# A Practical and Scalable-Oriented Enantioselective Synthesis of (*S*)‑Autumnaline

**DOI:** 10.1021/acs.oprd.5c00490

**Published:** 2026-06-25

**Authors:** Alessandro Brusa, Matteo Bottiglieri, Carola Tomassi, Francesco Feola, Daniele Lanaro, Andrea Bonetti, Gabriella Zappia, Federico Sala, Andrea Gambini, Mario Falzoni, Pietro Allegrini, Maria Luisa Gelmi, Massimiliano Aschi, Andrea Mazzanti, Fabio Pesciaioli, Armando Carlone

**Affiliations:** a Department of Physical and Chemical Sciences, Consorzio C.I.N.M.P.I.S., INSTM RU, Università degli Studi dell’Aquila, L’Aquila 67100, Italy; b Department of Pharmaceutical Sciences (DISFARM), 9304 Università degli Studi di Milano, Via Venezian 21, Milan I-20133, Italy; c Indena s.p.a., Via Don Minzoni 6, Settala, MI 20049, Italy; d Department of Industrial Chemistry “Toso Montanari”, University of Bologna, Via P. Gobetti 85, Bologna I-40129, Italy

**Keywords:** asymmetric synthesis, isoquinoline alkaloids, Bischler−Napieralski cyclization, circular dichroism

## Abstract

A process-friendly and enantioselective total synthesis of (*S*)-autumnaline, a key biosynthetic precursor to the colchicine alkaloids, is reported. The strategy relies on a Bischler–Napieralski cyclization–asymmetric imine reduction sequence to construct the chiral isoquinoline core. Significant process innovations include a cyanide-free homologation using a Darzens reaction and a chromatography-free purification strategy via d-tartrate salt formation. The absolute configuration was definitively confirmed through a combination of circular dichroism (CD) spectroscopy and computational modeling. This developed process affords (*S*)-autumnaline in 11 steps (nine-step longest linear sequence) with a 13% overall yield.

## Graphical abstract

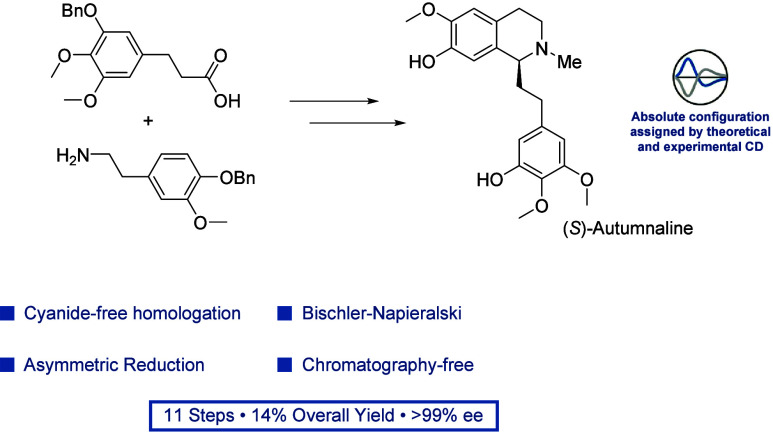

## Introduction

(*S*)-Autumnaline (**1**) (Figure 1), an isoquinoline alkaloid found in plants of *Colchicum* genus, is the biosynthetic precursor of colchicine and the related intermediated alkaloids involved in the biosynthetic cascade, such as isoandrocymbine, *O*-methylandrocymbine, demecolcine, and deacetylcolchicine.^1^

**Figure 1:**
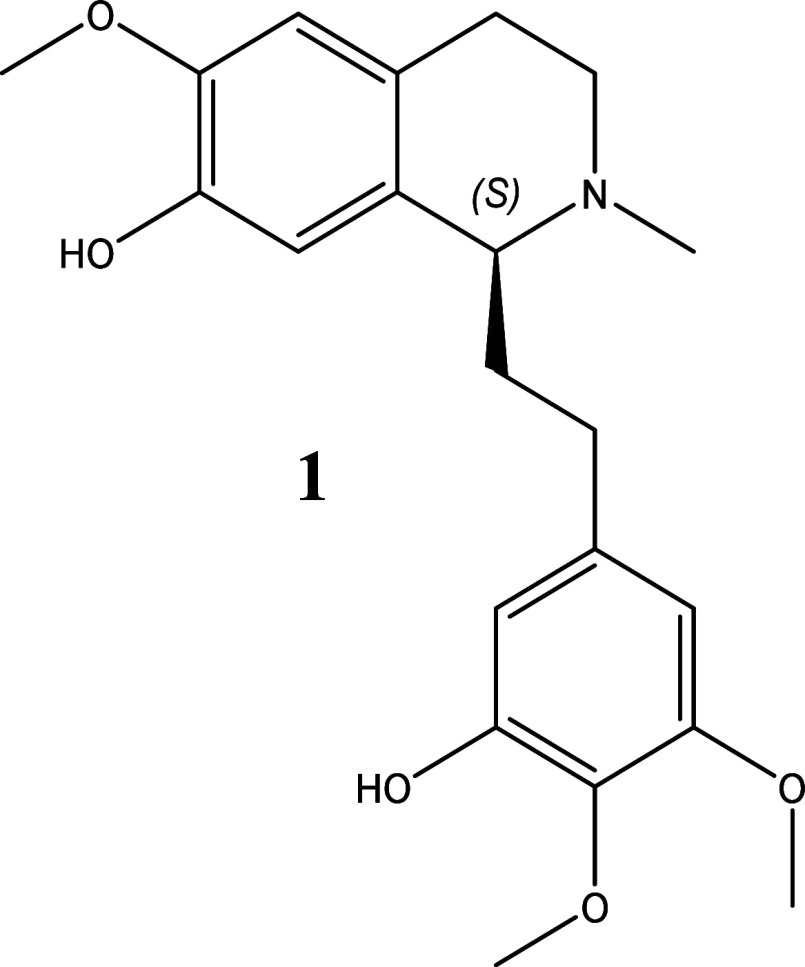
Structure of (*S*)-autumnaline (**1**).

It was extracted and isolated for the first time in 1969 by Šantavý and co-workers.^2^ The stereogenic center of the natural molecule was determined to be *S* by Barker and co-workers, via Cotton effect measured on the autumnaline *O*-dibenzyl tartrate salt.^3^ Until recently, autumnaline was prepared in racemic form and mainly for academic purposes—in particular for radiolabeling studies about *Colchicum* plant metabolism.^1,3–5^

To date, the only report for the enantioselective synthesis of **1** in appreciable quantities is a patented biocatalytic cascade via an engineered *Escherichia coli* strain, utilizing readily available and affordable starting materials, i.e., phenyl propionic acid and dopamine.^6,7^

Here, we engaged in developing an easy, practical, and enantioselective total synthesis of **1** developed with industrial considerations in mind, allowing us to have freedom to operate for business purposes. In fact, although the synthetic sequences previously reported are inspiring, they were not described in detail for industrial process and provided only racemic material.^1,3,8^ Furthermore, while the abovementioned chiral method offers an elegant solution to achieve enantiopure compound **1**, it requires specialized equipment or technology, which may present challenges for standard industrial facilities.

### Synthetic Strategy Evaluation

Upon considering various approaches for the synthesis of **1**, three distinct strategies were further evaluated on paper, based on key reactions for the synthesis of tetrahydroisoquinolines taking into consideration the possibility to achieve a chiral compound: the Bischler–Napieralski/asymmetric imine reduction sequence, the asymmetric Pictet–Spengler reaction, and the asymmetric Pomeranz–Fritsch–Bobbitt reaction followed by alkene hydrogenation (Scheme 1). The rationale was devised based on common disconnections so as to arrive at simple, readily available starting materials. The approach was selected to leverage established reactions often amenable to larger-scale operations that could be developed into a straightforward and scalable process. The asymmetric Pictet–Spengler reaction, reported on similar substrates with chiral phosphoric acids, was deemed unsuitable due to the high cost of the catalysts and the difficulty in optimizing them for our specific application.^9,10^ The Pomeranz–Fritsch–Bobbitt reaction was also dismissed, as the enantioselective organolithium addition to the imine intermediate is known to be a challenging and low-yielding step, making it unattractive for a scalable process.^11^

**Scheme 1:**
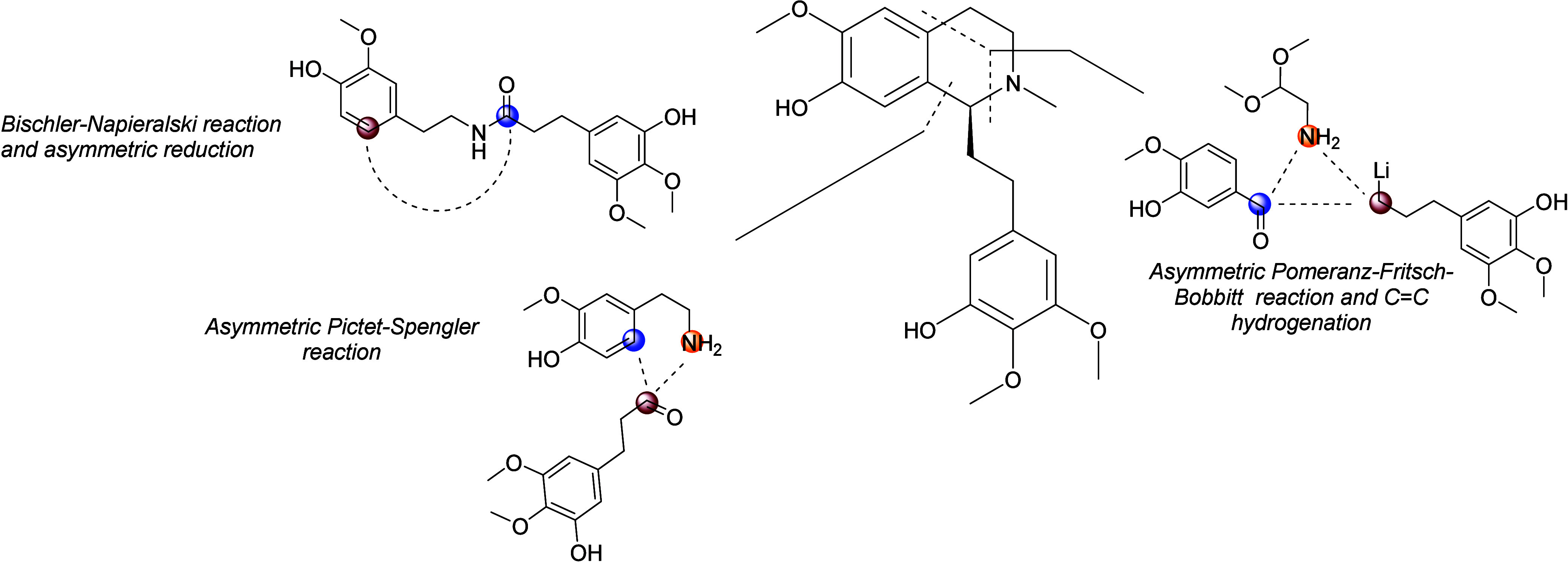
Tetrahydroisoquinoline Core Synthetic Approaches

The strategic selection of the Bischler–Napieralski reaction as the key cyclization step was pivotal to the synthetic design. This reliable and straightforward protocol efficiently yields the required dihydroisoquinoline intermediate via the annulation of an amide precursor. This choice directly influenced the starting materials required for the molecular core, thereby providing a clear and straightforward path for the rest of the synthetic disconnections (see Scheme 2).

**Scheme 2:**
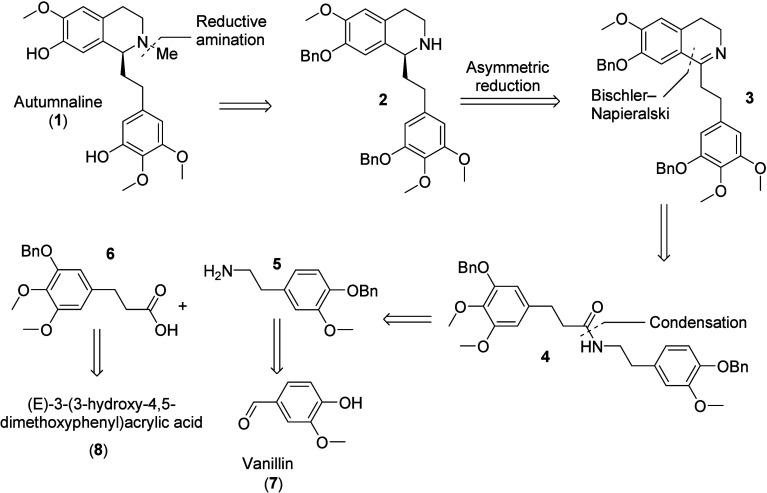
Retrosynthetic Approach to (*S*)-Autumnaline *via* Bischler–Napieralski Reaction

The first disconnection would be the removal of the *N*-methyl group to give intermediate **2**. Functional group interconversion (FGI) leads to **3**, via a reductive amination in the forward sense. The conversion of **3** into **2** via asymmetric reduction sets the stereogenic center toward the end of the synthesis. The dihydroisoquinoline ring can be closed via a Bischler–Napieralski reaction starting from **4**. Finally, amide bond disconnection yields **5** and **6**. Intermediate **5** can be prepared from vanillin (**7**) through a homologation/amination sequence. **6** can be easily traced back to (*E*)-3-(3-hydroxy-4,5-dimethoxyphenyl)­acrylic acid (**8**).

On the basis of the above synthetic scheme, here, we report on the development of an efficient and enantioselective total synthesis of **1**, avoiding the use of costly reagents. Our novel chemocatalytic approach shows potential for further scale-up in high overall yield affording a single stereoisomer of the target compound. The synthesis was designed to have an easy setup, avoid tedious chromatographic purifications, and utilize standard laboratory equipment, making it highly attractive for both academic and industrial settings. This work represents a significant advancement in the practical synthesis of this valuable alkaloid, providing an alternative to existing methods and offering a general blueprint for the synthesis of other complex isoquinoline alkaloids.

## Results

Different attempts were made to synthesize fragment **5**. All of the tested synthetic paths start from *O*-benzylated vanillin (**9**) to protect the sensible phenol group, diverging in the chemistry used to transform the aldehyde group **9** was obtained by reacting vanillin (**7**) with benzyl bromide in acetonitrile in the presence of potassium carbonate as a base. The benzyl group was selected due to its resilience and for the possibility to orthogonally deprotect the OH moieties. **9** was isolated in a 92% yield after crystallization in isopropanol (Scheme 3).

**Scheme 3:**
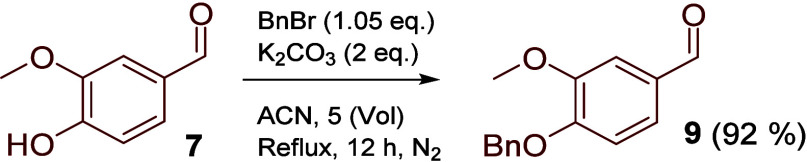
Benzylation of **7**

The reaction works also in methanol with slightly higher yield, but the use of acetonitrile was preferred because of the absence of side benzylation processes, which make the purification easier.

Homologation of the aldehyde group of **9** would add one more carbon atom to the chain and the terminal nitrogen atom, giving access to intermediate **5**. The most popular ways of doing this are the addition of cyanide after aldehyde reduction and subsequent reduction of the nitrile group, Henry reaction with nitromethane and reduction of the corresponding nitroalkene **10** to amine, Darzens reaction to form the glycidic ester, decarboxylation and trapping of the aldehyde to form the oxime **11**, and subsequent reduction to amine (Scheme 4). A route employing the use of cyanide was obviously ruled out.

**Scheme 4:**
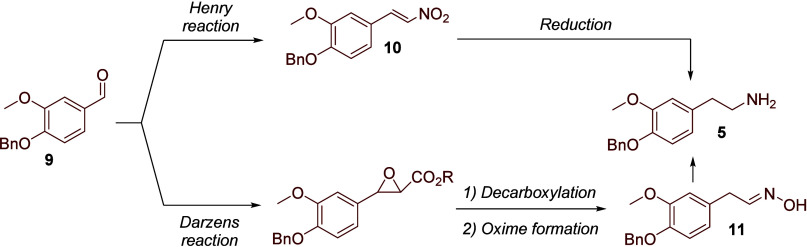
Possible Paths to Obtain **5** from **9**

To avoid the formation of potentially explosive methane nitronate ion formed under basic conditions, the Henry condensation of **9** with nitromethane was carried out in a neat acid environment,^12^ isolating **10** in 94% yield. Nitroalkene **10** was then reduced to amine **5** by lithium aluminum hydride in THF in a 65% isolated yield (Scheme 5).

**Scheme 5:**
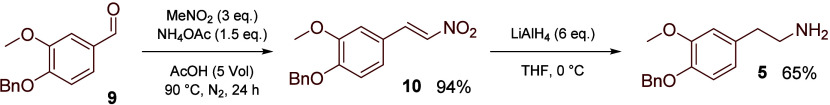
Synthesis of **10** and **5** from **9**

Purification of **5** by column chromatography was needed to remove dimer **5** ^**I**^ and trimer **5** ^**II**^ formed, under the above reaction conditions, in significant amounts. A plausible mechanism for their formation is the attack of the *in situ*-generated primary amine on a common imine-like intermediate in the presence of a Lewis acid followed by reduction (Scheme 6).

**Scheme 6:**
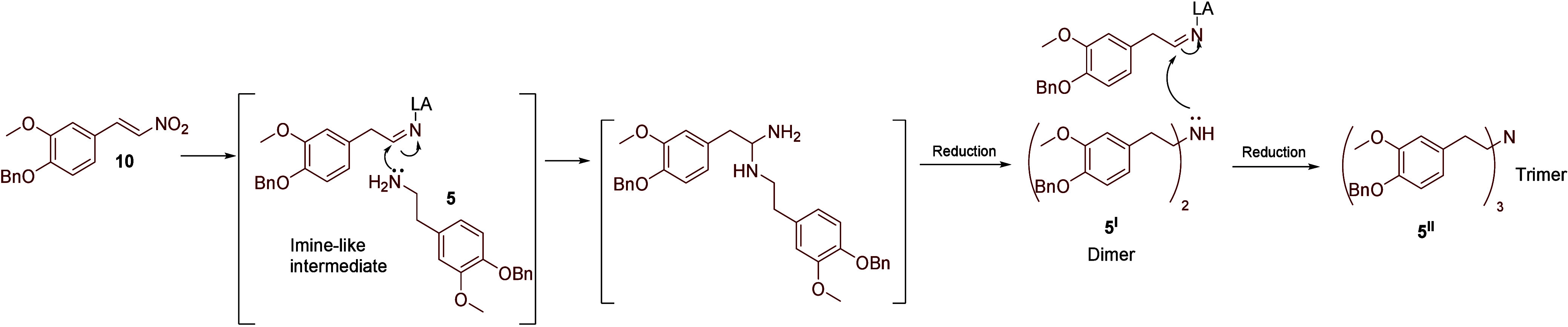
By-product Formation during the Synthesis of **5**

Alternative reduction strategies for the conversion of **10** to **5** were also investigated with discouraging results, including catalytic hydrogenation over palladium on carbon and a stepwise sequence involving reduction of the alkene moiety to the corresponding nitroalkane, followed by nitro-group reduction (see the Supporting Information for further details).

The path proceeding via Darzens reaction was then explored. First, **9** was exposed to chloro-methyl acetate in methanol in the presence of sodium methoxide as base. The glycidic ester product **12** was obtained quantitatively, by self-precipitation. Subsequently, **11** was converted to oxime **11** in a two-step, one-pot sequence. In particular, in the initial step, a saponification reaction with potassium hydroxide, quantitatively yielded the potassium carboxylate **12** ^**I**^. This intermediate was then carried forward in a tandem process where the addition of hydroxylamine hydrochloride (NH_2_OH•HCl) triggered a tandem glycidic acid decarboxylation^13^ and oxime formation, leading to **11**. The reaction conditions were carefully optimized to ensure a high yield. A slight excess of potassium hydroxide (KOH, 0.1 equiv) was used to ensure complete conversion and provide additional base to deprotonate a portion of the NH_2_OH•HCl. This carefully controlled, buffered system ensures a continuous excess of free hydroxylamine, which is crucial for promoting the conversion of the unstable aldehyde intermediate **12** ^**II**^ into the corresponding oxime **11**. Furthermore, the direct introduction of hydroxylamine into the reaction mixture effectively prevents the self-aldol condensation of the phenyl-acetaldehyde-derived intermediate, a side reaction known to occur spontaneously with these substrates.^14^ This strategy proved highly effective, furnishing compound **11** in an 85% yield after crystallization from methanol (Scheme 7).

**Scheme 7:**
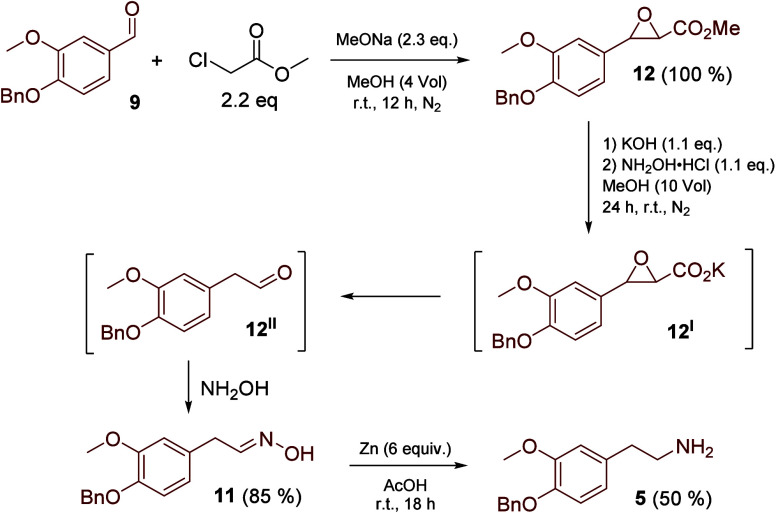
Synthesis of the Amine Fragment **5**—Oxime Route

A selection of reducing agents was tested on **11**, such as lithium aluminum hydride^15^ or zinc in the presence of ammonium formate,^16^ resulting in the formation of significant amounts of **5** ^**I**^ and **5** ^**II**^, analogously to what was observed for nitroalkene **10** (Table 1). Reduction of oxime **11** with zinc in the presence of acetic acid concurrently protonates product **5** reducing its nucleophilicity, drastically decreasing any significant byproduct formation (entry 3); reducing the amount of zinc or acetic acid does not suppress the formation of byproducts (entry 4). Other reduction methodologies were discarded for our purposes.^17–19^ The crude mixture was purified by simple filtration and partitioned between toluene and 10 M sodium hydroxide in water to remove the zinc salts, affording the free amine in 50% yield. The unexpected moderate yield is due to the tendency of **11** to coprecipitate with potassium chloride in various amount, lowering the real compound title.

**Table 1:** Screening of Reducing Agents to Access Compound **5**

entry	substrate	reducing agent	conditions	results
1	**11**	Zn	HCOONH_4_, MeOH, reflux	**5** ^**I**^ **, 5** ^**II**^
2	**11**	LiAlH_4_	THF, reflux	**5, 5** ^**I**^ **, 5** ^**II**^
3	**11**	Zn (6 equiv)	AcOH (15 vol), r.t.	**5** (50%)
4	**11**	Zn (3 equiv)	AcOH, r.t.	**5** ^**I**^, byproducts (not characterized), **5**

In view of the chromatographic purification required for lithium aluminum hydride reduction, the evolution of hydrogen gas, and the laborious workup, reduction with zinc in acetic acid was preferred to facilitate future scale-up of the process. For these reasons, the oxime route described in Scheme 7 was selected.

Carboxylic acid fragment **6** was synthesized by starting from commercially available acid **8**. The starting material was readily hydrogenated using palladium on carbon in quantitative yield. Crude **13**, obtained after filtration of palladium on carbon over Celite and solvent evaporation, was subjected to the benzylation of the free hydroxyl group. To achieve an efficient benzylation of **13** in methanol, a strong excess of benzyl bromide (2.5 equiv) and K_2_CO_3_ (2.6 equiv) is required. The product was isolated in 90% yield with the excess of the corrosive and irritating benzyl bromide remaining unreacted in the crude mixture. On the other hand, carrying out the reaction in acetonitrile, the excess of benzyl bromide was consumed, because the benzyl ester of **13** was formed, providing a better impurity safety profile. Even if the ester byproduct was formed, the saponification of the reaction mixture with potassium hydroxide in a water–acetonitrile mixture and quenching with an acidic workup provided product **6** by filtration in 92% yield over the two steps (Scheme 8).

**Scheme 8:**
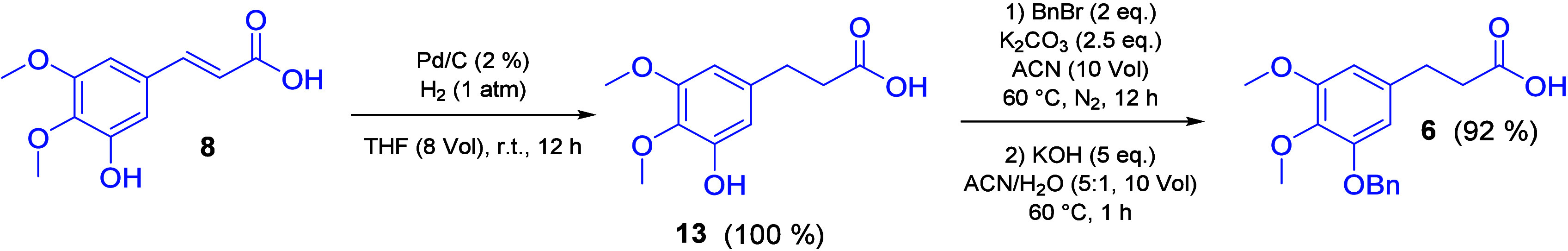
Synthetic Sequence for Carboxylic Acid Fragment **6**

Fragments **5** and **6** were combined to construct the tetrahydroisoquinoline core and complete the final steps of the synthesis (Scheme 8). Following a screening of coupling conditions for the condensation of fragments **5** and **6** to form intermediate amide **4** (Table 1 in the Supporting Information (SI)), CDI (carbonyldiimidazole) in ethyl acetate was finally chosen. Crystallization in isopropanol furnished amide **4** in a 75% yield. Compound **4** was very rapidly converted to the key imine intermediate **3** in the presence of an excess of phosphorus trichloride (POCl_3_) in toluene, after easy removal of excess POCl_3_ washing with a basic aqueous solution or direct evaporation. Imine **3** could be used immediately in the subsequent enantioselective reduction step without further purification (Scheme 9).

**Scheme 9:**
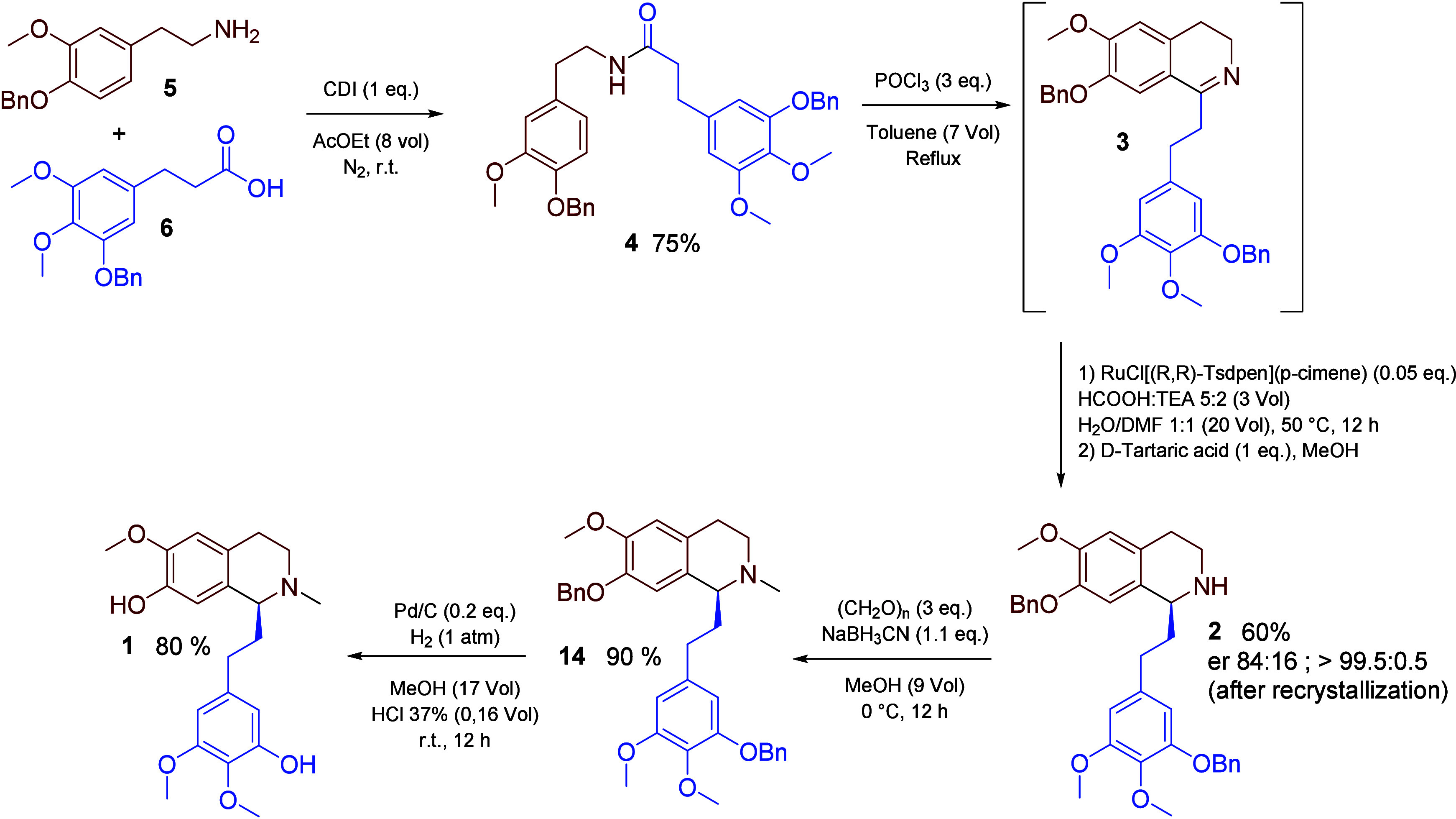
End Game toward **1**

An asymmetric transfer hydrogenation (ATH), using 5 mol % of RuCl­[(*R*,*R*)-Tsdpen]­(p-cimene) as a precatalyst and formic acid as reducing agent, was screened as a promising approach, inspired by the work of Puerto Galvis and Kouznetsov (Table 2).^20^

**Table 2:** Selected Optimization Conditions of the Asymmetric Transfer Hydrogenation (ATH)

entry	**catalyst loading** ^a^	solvent	reducing agent	**yield** ^b^	e.r.^c^
1	2%	H_2_O/MeOH 1:1 (100 vol)	HCOOH/TEA^d^ 5:2 (10 equiv)	40%	66:34
2	2%	H_2_O/DME^e^ 1:1 (20 vol)	HCOOH/TEA 5:2 (10 equiv)	50%	62:38
3	2%	H_2_O/NMP^f^ 1:1 (20 vol)	HCOOH/TEA 5:2 (10 equiv)	20–25%	53:47
4	5%	H_2_O (20 vol)	HCOOH/TEA 5:2 (10 equiv)	byproduct formation	n.d.
5	2%	H_2_O/DMF 1:1 (24 vol)	HCOOH/TEA 5:2 (10 equiv)	88%	84:16
6	2%	H_2_O/DMF 1:1 (11 vol)	HCOONa (5 equiv)	30%	87:13
7	2%	H_2_O/DMF 1:1 (100 vol)	HCOOH/TEA 5:2 (10 equiv)	>95%^g^	66:34
8	5%	H_2_O/DMF 1:1 (10 vol)	HCOOH/TEA 5:2 (10 equiv)	60% + 20% byproduct formation	n.d.
9	5%	H_2_O/DMF 1:1 (20 vol)	HCOOH/TEA 5:2 (1 equiv)	full conversion with concomitant byproduct formation	n.d.
10	5%	H_2_O/DMF 1:1 (20 vol)	HCOOH/TEA 5:2 (10 equiv)	>95%	84:16

a The catalyst was loaded in DMSO solution.

b Measured by ^1^H NMR.

c er. = enantiomeric ratio, calculated by chiral HPLC.

d TEA = triethylamine.

e DME: dimethoxyethane.

f NMP: *N*-methylpyrrolidone.

g Byproduct detected.

The optimized reaction conditions were established to ensure high reproducibility, selectivity, favorable rheology, and complete conversion of **3**. Among the different solvents tested (entries 1–5), 1:1 H_2_O/DMF (entry 5) showed the best conversion and chemoselectivity. Alcohols, including methanol and *tert*-butanol, were also investigated as reaction media and demonstrated an initial efficacy on a small scale. However, upon performing the reactions on a 100 mg scale, their performance exhibited poor reproducibility, leading to the ultimate selection of DMF-based systems (further details are provided in the Supporting Information).^20^ Reducing agent HCOOH/TEA (5:2; entry 5) performed better than sodium formate (entry 6). Indeed, altering concentration, catalyst loading, and amount of reducing agent (entries 5–9) did not improve the outcome. Finally, a small increase in catalyst loading and reduced solvent volumes (entry 10) ensured high conversion of **3** (>95%), affording **2** with an enantiomeric ratio that, although not great, is the optimal compromise for the designed process. These conditions were successfully reproduced in the lab on a 15 g scale.

Presumably, steric clashes introduced by the benzyl ethers affects key substrate–catalyst interactions, resulting in lower enantioselectivity than that observed by Kouznetsov and co-workers for a structural analogue (Scheme 10). Additionally, the uncatalyzed reduction of imine **3** proceeds, under the same conditions, in 30% conversion; this would further contribute to an erosion of the enantiomeric purity.

**Scheme 10:**
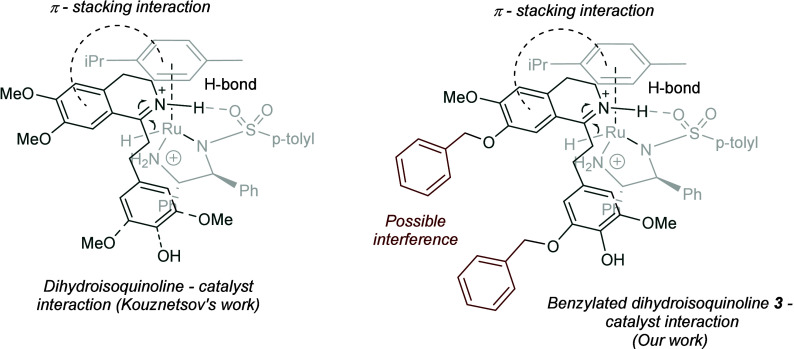
Ruthenium Hydride Complex Interaction with the Substrate

Crystallization of **2** was pursued for enantio-enrichment; both racemic and scalemic mixtures were screened under various conditions (see the SI). d-Tartaric acid in methanol proved to be the most effective, causing precipitation of the hemitartrate salt and yielding the product in enantiopure form.^3,21^ After treatment of the salt with a base, compound **2** was isolated in enantiopure form in 60% overall yield over two synthetic steps and a recrystallization.

Once the chiral isoquinoline core was established, the final steps of the synthesis were achieved through a series of straightforward chemical transformations, starting from a reductive amination with formaldehyde. Using sodium cyanoborohydride as a reducing agent and para-formaldehyde as a formaldehyde source in methanol at 0 °C followed by a simple acqueous workup, tertiary amine **14** was obtained in 90% yield. Luckily, no racemization was observed in these conditions as reported by Lin and Ma^22^, Puerto Galvis and Kouznetsov,^20^ and others.^23,24^ Finally, hydrogenolysis of the benzyl group provided the enantiopure desired target **1** in 80% yield, after simple filtration and a basic wash. Noteworthily, concentrated aqueous hydrogen chloride was used as an additive to protonate the amine functionality, effectively preventing the product from absorbing onto the catalyst surface. Furthermore, chiral HPLC confirmed that no epimerization occurred during the final stages of the synthesis.

### Determination of the Absolute Configuration via Circular Dichroism

A critical aspect of our program was the determination of the absolute configuration of the final product obtained. Access to a natural reference sample of **1** or *Colchicum* plant for extraction was unfeasible. Therefore, the stereochemical outcome of the presented enantioselective synthesis was confirmed via circular dichroism (CD). A CD spectrum was first recorded on a sample synthesized using the (*R*,*R*) catalyst. Concurrently, a high-level computational simulation of the CD spectrum for (*S*)-**1** was performed, employing a quantum–classical methodology as described below. The structure of (*S*)-**1** was simulated for 60 ns using classical molecular dynamics (MD) in an acetonitrile box at a temperature of 25 °C and a pressure of 1.0 bar. The GROMACS software package^25^ was employed with the GROMACS-ATB force field.^26^ From the MD trajectory, five representative structures were extracted, each associated with a corresponding probability. Subsequently, density functional theory (DFT) calculations were performed on these selected structures. Specifically, for each structure, (i) geometry optimizations were carried out while constraining the soft degrees of freedom (mainly torsional angles) in order to preserve the conformations sampled during MD; (ii) on the optimized geometries, time-dependent DFT (TD-DFT) calculations were conducted using the B3LYP functional^27,28^ and the 6-311+G** basis set, including solvent effects via the polarizable continuum model (PCM)^29^ as implemented in Gaussian16,^30^ the software used for the TD-DFT computations. A total of 65 vertical excited states were determined. Finally, the absorption spectrum was obtained by applying Gaussian broadening functions with a σ value of 0.090 eV. The final spectrum was generated by summing the individual spectra, each weighted according to the probability derived from the MD simulation (see the SI).

The exceptional alignment between the experimental and simulated data (Figure 2) provides compelling and unambiguous evidence that the process successfully delivers the target molecule with the correct (*S*)-stereochemistry. There is a good fit between the experimental and observed spectra, particularly in the polarity inversion region. This result allows for the definitive conclusion that the (*R*,*R*) enantiomer of the catalyst yields (*S*)-**1**. This finding also aligns with previous results reported by Puerto Galvis and Kouznetsov in their synthesis of the *Dysoxylum* alkaloid series.^20^ This confirmation underscores the rigor, reliability, and precision of the process, making it a valuable addition to the field of process chemistry. This not only validates the synthetic strategy but also corroborates a powerful and practical method for stereochemical assignment in the absence of natural product standards, which is a challenge often encountered in complex natural product synthesis.

**Figure 2:**
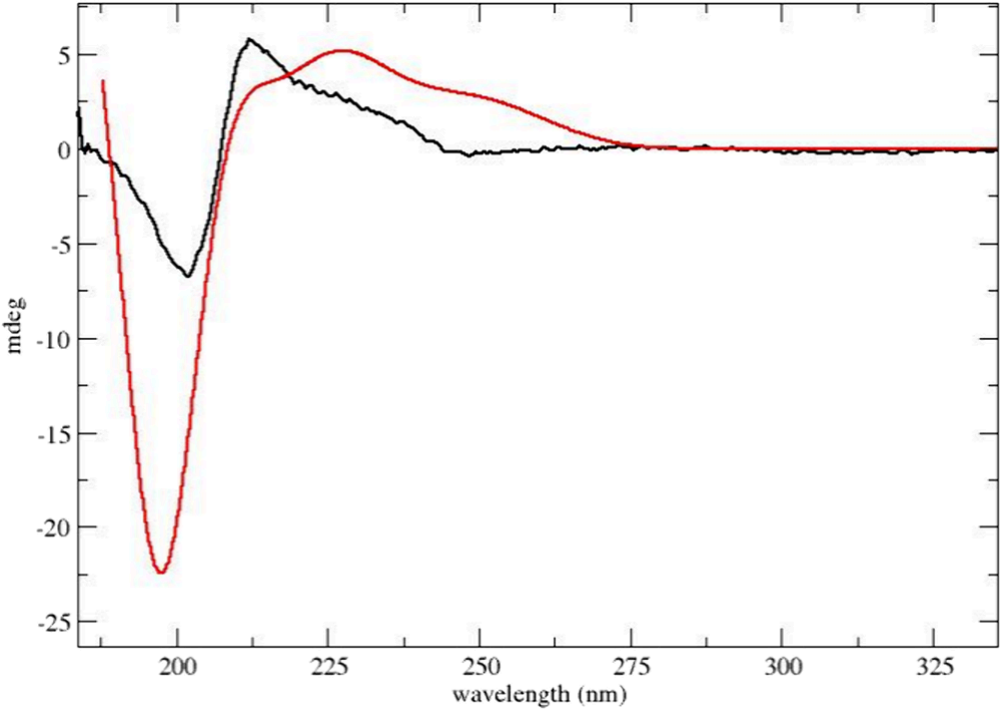
Overlap of experimental (black line) and simulated (red line) CD.

## Conclusions

In summary, this work establishes a practical route with potential for industrial adaptation to (*S*)-autumnaline, effectively overcoming the lack of stereocontrol inherent in the previous academic methods. The synthetic strategy centers on a Bischler–Napieralski cyclization followed by an asymmetric imine reduction to install the tetrahydroisoquinoline core. To ensure process safety and cost efficiency, we engineered a cyanide-free homologation sequence utilizing a Darzens reaction and a tandem decarboxylation-oxime formation, avoiding the need for hazardous reagents or expensive catalysts. Furthermore, critical impurity issues during the reduction step were resolved by optimizing the reaction environment, while downstream processing was significantly streamlined through a chromatography-free purification strategy using d-tartaric acid. Beyond the process improvements, a rigorous confirmation of the absolute configuration via circular dichroism (CD) spectroscopy and computational modeling provided unambiguous evidence of the correct (*S*) stereochemistry, validating a powerful and practical method for stereochemical assignment in the absence of natural product standards. Ultimately, this efficient 11-step sequence, with a longest linear sequence of nine steps and an overall yield of 13%, offers a competitive chemocatalytic alternative, amenable to larger-scale laboratory preparation, to existing methods and provides a generalizable blueprint for the sustainable manufacture of complex isoquinoline alkaloids.^31^

## Supplementary Material



## Notes

Synthetic procedures, characterization, ECD, NMR and HPLC spectra, and ECD simulation data (PDF)
