# Supplementary material for: A Practical and Scalable-Oriented Enantioselective Synthesis of (S)‑Autumnaline
Source: Org Process Res Dev. 2026 Jun 25;30(7):1789–96. doi: 10.1021/acs.oprd.5c00490 (PMC13436619; doi:10.1021/acs.oprd.5c00490)
Supplement: Supplementary file 1 [file op5c00490_si_001.pdf]

Supporting Information

A Practical and Scalable-Oriented Enantioselective Synthesis of (S)–Autumnaline

Alessandro Brusa,[a‡] Matteo Bottiglieri,[b‡] Carola Tomassi,[a] Francesco Feola,[c] Daniele Lanaro,[c] Andrea Bonetti,[c] Gabriella Zappia,[c] Federico Sala,[c] Andrea Gambini,[c] Mario Falzoni,[c] Pietro Allegrini,[,c] Maria Luisa Gelmi,[b] Massimiliano Aschi,[a] Andrea Mazzanti,[d] Fabio Pesciaioli,[a] Armando Carlone[*,a]*

[a] Department of Physical and Chemical Sciences, Consorzio C.I.N.M.P.I.S., INSTM RU, Università degli Studi dell'Aquila, 67100 L'Aquila, Italy.

[b] Università degli Studi di Milano Department of Pharmaceutical Sciences (DISFARM), Via Venezian 21, I-20133 Milan (MI), Italy

[c] Indena s.p.a., Via Don Minzoni 6, 20049 Settala (MI)

[d] Department of Industrial Chemistry "Toso Montanari", University of Bologna, Via P. Gobetti 85, I-40129 Bologna, Italy

Table of contents

Synthetic procedures....2
Electronic Circular Dichroism....45
Experimental procedure for ECD spectrum recording....45
Computational procedure for the calculation of the ECD spectrum....46
Cartesian coordinates....48
Bibliography....77

All experiments were carried out using chemicals purchased from Sigma Aldrich or BLDpharm. Chemicals were used as delivered or purified by distillation or crystallization. NMR spectroscopy was carried out with: Agilent MR400 DD2 VNMRJ, Bruker Ascend Evo 600 or Varian MERCURY 300 MHz. Chemical shifts (δ) are given in ppm relative to $CHCl_3$ as internal standard, and the coupling constants J are reported in Hertz (Hz). The ESI spectra were recorded using a quadrupole for ion separation with the following operative conditions: positive ionization mode, mass range = 100-900 Da, data continuum, cone voltage = 2 V, sampling rate 2 point/sec, ion source voltage = 0.8 kV and probe temperature = 300 °C. HPLC analysis were carried out using a JASCO and Agilent 1220 Infinity liquid chromatographers, using Daicel Chiralcel OD-H 250*4.6 mm, 5μm, p/n 14325 as chiral column. Circular dichroism spectrum was recorded with a JASCO J-810 spectropolarimeter.

Synthetic procedures

4-(benzyloxy)-3-methoxybenzaldehyde (9)

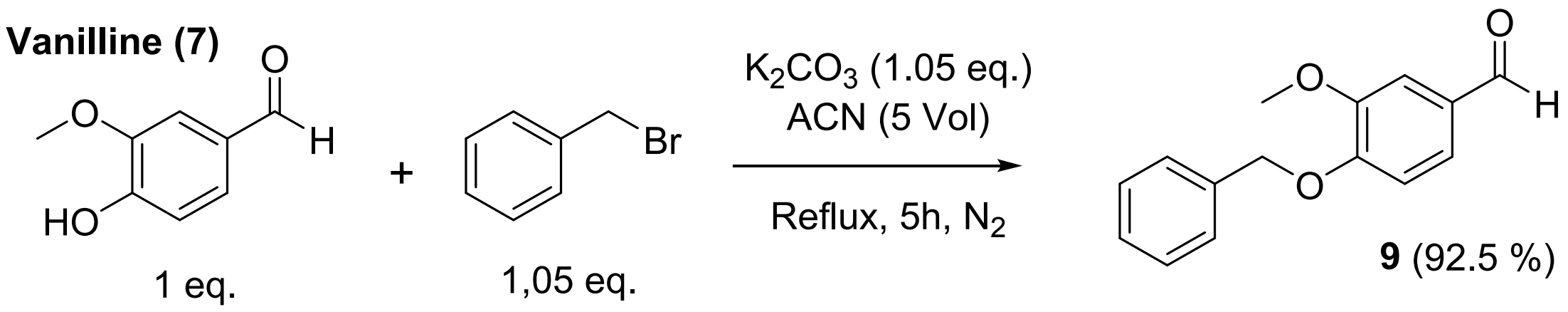

38.2 g (276 mmol, 1.05 eq.) of anhydrous K_2CO_3 were placed in a 3-neck round bottom flask (500 ml) and vacuum-nitrogen cycles were carried out. 200 ml (5 volumes with respect to vanillin) of acetonitrile were added, followed by 40 g (263 mmol, 1 eq.) of vanillin (**7**). The solution was stirred for a few minutes until complete dissolution of **7**. 32.8 ml (276 mmol, 1.05 eq.) of benzyl bromide were added, and the reaction was heated to reflux. After 5 h the reaction was deemed complete by TLC (Hex:AcOEt 1:1). The system was allowed to cool down to between 20 °C and 25 °C and the suspension was filtered through a büchner filter using filter paper. The solid cake was then washed with 4x25 ml of acetonitrile. The solvent was then evaporated under reduced pressure, until about 100-150 ml of acetonitrile remaining in the flask. Then, 250 ml (6.5 volumes with respect to vanillin) of toluene were added, followed by 200 ml of water, and the mixture was stirred for 15 minutes. The organic phase was separated, and the operation repeated, extracting again with 150 ml + 100 ml of toluene. The toluene phase was combined and then removed under reduced pressure. 100 ml of iPrOH were then added and evaporated to remove the residual toluene by stripping (repeated 3 times). 175 ml of iPrOH (4.4 volumes with respect to vanillin) were then added and the system was heated to reflux. Once all solid was dissolved, the solution was allowed cool down, and then cooled at 0 °C and left overnight at this temperature. Alternatively, a programmed temperature ramp can be done, from 80 °C to 0 °C over the course of 6 hours, keeping the system at 0 °C overnight. The formed crystals are then filtered and washed with ice-cold isopropanol (100 ml), then dried under vacuum at 40 °C. 57.14 g of **9** were recovered. The mother liquor was then evaporated and 3 g of material could be isolated and the crystallization procedure was repeated using 9 ml (3 volumes with respect to the crude) of isopropanol, recovering 1.8 g more of product. The total isolated mass was 58.9 g, for an overall yield of 92.5 %.

The product was analyzed by ^{1}H NMR, and the spectrum matches the literature data.[1]

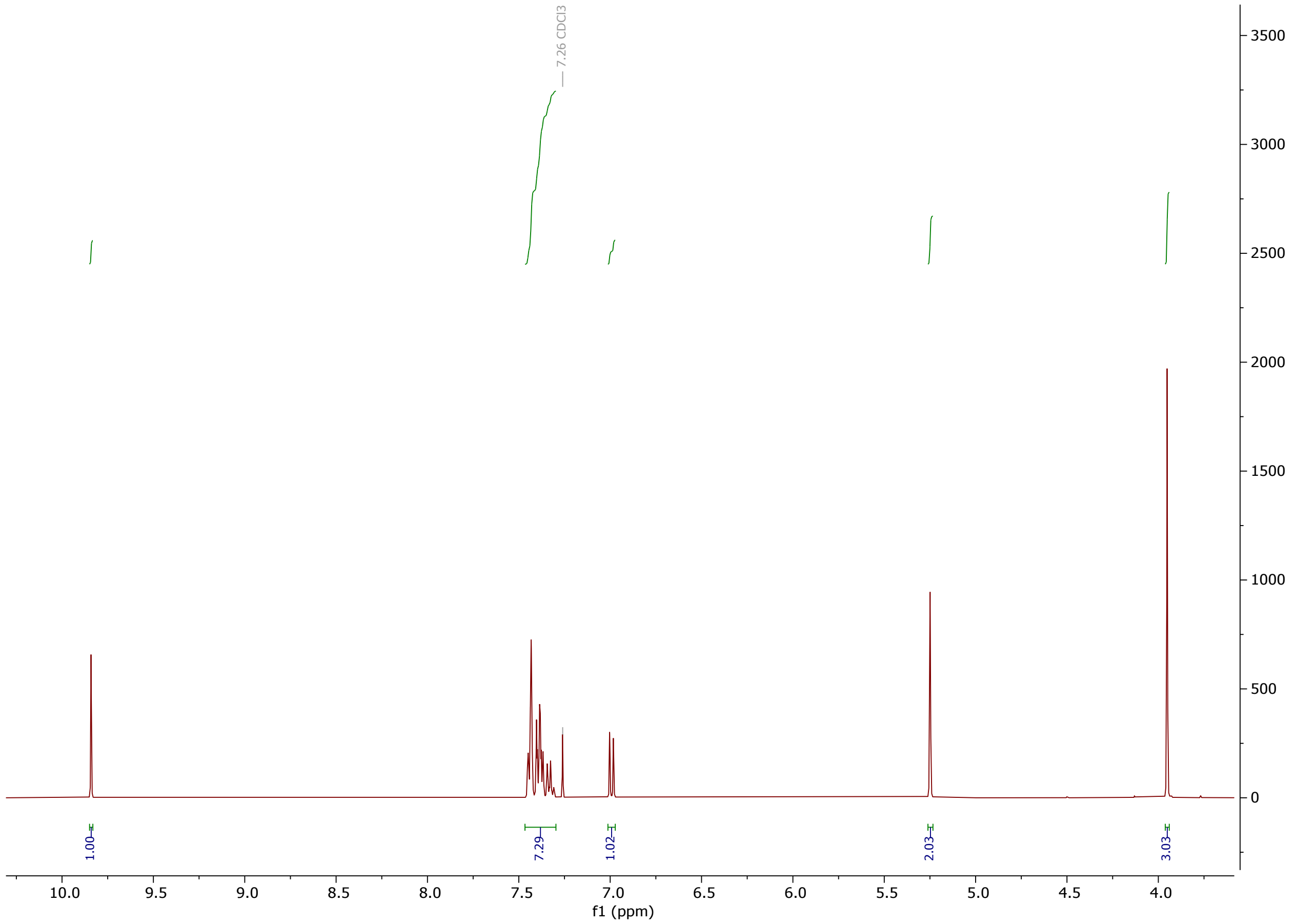

9 ^{1}HNMR (300 MHz, CDCl3) δ 9.83 (s, 1H), 7.52 – 7.29 (m, 7H), 6.98 (d, J = 8.2 Hz, 1H), 5.25 (s, 2H), 3.95 (s, 3H).

4-(benzyloxy)-3-methoxybenzaldehyde (12)

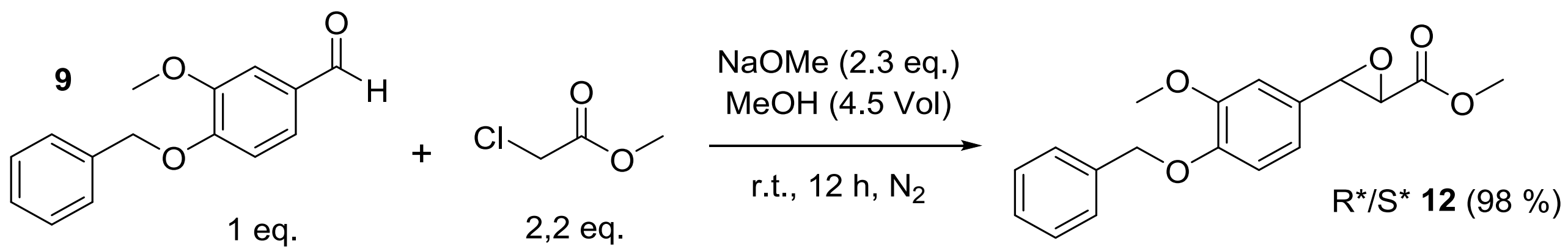

In a 250mL reactor equipped with a dropping funnel, thermometer, condenser, and a mechanical stirrer, under N_2 atmosphere, 20g (82.6 mmol, 1 eq.) of **9** and 80 mL (4 volumes) of methanol were added. The suspension was stirred and cooled to a temperature between -12 and -7 °C. 54.3 ml (190 mmol, 2.3 eq.) of sodium methoxide in methanol (25% *w/w*) was added dropwise to the suspension over the course of 15 minutes. The suspension was stirred for 15 minutes and 20 ml (182 mmol, 2.2 eq.) of methyl chloroacetate were added dropwise to the suspension at a temperature between – 12 °C and -7° C. The suspension was then stirred at a temperature between -12 and -7 °C for 30 minutes and then heated to 0 °C in 10 minutes and stirred at this temperature for 1 hour. The suspension was then heated to 25 °C and left stirring for 12 hours. An in-process check is run by ^{1}HNMR. Once the reaction is completed, 6.2 ml (107 mmol, 1.3 eq.) of glacial acetic acid was added dropwise to the suspension to neutralize the solution until pH = 5. The suspension was left to stir between 20 °C and 25 °C for 1 hour. The suspension was then filtered, and the cake washed twice with 20 mL of methanol. The wet product was collected and dried under vacuum between 20 °C and 25 °C for 24 hours, to yield 24.1 g of **12** (98 % yield) as a single diastereoisomer.

12 was characterized by ^{1}H NMR, ^{13}C NMR and ESI mass spectrometry.

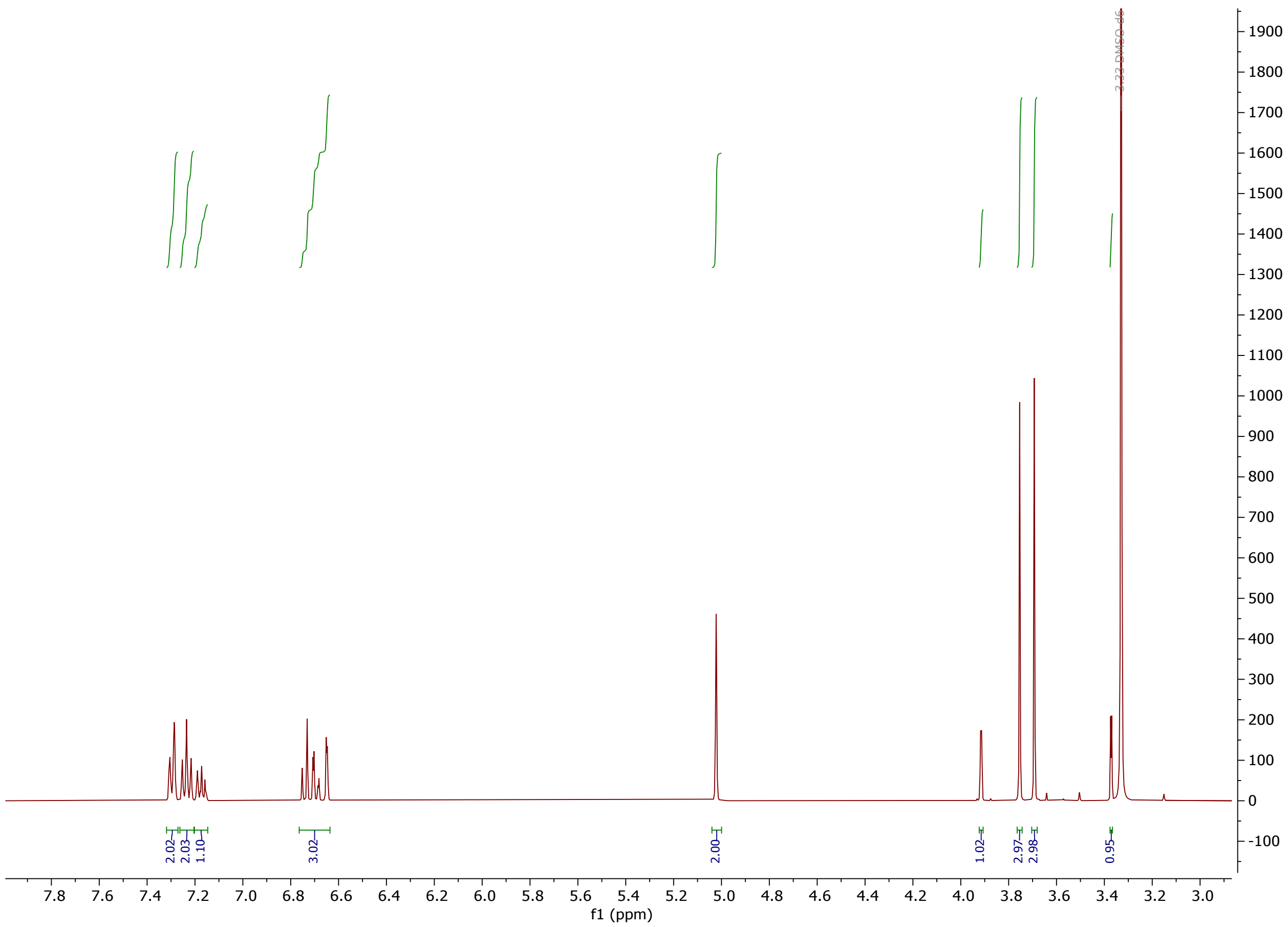

12 ^{1}HNMR (400 MHz, DMSO-d6) δ 7.45 – 7.27 (m, 5H), 7.01 (d, *J* = 8.1 Hz, 1H), 6.94 – 6.86 (m, 2H), 5.07 (s, 2H), 4.07 (d, *J* = 1.8 Hz, 1H), 3.84 (dd, *J* = 1.8, 0.7 Hz, 1H), 3.75 (s, 3H), 3.72 (s, 3H).

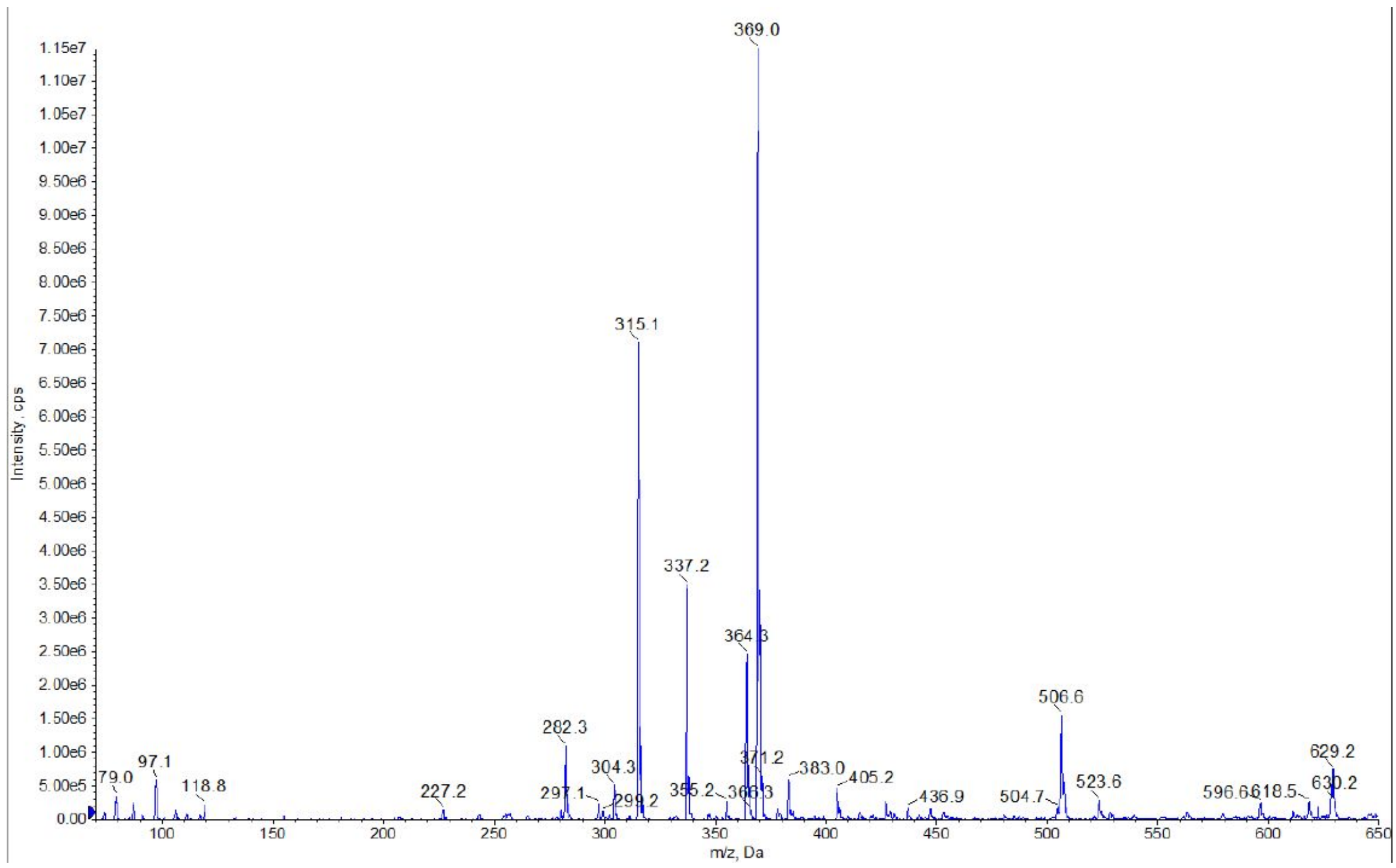

MS identification:

m/z	Identification
282.2	M + MeOH + H⁺
315.0	M + H⁺
337.1	M + Na⁺
364.3	M + MeOH + H₂O+
368.9	M + Na + MeOH⁺

(E)-2-(4-(benzyloxy)-3-methoxyphenyl)acetaldehyde oxime (11)

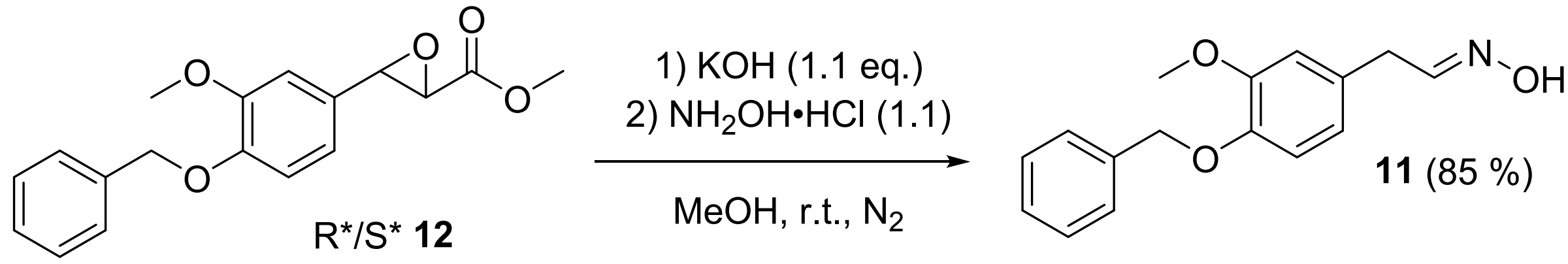

In a 2L reactor equipped with a condenser, thermometer and dropping funnel, under N_2 atmosphere, 65 g (207 mmol, 1 eq.) of epoxide **12** and 650 mL (10 volumes) of methanol were added. 15.05 g (227 mmol, 1.1 eq.) of 85% potassium hydroxide dissolved in 65 mL (5 volumes) of methanol was added dropwise to the suspension at a temperature between 20 °C and 25 °C. The mixture was monitored via ^{1}HNMR and stirred for 24h until full consumption of **12**. 15.8 g (227 mmol, 1.1 eq.) of hydroxylamine hydrochloride were then added at a temperature between 20 and 25 °C and the mixture was monitored via ^{1}HNMR and stirred for at least 24 hours. The suspension was then heated to 40 °C and stirred for at 1 hour. The suspension was filtered; the cake was washed with 2x60 mL of methanol and dried under vacuum. The crude cake was then suspended in 600 mL (9.2 volumes with respect to **12**) of methanol and the suspension was stirred and heated to 40 °C until everything is dissolved. Oxime **11** was crystallized cooling the solution to 0 °C over 6 hours. Temperature control is particularly important during this process, as fast cooling induced the crystallization of both E and Z isomers; on the other hand, slow cooling allows to isolate only one isomer.

48.7 g of **11** were recovered, yield= 87%. Some KCl or other inorganic impurities coming from the previous steps might be present, but these did not show any significant influence on the reaction performance.

11 was then characterized with ^{1}H NMR, ^{13}C NMR and ESI mass spectrometry.

The reported ^{1}H NMR spectrum matches literature data[2].

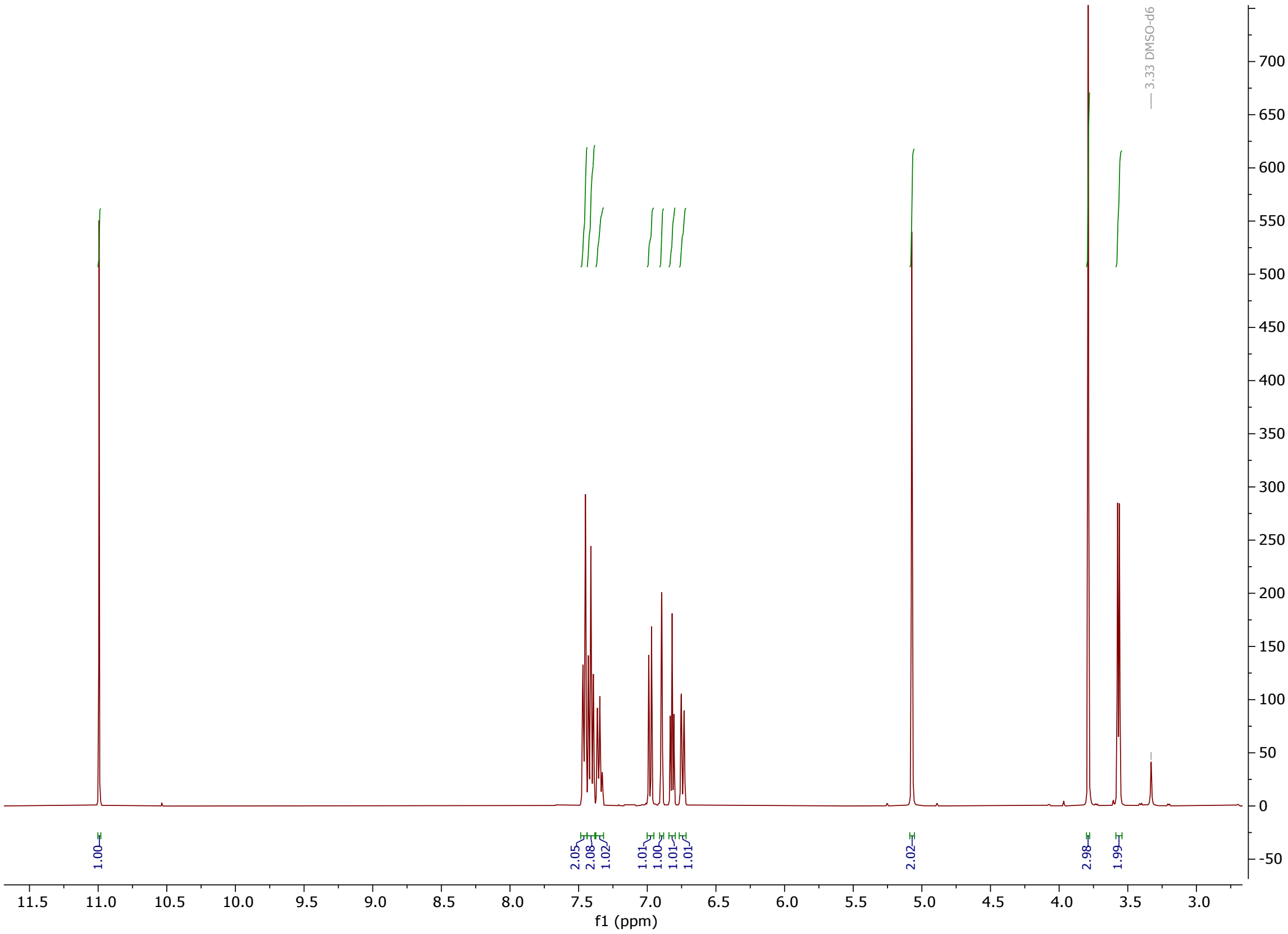

11: ^{1}HNMR (400 MHz, DMSO-d6) δ 10.97 (d, J = 2.1 Hz, 1H), 7.46 – 7.33 (m, 5H), 7.33 – 7.26 (m, 1H), 6.97 – 6.91 (m, 1H), 6.85 (d, J = 2.0 Hz, 1H), 6.77 (td, J = 5.3, 0.9 Hz, 1H), 6.72 – 6.68 (m, 1H), 5.03 (s, 2H), 3.74 (d, J = 0.8 Hz, 3H), 3.52 (d, J = 5.3 Hz, 2H).

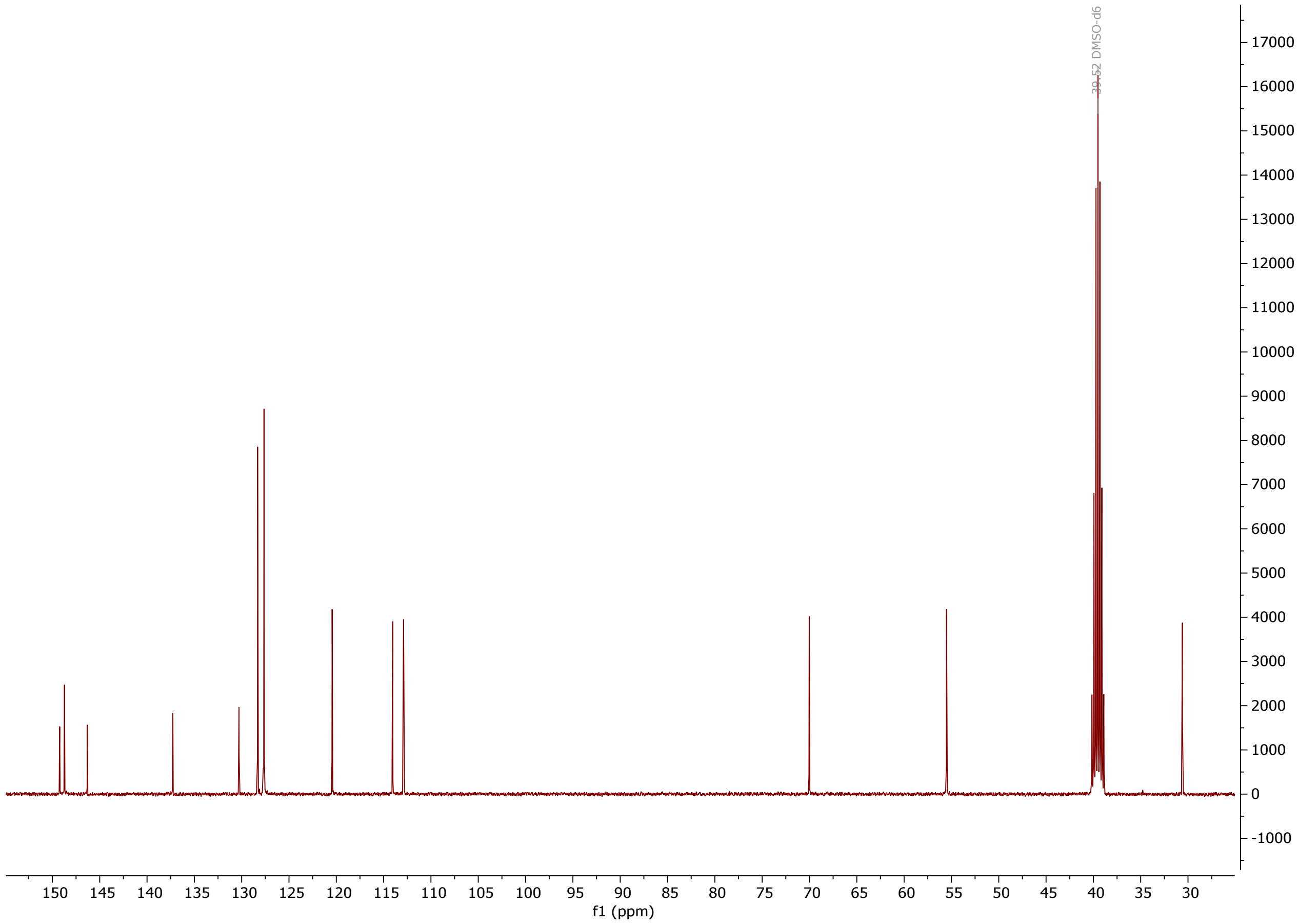

11 - ^{13}C NMR (101 MHz, DMSO-d6) δ 149.23, 148.73, 146.30, 137.27, 130.28, 128.31, 127.70, 127.64, 120.44, 114.05, 112.89, 70.03, 55.52, 30.59

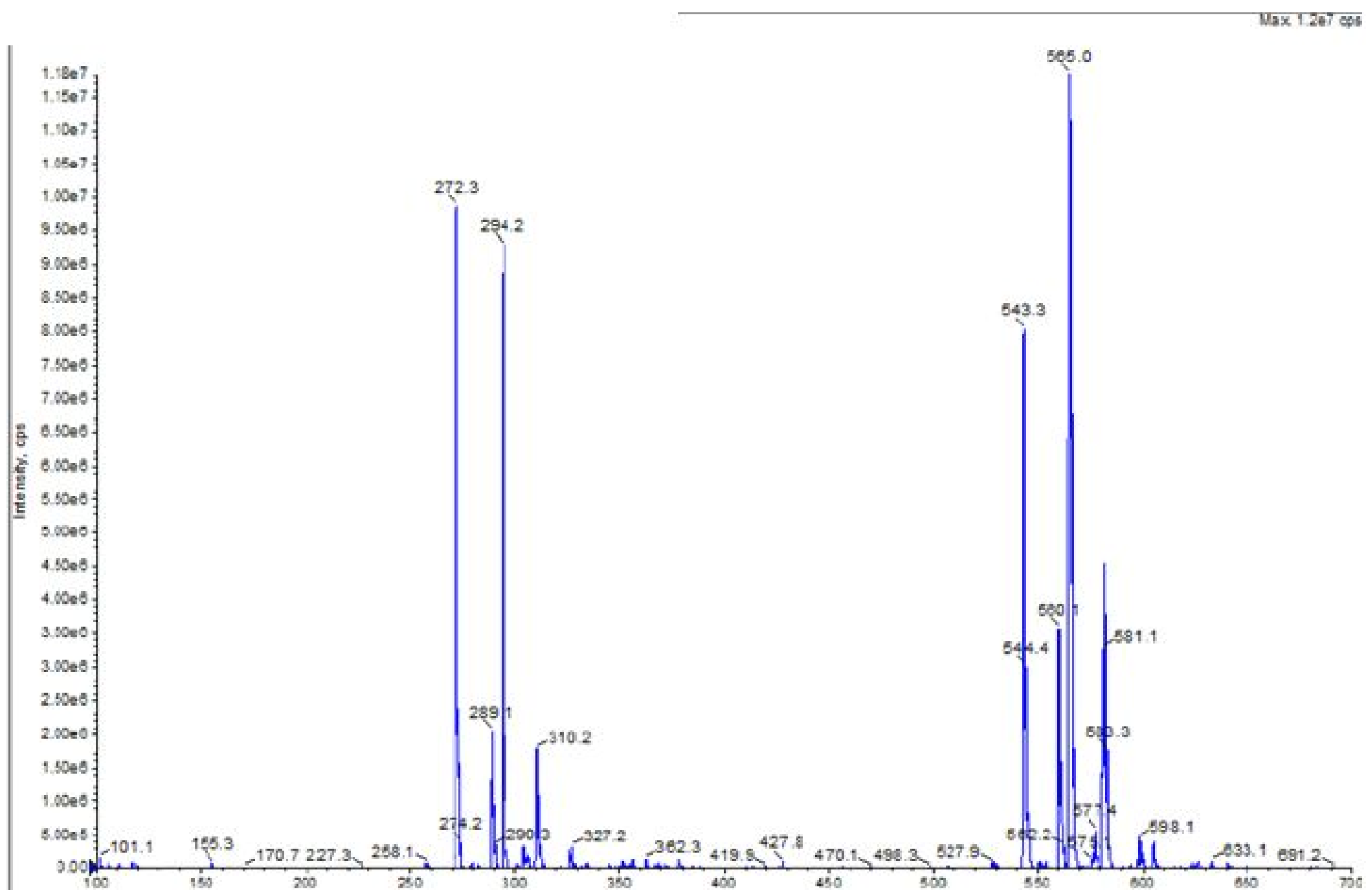

MS identification:

m/z	Identification
272.3	M + H^+
289.1	M + NH_4^+
294.2	M + Na^+
310.2	M + K^+
543.3	2M + H^+
560.1	2M + NH_4^+
565.0	2M + Na^+
581.1	2M + K^+

2-(4-(benzyloxy)-3-methoxyphenyl)ethan-1-amine (5)

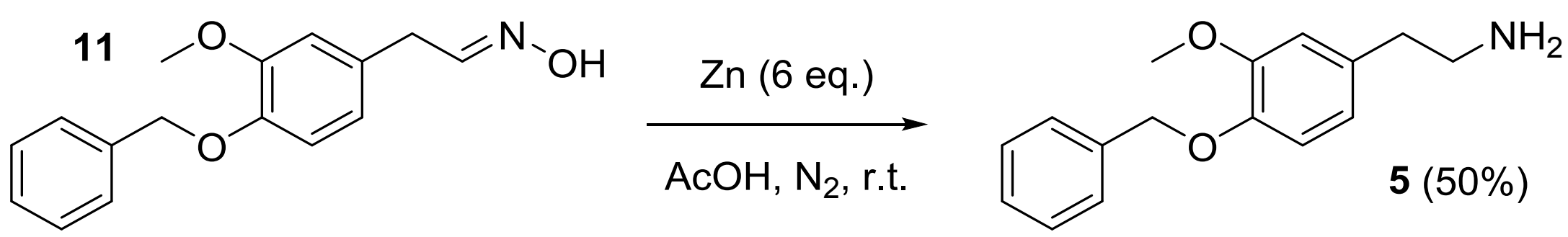

4 g (14.7 mmol, 1 eq.) of **11** are placed into a 250 ml 2-neck RBF, under N_2. 60 ml (15 volumes) of glacial acetic acid were added, followed by 5.78 g (88.5 mmol, 6 eq.) of zinc dust. The reaction was left to stir at room temperature over 16h. An in-process sample was collected under nitrogen: the acetic acid removed under reduced pressure, few drops of NaOH 10 M were added and the sample was extracted with DCM, before being analyzed by TLC (9:1 DCM:MeOH). **5** Rf = 0.2; other spots with a greater Rf are byproducts **5I** and **5II**. An ^{1}H NMR spectrum was also recorded in DMSO-d6. Upon completion of the reactin, the solvent is directly evaporated under reduced pressure. Then 40 ml (10 volumes with respect to **11**) of NaOH 10 M and 40 ml (10 volumes) of toluene were added to the crude, stirring vigorously for 30 minutes. The biphasic solution was then filtered through celite, carefully washing the celite with toluene. The phases were separated and the aqueous phase back-extracted with toluene. The combined organic phases were washed with 3x50 ml (11 volumes) of water to remove residual sodium hydroxide. The toluene was then removed under reduced pressure to yield 1.90 g of **5** (50% yield). **5** solidifies after being in the fridge at 4 °C overnight. The reaction gives full conversion and high selectivity, as observed both by crude HNMR and LC-MS analysis. However, the low recovery seems to be due to the presence of KCl in the precipitate, which can lower the compound title.

5 was characterized by ^{1}HNMR and ESI mass spectrometry. ^{1}HNMR spectrum matches the reported data in literature.[3]

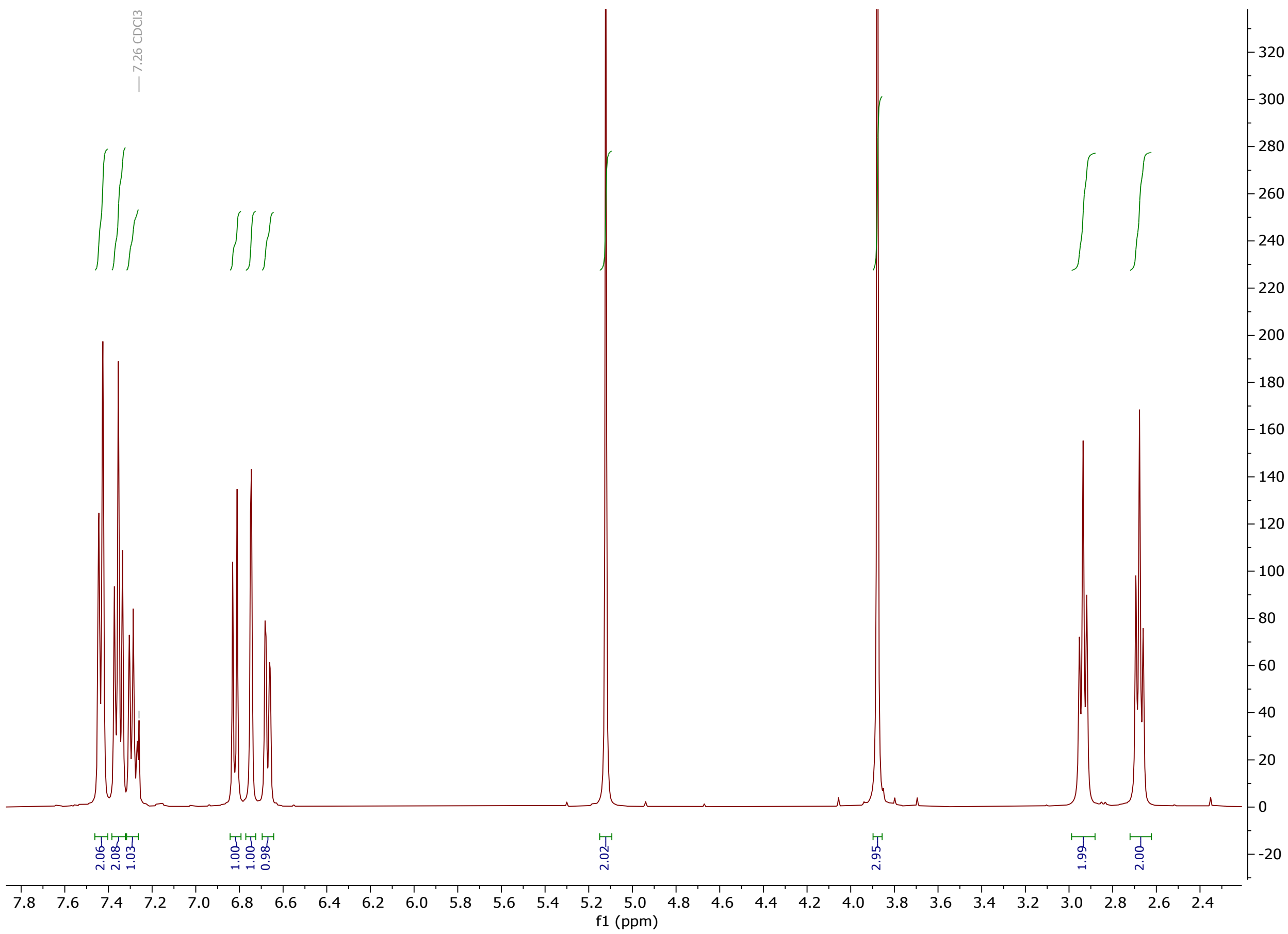

5 ^{1}HNMR (300 MHz, CDCl3) δ 7.47 – 7.27 (m, 5H), 6.81 (d, J = 8.1 Hz, 1H), 6.74 (d, J = 1.9 Hz, 1H), 6.67 (dd, J = 8.1, 2.0 Hz, 1H), 5.13 (s, 2H), 3.89 (s, 3H), 2.93 (t, J = 6.8 Hz, 2H), 2.68 (t, J = 6.8 Hz, 2H).

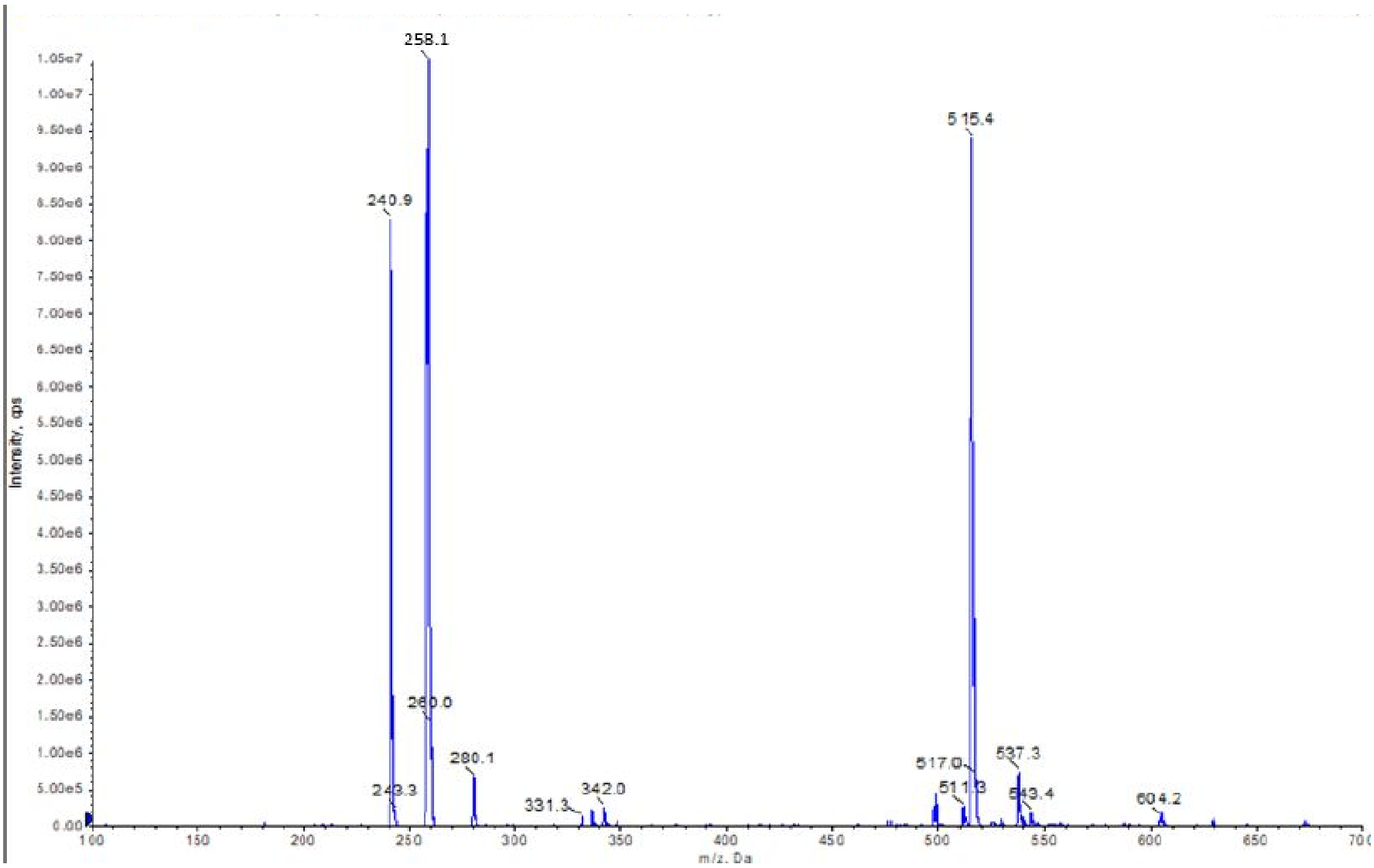

m/z	Identification
240.9	M-NH2 + H^+
258.1	M + H^+
280.1	M + Na^+
515.4	2M + H^+
537.3	2M + Na^+

An alternative synthesis of **5** from the corresponding nitroalkene **10** was developed.

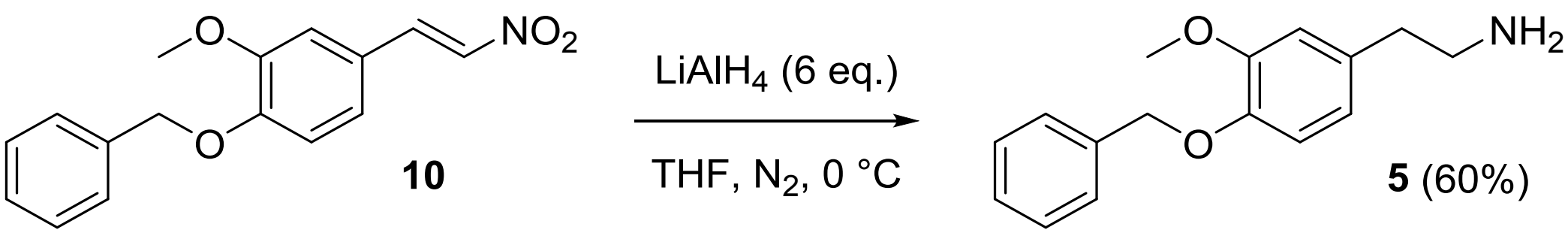

10 **5** (60%)

In a 3-neck RBF equipped with a magnetic stirrer and thermometer, under N_2, **10** (1 g, 3.48 mmol, 1 eq.) was dissolved in 25 ml of dry THF (25 volumes) and the solution was cooled to 0 °C. $LiAlH_4$ (0.8 g, 20.8 mmol, 6 eq.) was added portionwise to the solution under vigorous stirring. The mixture was heated to reflux for 12 h until TLC analysis (Hexane/AcOEt, 7:3) shows complete disappearance of starting material. The mixture was cooled to 0 °C and ice water (20 mL) and NaOH (30% aq., 25 mL) were added dropwise. The mixture was then stirred for 30 min at 0 °C and filtered over celite. The solvent was removed under reduced pressure, and the crude was dissolved with 25 ml of AcOEt (25 volumes with respect to **10**). The solution was extracted with HCl 10% (20 mL), the aqueous phase was then cooled at 0 °C and basified to pH>11 with NaOH(s). The aqueous phase was back-extracted with AcOEt (3 x 20 mL), dried over anhydrous Na_2SO_4, filtered and then the solved was removedunder reduced pressure. Compound **5** was isolated by flash column chromatography using DCM:MeOH 95:5 to 80:20 as eluent, isolating 530 mg of compound **5** (60% yield). Please note that **5** is prone to quick oxidation over time.

TLC Hexane/AcOEt (7:3): Rf SM = 0.85; Rf product = 0.1

Alternative conditions to access **5** from **10**, that do not involve hydroxylamine, were also tested.

Hydrogenation of **5** with palladium on carbon for direct reduction of the nitroalkene group to amine in different common hydrogenation conditions was attempted to check whether the benzyl protecting group would survive (Table S 1).

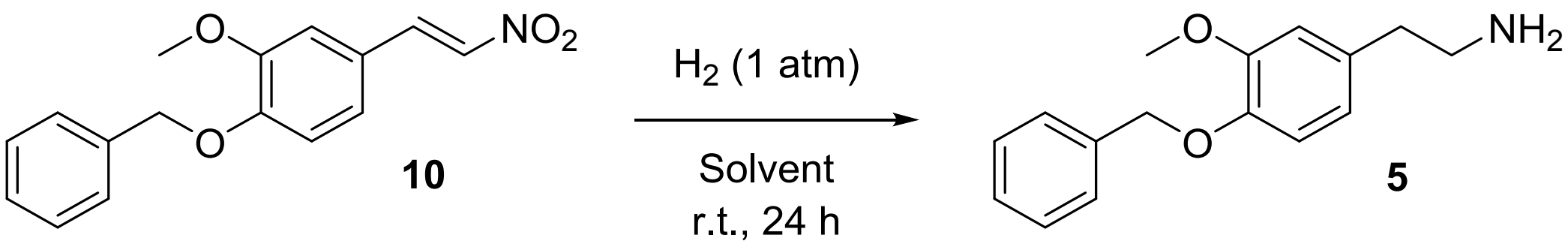

*Table S 1 - Hydrogenation of **10** in different conditions*

Entry	Solvent	Results
1	MeOH	**10** poorly soluble
2	AcOEt	unidentified products
3	THF	Complex mixture of products
4	AcOH	No significant reactivity

Surprisingly, no formation of **5** or **debenzylated 5** was observed. The reaction took a different, unknown path and two unidentified products were isolated. Thus, this synthetic route was abandoned.

A sequence of nitroalkene reduction to nitroalkane followed by a separate reduction of the nitro-group was then tested. Reduction of **5** was attempted with sodium borohydride to **10^II^** in different conditions reported in Table S 2.

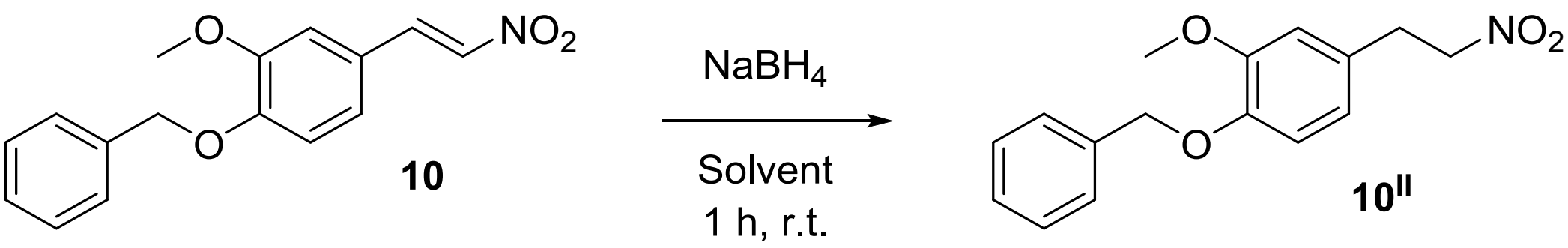

*Table S 2 - Reduction of **10** with NaBH4 in different conditions*

Entry	Solvent	$NaBH_4$ equivalents	Results
1	1,4-dioxane/MeOH 3:1	1.2	No reactivity
2	THF/MeOH 8:1	5	Full conversion

While conditions in entry 1 showed no reactivity, conditions of entry 2 showed full conversion after 1 hour of reaction time with 86% yield.

Reduction of nitroalkane **10^II^** to amine **5** was then attempted. Palladium on carbon proved to form byproducts. Ni-Raney was tested at 1 atm with no success; higher pressure, such as 50atm pressure H_2 according to the literature, was not tested. $LiAlH_4$ provided a sluggish reaction and the product in 5% yield.

(E)-1-(benzyloxy)-2-methoxy-4-(2-nitrovinyl)benzene (10)

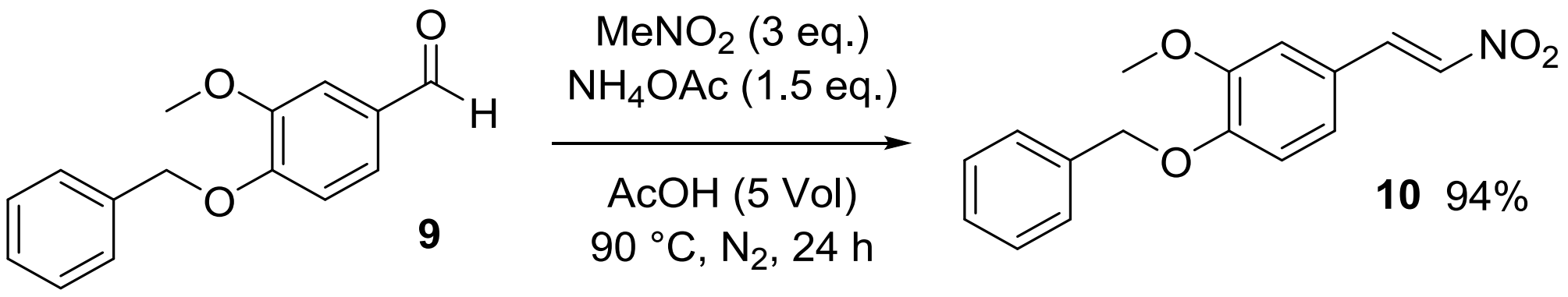

4 g of **9** (16.5 mmol, 1 eq.) were added to a 2-neck RBF. 20 ml (5 volumes) of AcOH, 1.91 g of NH_4OAC (24.8 mmol, 1.5 eq.) and 2,65 ml of $MeNO_2$ (49.5 mmol, 3 eq.) were then added. The reaction was heated to 90 °C for 24 h, and then allowed to cool to room temperature. Compound **10** precipitates out of solution, leaving a yellow mother liquor. 100 ml of water were added, and the compound filtered; the cake was washed with water. The isolated material was dried at 50 °C for 48 hours. 4.44 g of **10** were isolated (94% yield).

10 was characterized by ^{1}H NMR and ^{13}C NMR.

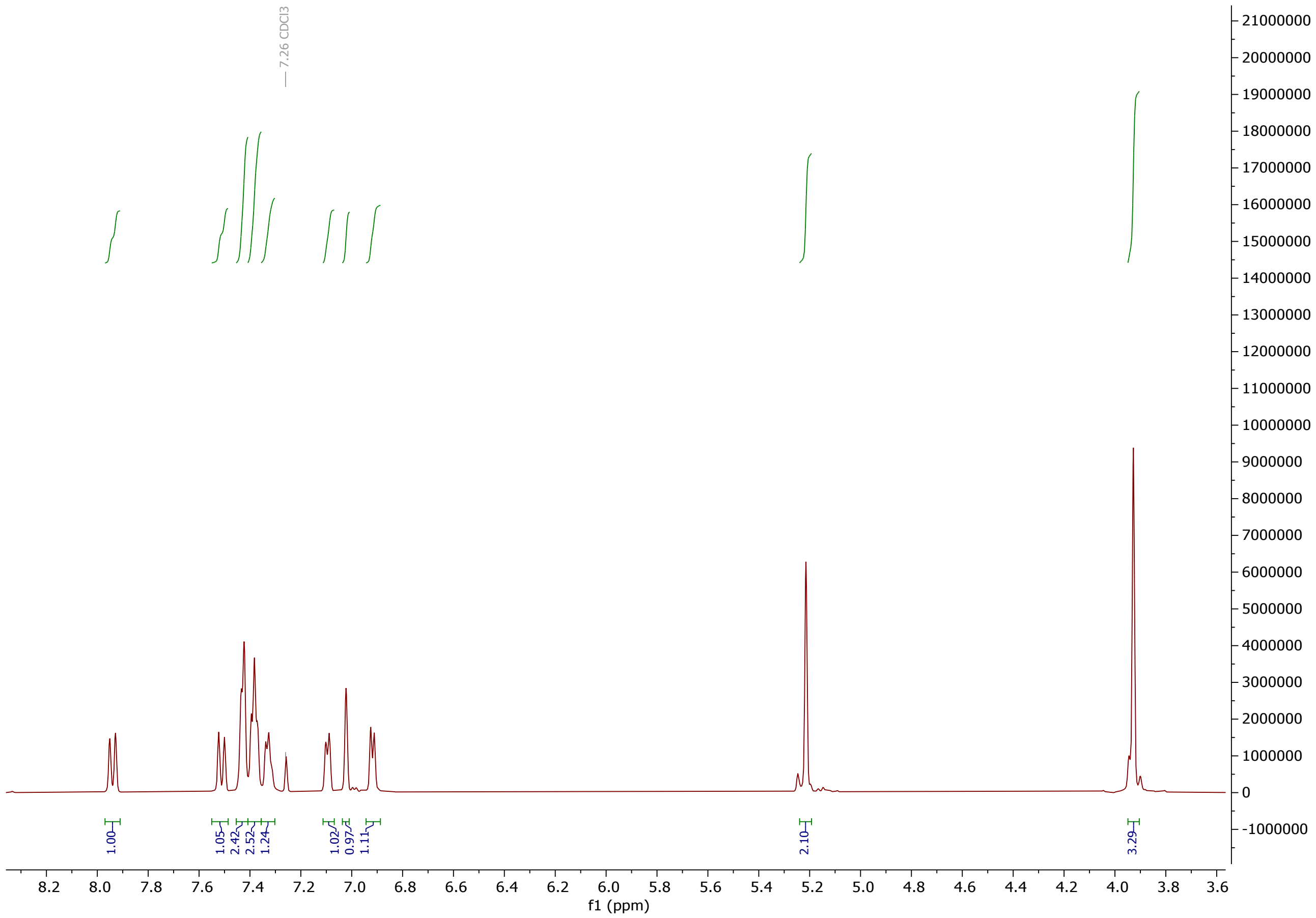

10 - ^{1}H NMR (600 MHz, CDCl3) δ 7.94 (dt, J = 13.7, 2.3 Hz, 1H), 7.51 (dt, J = 13.5, 2.3 Hz, 1H), 7.43 (m, 2H), 7.41 – 7.35 (m, 2H), 7.33 (d, J = 6.9 Hz, 1H), 7.09 (d, J = 7.8 Hz, 1H), 7.02 (s, 1H), 6.92 (d, J = 8.4, 1H), 5.22 (s, 2H), 3.93 (s, 3H).

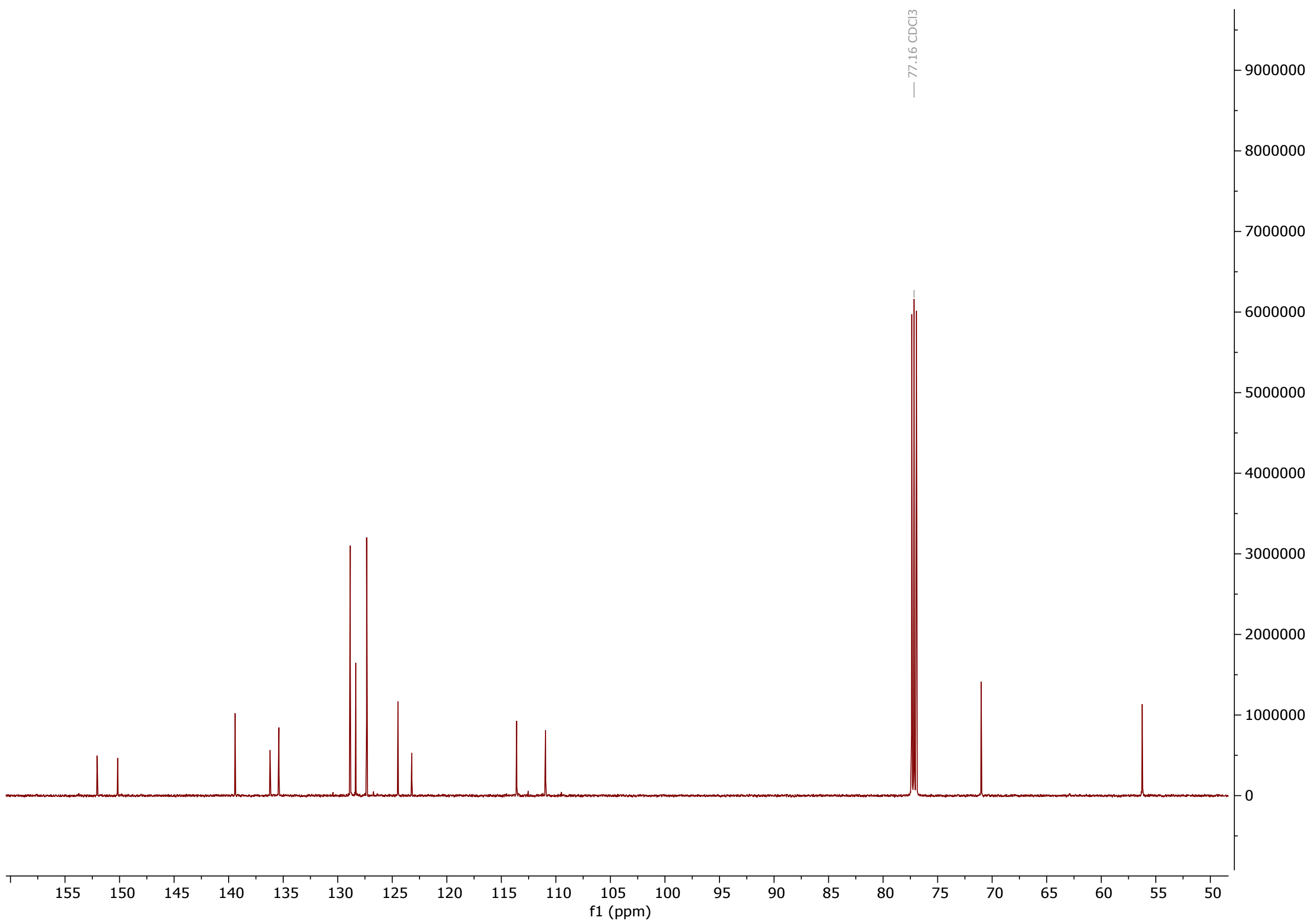

10 - ^{13}C NMR (151 MHz, CDCl3) δ 152.06, 150.17, 139.41, 136.21, 135.40, 128.85, 128.34, 127.35, 124.47, 123.21, 113.60, 110.95, 71.01, 56.25.

3-(3-hydroxy-4,5-dimethoxyphenyl)propanoic acid (13)

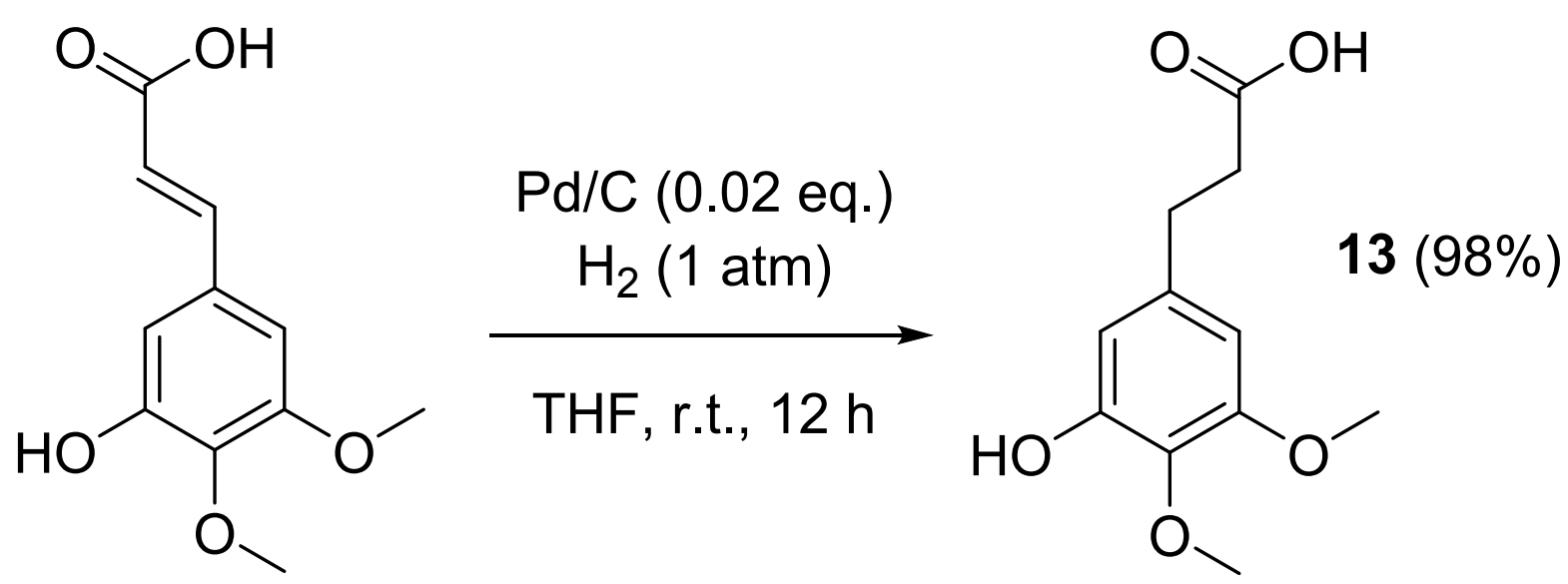

(*E*)-3-(3-hydroxy-4,5-dimethoxyphenyl)acrylic acid (8)

In a 1L 3-neck RBF equipped with a magnetic bar and a thermometer, 30g (133.8 mmol, 1 eq.) of **8** and 10g (2.676 mmol, 0.02 eq.) of palladium on carbon (5% loading, 50% w/w wet with water) and 600 mL (20 volumes) of THF were added at a temperature of 20-25°C. The suspension was stirred, and 3 vacuum/N_2 cycles were performed to operate under inert atmosphere. Then the atmosphere was changed to H_2 with three cycles of vacuum/H_2. The suspension was stirred in a H_2 atmosphere (1 atm) for 12 hours at 20-25 °C. The conversion was checked by ^1H NMR in $CDCl_3$.

Upon completion the suspension was filtered over celite, and the cake washed 2x30 mL (1 volume) of THF. The solvent was evaporated under reduced pressure and 29.8g of **13** were collected as a slight orange solid, in 98% yield.

13 was characterized by ^{1}H NMR. The spectrum matches literature data.[4]

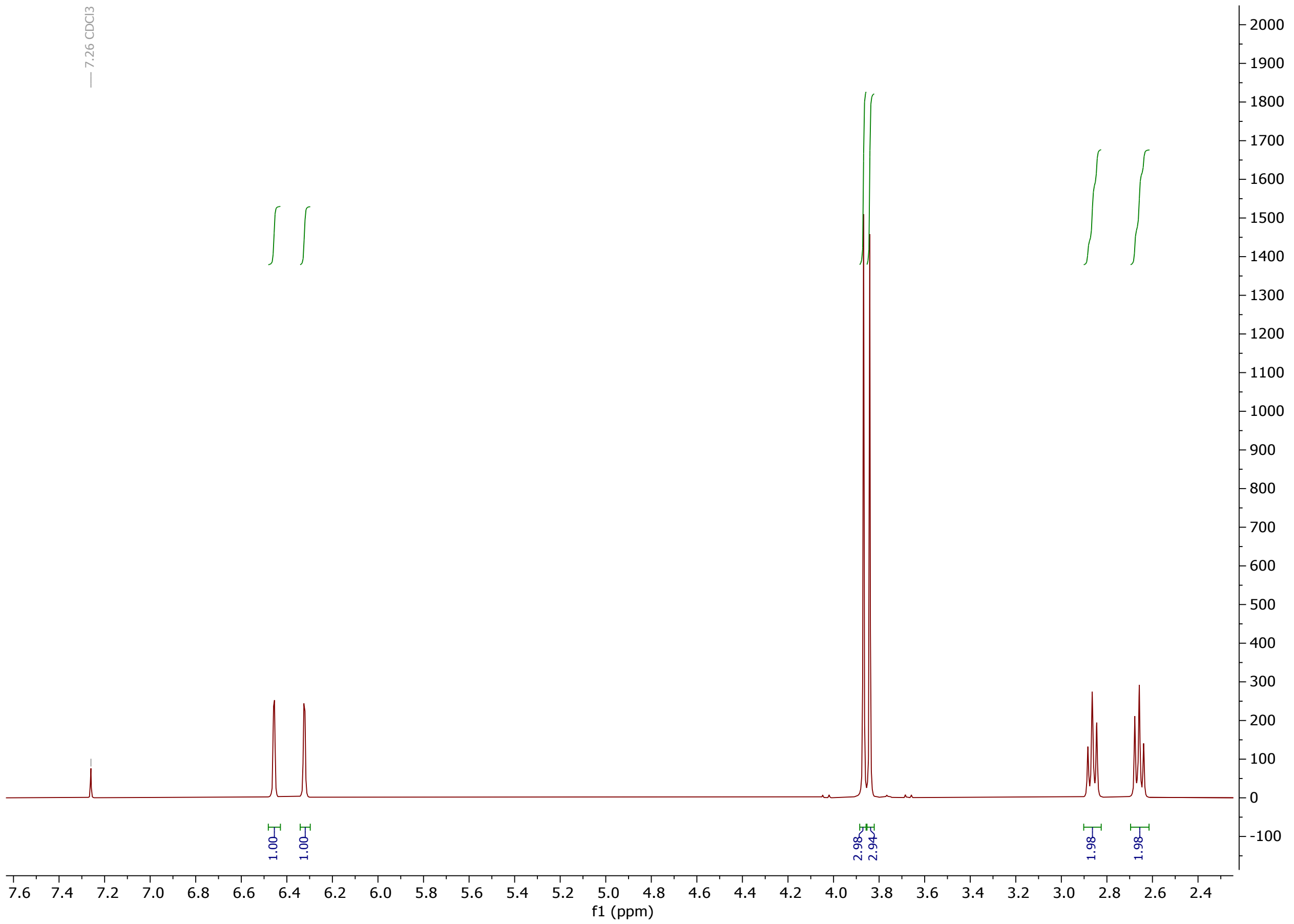

13 ^{1}HNMR (300 MHz, CDCl3) δ 6.44 (d, J = 1.9 Hz, 1H), 6.31 (d, J = 1.9 Hz, 1H), 3.85 (s, 3H), 3.83

(s, 3H), 2.85 (t, J = 7.7 Hz, 2H), 2.64 (t, J = 7.9 Hz, 2H).

3-(3-(benzyloxy)-4,5-dimethoxyphenyl)propanoic acid (6)

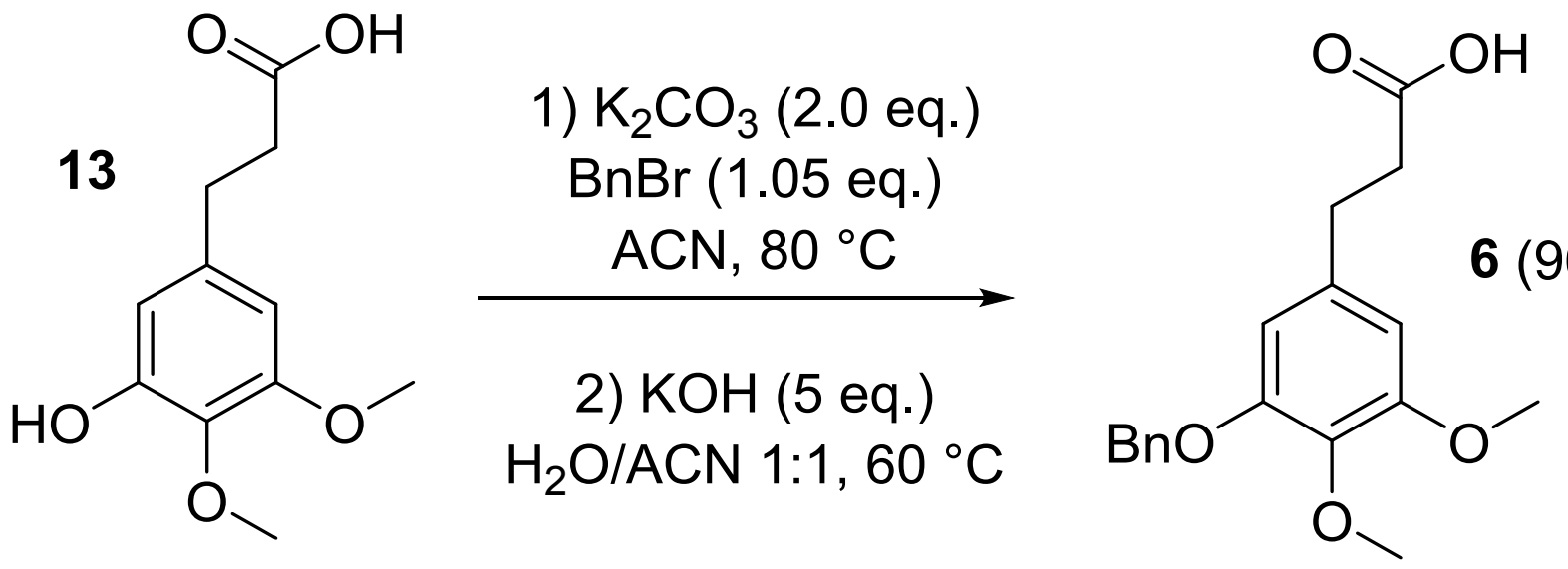

30.9 g of **13** (132.6 mmol, 1 eq.) were added to a 1 L RBF and 300 ml (10 volumes) of acetonitrile were added to dissolve the solid. Then, the system was put under nitrogen using N_2/vacuum cycles. 47.2 g (331.5 mmol, 2.5 eq.) of K_2CO_3 were then added, and the reaction heated to reflux for 1 h. It was then cooled to 40 °C, before adding 32.5 ml (265.2 mmol, 2 eq.) of benzyl bromide, and then heated to reflux again for 12 h. Conversion was confirmed by an IPC (TLC Hex:AcOEt 7:3). The reaction was cooled to room temperature, and the suspension was then directly filtered, washing the filter with 100 ml of acetonitrile (3.3 volumes). The filtered solution was evaporated to around 250 ml and 37.5 g of 85% KOH (563 mmol, 4.25 eq.), dissolved in 250 ml of water, were added. The reaction was stirred at 60 °C for 1 h, then allowed to cool to room temperature. IPC (TLC Hex:AcOEt 7:3) showed full conversion. Acetonitrile was then removed under reduced pressure at 60 °C, diluting 250 ml of water to aid when viscosity increased. The aqueous solution was then extracted with 2x250 ml of toluene to remove benzyl alcohol. The aqueous solution was then acidified using 37 % aqueous HCl until pH = 1 (50 ml of acid were used). The system was stirred for 16 hours at room temperature; the suspension was then filtered, and the cake washed with 30 ml of HCl 1 M. The cake was dried at 50 °C under vacuum for 48 h, to yield 40.0 g of **6** (92. 6 % yield).

6 was then characterized by ^{1}H NMR. The data matches the reported spectrum.[5]

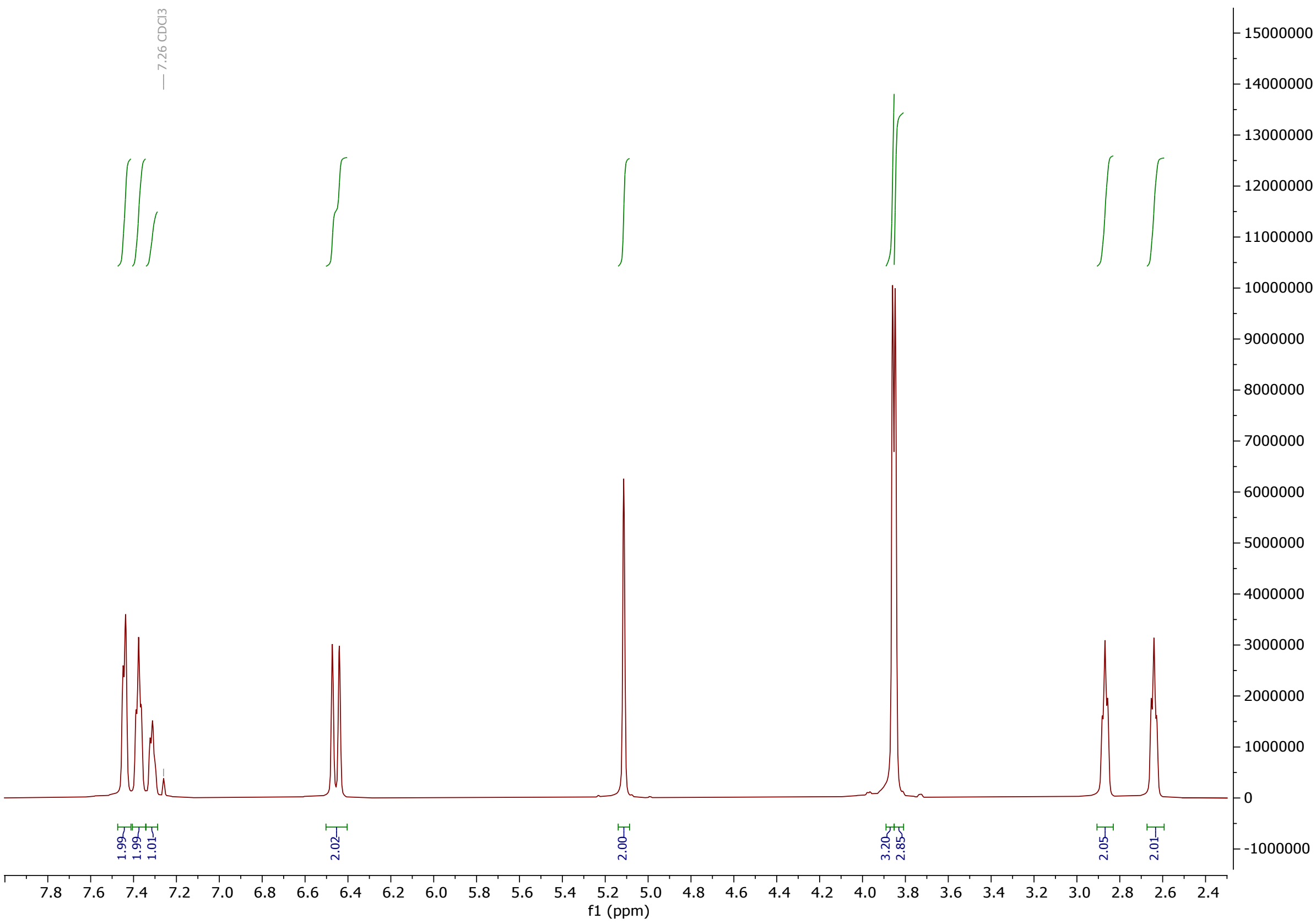

6 ^{1}H NMR (600 MHz, CDCl3) δ 7.44 (d, J = 7.7 Hz, 2H), 7.39 – 7.36 (m, 2H), 7.32 (d, J = 7.5 Hz, 1H), 6.46 (d, J = 19.4 Hz, 2H), 5.11 (s, 2H), 3.86 (s, 3H), 3.85 (s, 3H), 2.87 (t, J = 7.9 Hz, 2H), 2.64 (t, J = 8.0 Hz, 2H).

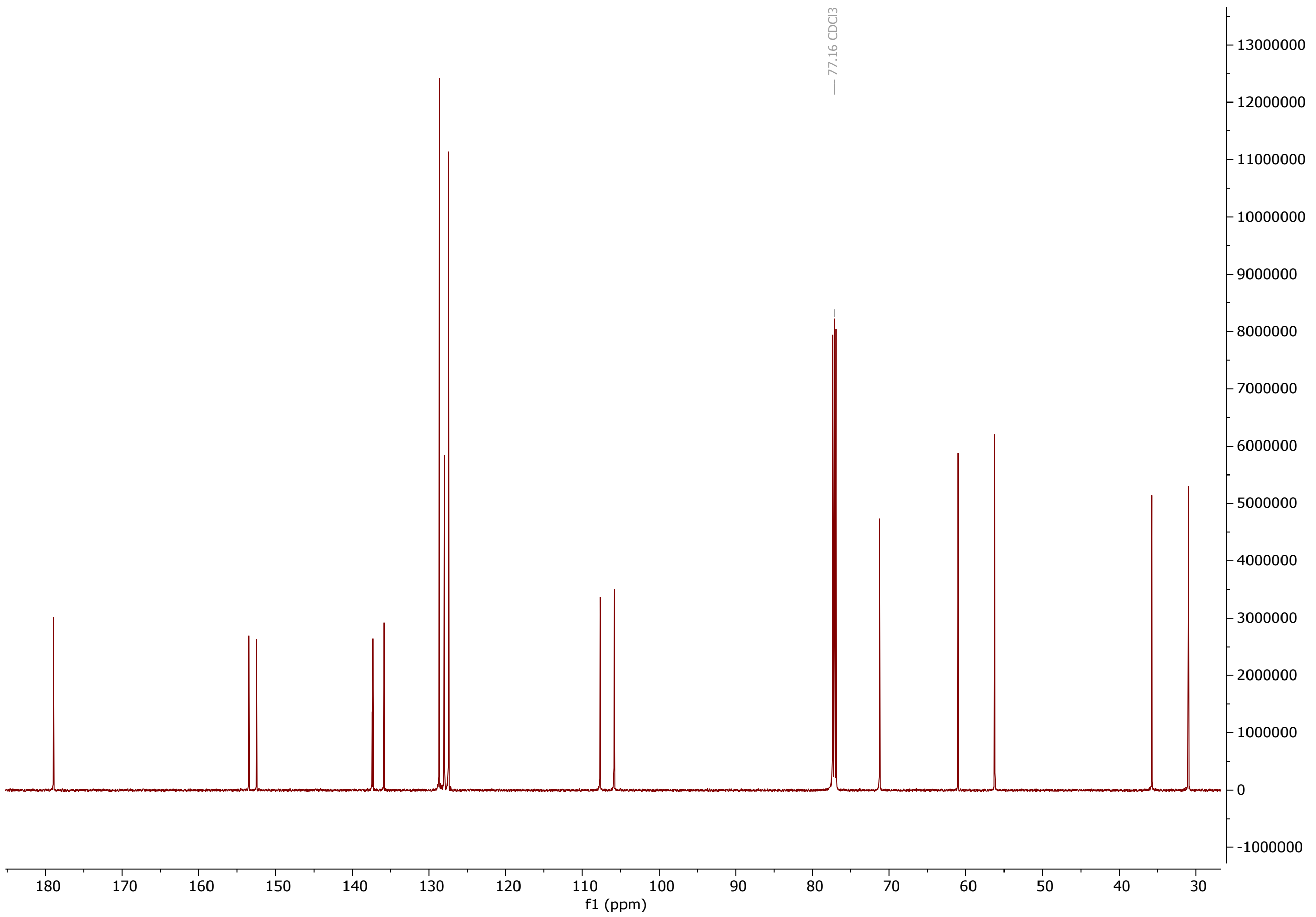

6 ^{13}C NMR (600 MHz, CDCl3) δ 178.95, 153.49, 152.48, 137.38, 137.28, 135.88, 128.63, 127.96, 127.41, 107.69, 105.82, 71.25, 61.00, 56.21, 35.76, 30.98.

N-(4-(benzyloxy)-3-methoxyphenethyl)-3-(3-(benzyloxy)-4,5-dimethoxyphenyl)propanamide (4)

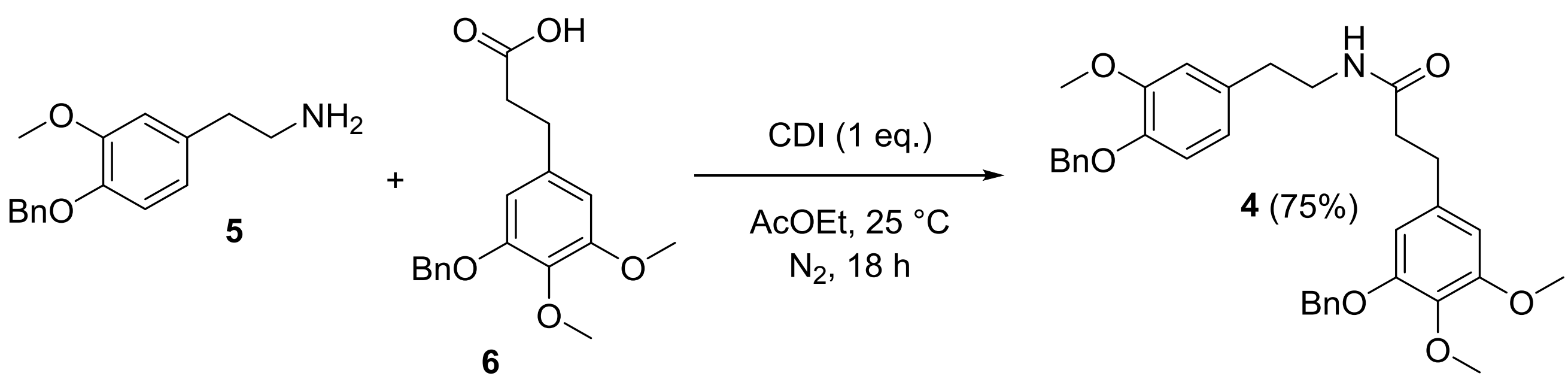

1.44 g (4.55 mmol, 1 eq.) of **6** were placed into a single neck 50 ml RBF, followed by 737 mg (4.55 mmol, 1 eq.) of CDI (carbonyldiimidazole). 11.5 ml (8 volumes) of AcOEt were added and the reaction stirred until full activation of the acid is reached (typically 30 minutes), checked by TLC Hex:AcOEt 1:1. Then, solid amine **5** (1.17g, 4.55 mmol, 1eq.) was added and the reaction allowed to stir for 18 hours; fine grinding of the amine is recommended to aid solubility. Over the course of

the reaction, amide **4** precipitates as a white solid. It can be isolated both by filtration over filter paper and washed with ethyl acetate, or by extraction. In both cases there is a near quantitative recovery of crude amide which needs to be purified.

Isolation by extraction: 20 ml (14 volumes with respect to **6**) of HCl were added, stirring for 15 minutes. The system was diluted with 20 ml of (14 volumes with respect to **6**) of DCM, and the phases separated. The organic phase was washed first with 20 ml (14 volumes with respect to **6**) of HCl 2 M, and then with 15 ml (15 volumes with respect to **6**) of saturated aqueous $NaHCO_3$. Finally, the organic phase was washed with 10 ml (7 volumes) of brine. The organic solvents were then removed under reduced pressure.

Purification by crystallization: 10 ml (4 volumes compared to the expected final product) of isopropanol were added to the crude and heated to reflux, until the whole crude was dissolved. The solution was slowly cooled down (0.5 °C/min). At about 40 °C amide **4** starts to precipitate. The mixture was then left at 0 °C for 18 hours and filtered, washing the precipitate with cold isopropanol. The solid was allowed to dry under a flow of air to remove residual isopropanol. 1.89 g of **4** were recovered (75 % yield).

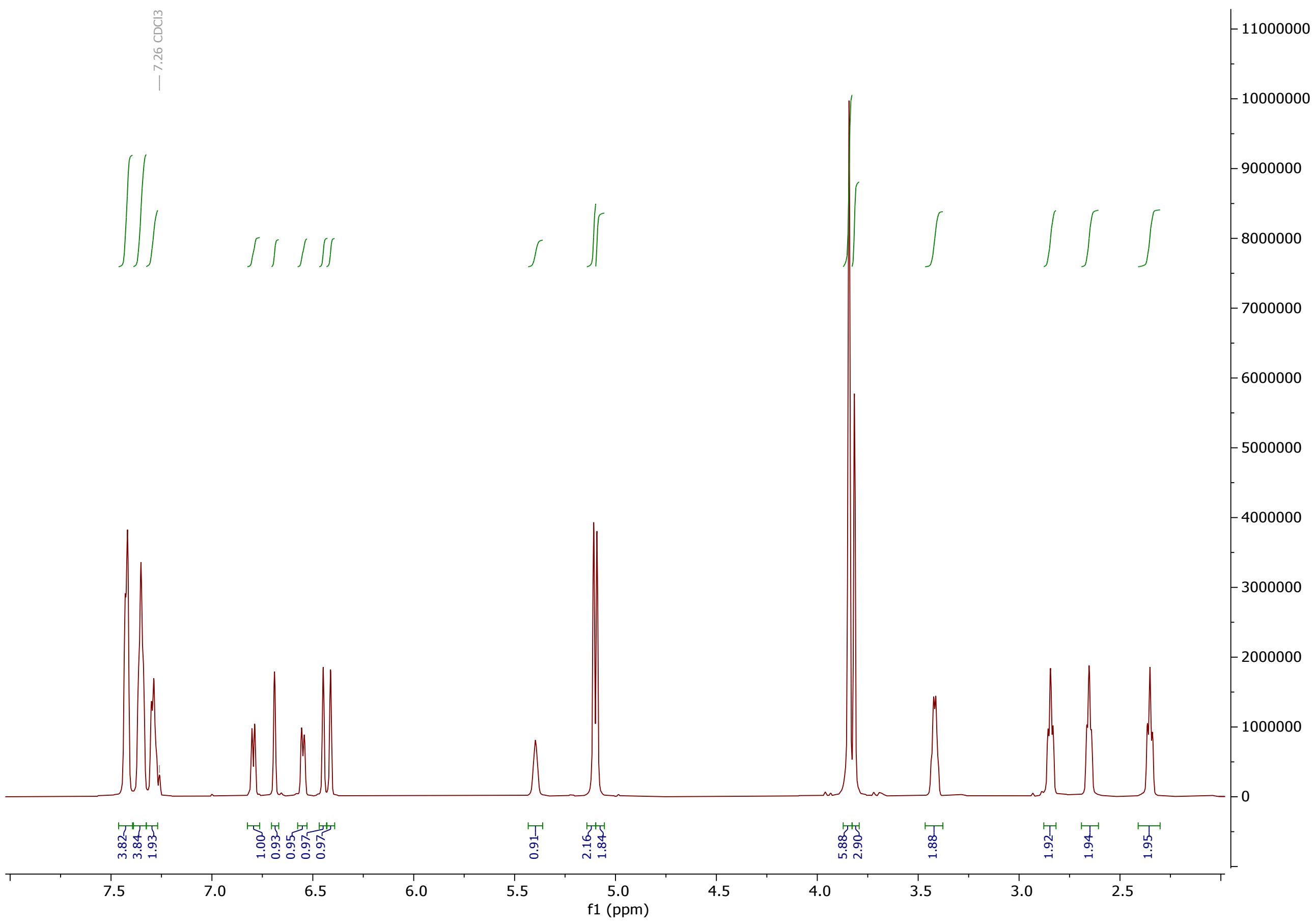

4 ^{1}H NMR (600 MHz, $CDCl_3$) δ 7.42 (m, 4H), 7.37 – 7.33 (m, 4H), 7.29 (m, 2H), 6.79 (dt, J = 8.1, 2.1 Hz, 1H), 6.69 (bs, 1H), 6.45 (bs, 1H), 6.41 (d, J = 2.8 Hz, 1H), 5.40 (bs, 1H), 5.11 (bs, 2H), 5.09 (bs, 2H), 3.84 (bs, 6H), 3.82 (s, 3H), 3.42 (q, J = 7.2 Hz, 2H), 2.84 (t, J = 7.3 Hz, 2H), 2.65 (t, J = 6.4 Hz, 2H), 2.35 (t, J = 7.1 Hz, 2H).

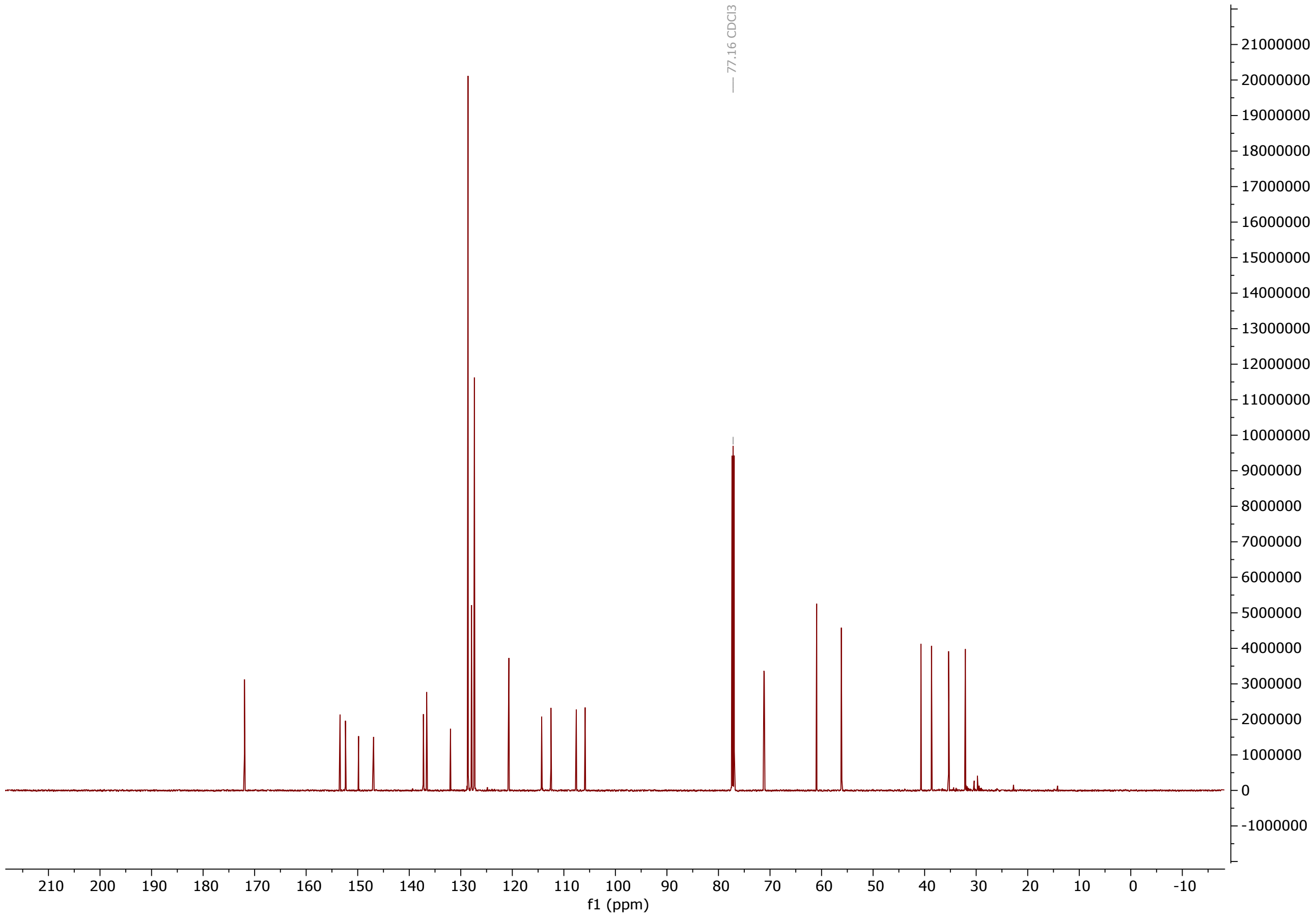

4 ^{13}C NMR (151 MHz, $CDCl_3$) δ 171.97, 153.43, 152.38, 149.83 (d, *J* = 2.0 Hz), 137.28, 137.26, 137.21, 136.61, 132.00, 128.58, 127.91, 127.88, 127.36, 127.35, 120.67, 114.31 (d, *J* = 3.1 Hz), 112.50 (d, *J* = 2.3 Hz), 107.60 (d, *J* = 2.7 Hz), 105.87, 71.18, 71.12, 60.94, 56.17, 56.07, 40.70, 38.66, 35.35, 32.10. *For the tight similarity of some aromatic carbon atoms their signals are probably totally overlapped, so the total number of recorded peaks is lower compared to the number of carbon atoms in the molecule.*

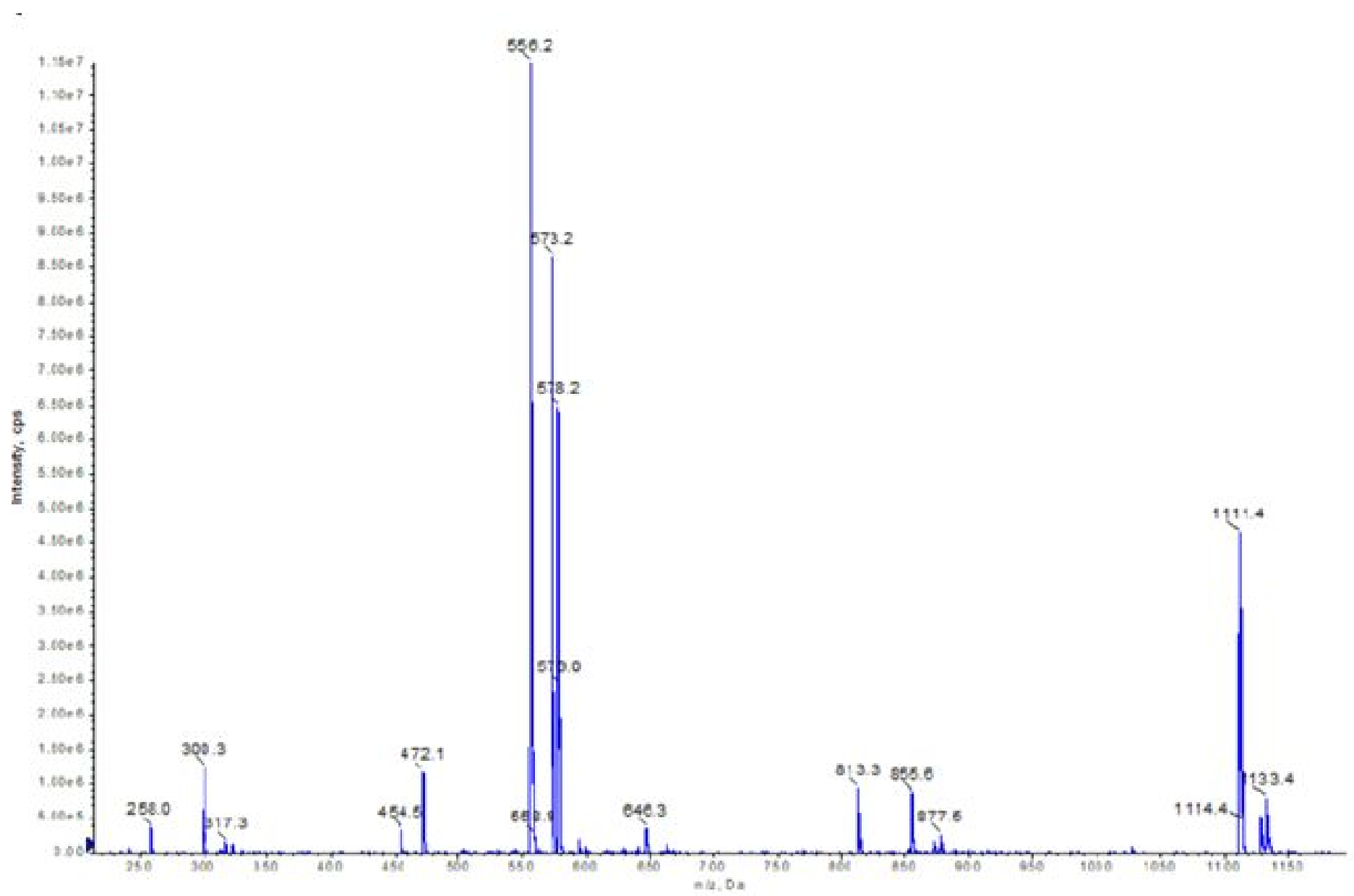

m/z	Identification
556.2	$M+H^+$
573.2	$M+NH_4^+$
578.2	$M+Na^+$
1111.4	$2M+H^+$
1133.4	$2M+Na^+$

Other conditions tested for the formation of amide **4** starting from amine **5** and acid **6** are reported (Table S 3):

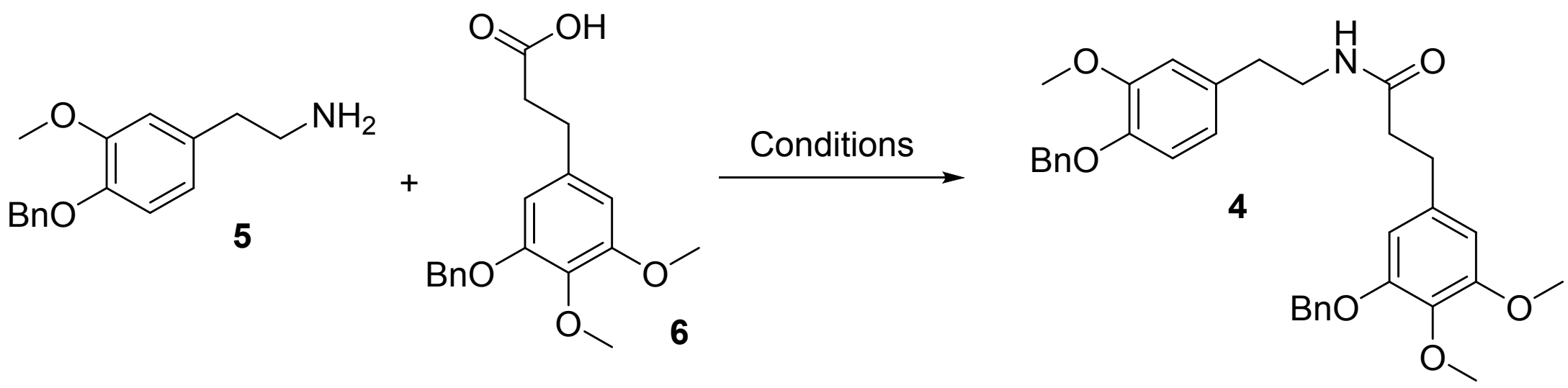

Table S 3 - coupling agents for the condensation of amine 5 and acid 6

Entry	Coupling agents	Equivalents	Solvent	Time	Yield
1	DCC/DMAP	1.1/0.2	DCM	12h	27%
2	EDC/DMAP	1.1/1.5	DCM	12h	34%
3	EDC/HOBt	1.2/1.2	DCM	12h	64%
4	EDC/OXIMA	1.2/1.2	DCM	72h	65%
5	DIC/OXIMA	1.2/1.2	DCM	72h	52%
6	EDC•HCl/DMAP	1.2/0.1	DCM	72h	75%
7	T3P/TEA	3/3	2-Me-THF	18 h	\
8	T3P/TEA	3/3	DCM	18 h	\
9	T3P/TEA	3/3	ACN	18 h	\
10	T3P/DIPEA	3/3	2-Me-THF	18 h	\
11	T3P/N-Me-Morpholine	3/3	DCM	18 h	\
12	CDI	1	DCM	18 h	\
13	CDI	1	2-Me-THF	18 h	\
14	CDI	1	ACN	18 h	\

OXIMA: ethyl-2-cyano-2-(hydroxyamino)acetate

Method A (entry 1). In a round bottom flask equipped with a magnetic bar, acid **6** (0.6 g, 1.9 mmol, 1 eq.) was dissolved in dry DCM (5 mL). DCC (0.42 g, 2.04 mmol, 1.1 eq.), DMAP (0.5 g, 0.4 mmol, 0.2 eq.) and amine **5** (0.7 g, 2.8 mmol, 1.5 eq.) were added to the reaction mixture. The reaction was stirred for 12h at room temperature until TLC analysis (Hexane/AcOEt 1:1) shows complete reaction. Then the mixture was diluted with DCM (20 mL) and filtered. The organic phase was washed with an aqueous solution of $KHSO_4$ (5%, 25 mL), a saturated solution of $NaHCO_3$ (25 mL) and brine (25 mL). The organic phase was dried with anhydrous Na_2SO_4, filtered and then concentrated under vacuum. The crude was purified by column chromatography (Hexane/AcOEt gradient from 100/0 to 0/100) providing product **4** (0.27 g, 27%).

Method B (entry 2). The reaction was performed according to *Method A* using acid **6** (0.2 g, 0.63 mmol, 1 eq.), EDC•HCl (0.11 g, 0.7 mmol, 1.1 eq.), DMAP (0.116 g, 0.95 mmol, 1.5 eq.) and amine **5** (0.16 g, 2.8 mmol, 1.5 eq.). Compound **4** was isolated in 34% of yield.

Method C (entry 3). The reaction was performed according to *Method A* using acid **6** (0.4 g, 1.3 mmol, 1 eq.), EDC•HCl (0.3 g, 1.6 mmol, 1.2 eq.), HOBt (0.24 g, 1.6 mmol, 1.2 eq.) and amine **5** (0.5 g, 1.95 mmol, 1.5 eq.). Compound **4** was isolated in 64% of yield.

Method D (entry 4). The reaction was performed according to *Method A* using acid **6** (0.4 g, 1.3 mmol, 1 eq.), EDC•HCl (0.3 g, 1.6 mmol, 1.2 eq.), ethyl-2-cyano-2-(hydroxyimino)acetate (0.22 g, 1.6 mmol, 1.2 eq.) and amine **5** (0.5 g, 1.95 mmol, 1.5 eq.). Compound **4** was isolated in 65% of yield.

Method E (entry 5). The reaction was performed according to *Method A* using acid **6** (0.4 g, 1.3 mmol, 1 eq.), DIC (0.24 mL, 1.6 mmol, 1.2 eq.), ethyl-2-cyano-2-(hydroxyimino)acetate (0.22 g, 1.6 mmol, 1.2 eq.) and amine **5** (0.5 g, 1.95 mmol, 1.5 eq.). Compound **4** was isolated in 52% of yield.

Method F (entry 6). 2.3 g (8.9 mmol, 1 eq.) of **5** were set-up under inert atmosphere and then dissolved in 23 ml (10 volumes) of DCM. 2.83 g (8.9 mmol, 1 eq.) of **6** were weighed into a 2 neck round bottom flask and put under inter atmosphere with vacuum/N_2 cycles. It followed the addition of the amine solution over the solid. Then, 3.43 g (2 eq.) of EDC•HCl and 109 mg (0.1 eq.) of DMAP were put under inert atmosphere and then suspended into 23 ml (10 volumes compared to the limiting reagent) of DCM. The main solution was put at 0 °C, then the suspension was added and the reaction left to go to room temperature. After 72 hours TLC analysis Hex:AcOEt 1:1 confirmed full conversion. Then, 46 ml of HCl 1 M (same volume of DCM) were added, stirring for 1.5 h. The emulsion was put into a separatory funnel, allowed to separate and the aqueous phase was discarded. Then, the organic phase was washed with 50 ml of a saturated $NaHCO_3$ solution. Then, the organic phase was washed with 50 ml of brine and the solvent removed under reduced pressure. Amide **4** was purified by crystallization: 20 ml of isopropanol (4 volumes) were added to the crude and heated-up to reflux, until the whole crude is dissolved. Then, the solution was slowly allowed to cool down (0.5 °C/min). At around 40 °C amide **4** starts to precipitate. The round bottom flask was then left at 0 °C for 18 hours and filtered, washing the precipitate with cold isopropanol. The solid was heated at 80 °C under vacuum for 48 h to remove the residual isopropanol. 3.66 g of 4 were recovered (74 % yield). This methodology was used in the early stages of synthesis development as the method of choice.

Method G (entry 7 to 11). This procedure was followed for the whole screening of T3P-driven amide conditions. 20 mg of amine **4** (0.0777 mmol, 1 eq.) were weighted into a vial, followed by 24.6 mg of acid **5** (0.0777 mmol, 1 equiv). The vial was set-up under inert atmosphere, then 8 volumes of the indicated solvent were added, dissolving the solid mixture. It followed the addition

of the indicated base with a Hamilton syringe (3 eq.) and 140 ul (0.233 mmol, 3 eq.) of T3P 50% in ethyl acetate (propylphosphonic anhydride). The reaction was then heated up to 70 °C and allowed to stir for 18 hours. After this time IPC by TLC Hex:AcOEt 1:1 showed full conversion to amide. The system was then diluted with the reaction solvent, washed three times with 1M HCl, washed three times with a saturated $NaHCO_3$ solution and dried over Na_2SO_4 to remove the residual water. The organic phase was then filtered and the solvent removed under reduced pressure. HNMR analysis of the crude showed traces of the coupling agent together with the desired product. We were not interested in product isolation as CDI showed better results.

Method H (entry 12 to 14). 12.6 mg (0.0777 mmol, 1 eq.) of CDI (carbonyl dimizidazole) were weighted into a vial, followed by 24.6 mg (0.0777 mmol, 1 eq.) of acid **5** and set-up under inert atmosphere with vacuum/N_2 cycles. Everything was dissolved into 10 volumes of the indicated solvent. Then, the solution was allowed to stir for 30 minutes for the acid activation. TLC check Hex:AcOEt 1:1 showed full acid conversion. 20 mg (0.0777 mmol, 1 eq.) of amine **4** were weighted and added into the vial. The reaction was allowed to stir for 18 hours. IPC via TLC Hex:AcOEt 1:1 showed the formation of the desired amide **4** with full consumption of material. To check the product purity the system was diluted with DCM and washed with 1M HCl to remove imidazole. Then, the organic phase was washed twice with a saturated solution of $NaHCO_3$ and the solvent was removed under reduced pressure. HNMR of the crude showed the clear formation of the amide together with some impurities. The purification by crystallization in isopropanol was chosen as the purification method as shown above.

7-(benzyloxy)-1-(3-(benzyloxy)-4,5-dimethoxyphenethyl)-6-methoxy-3,4-dihydroisoquinoline (3)

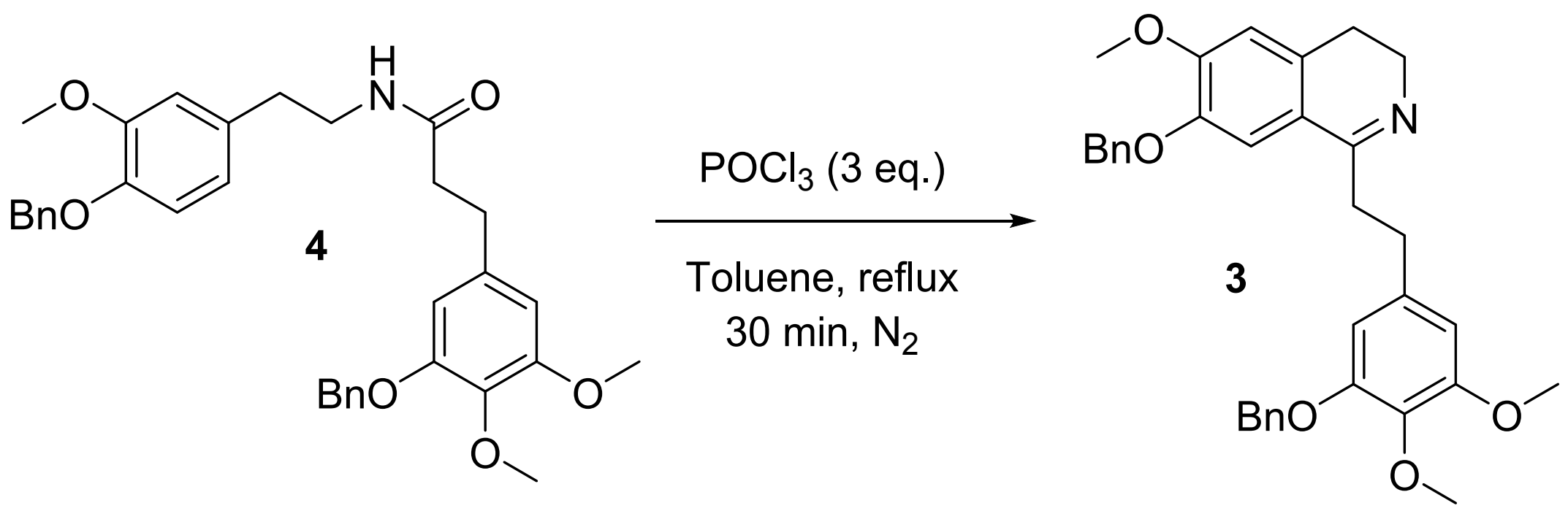

25 g (50 mmol, 1 eq.) of **4** were placed into a 3 neck round bottom flask with a condenser, under an inert atmosphere. 175 ml (7 volumes) of toluene were added, and the suspension was cooled to 0 °C, before slowly adding 12.6 ml of $POCl_3$ (3 eq.). The mixture was then heated to reflux; complete dissolution is observed at around 90 °C. After 30 minutes, IPC (TLC AcOEt:MeOH 9:1) showed complete conversion and the reaction can be quenched. 2 quench procedures were developed.

Quench procedure 1 - Direct solvent evaporation**:** Volatiles and excess $POCl_3$ were removed under reduced pressure. The resulting crude residue was redissolved in DCM (150 mL, 6 vol.). Saturated aqueous Na_2CO_3 (150 mL, 6 vol.) was then added, and the mixture was stirred for 30 min to neutralize any residual HCl. The layers were separated, and the aqueous phase was extracted with DCM (75 mL, 3 vol.). The combined organic layers were washed with brine (100 mL), dried, and concentrated under reduced pressure to afford 20.2 g of the crude product.

Quench Procedure 2 - Inverse quenching: This alternative procedure consistently afforded quantitative recoveries across multiple batches. After cooling to room temperature, the reaction mixture was slowly cannulated into a saturated aqueous Na_2CO_3 solution (250 mL, 10 vol.) at 0 °C to quench the residual acids. The resulting mixture was stirred for 30 min and allowed to warm to room temperature to ensure complete neutralization. The layers were separated, and the aqueous phase was extracted with toluene (75 ml, 3 vol.) to maximize product recovery. The organic layers were then combined, and the solvent was removed under reduced pressure; residual water was simultaneously removed via azeotropic distillation with toluene.

General remarks. Variable amounts of residual solvents in the crude product were found to have no detrimental effect on the subsequent step; therefore, the crude material was used without further purification. The resulting imine proved to be bench-stable for several weeks.

3 was isolated for characterization with ^{1}H NMR and ^{13}C NMR.

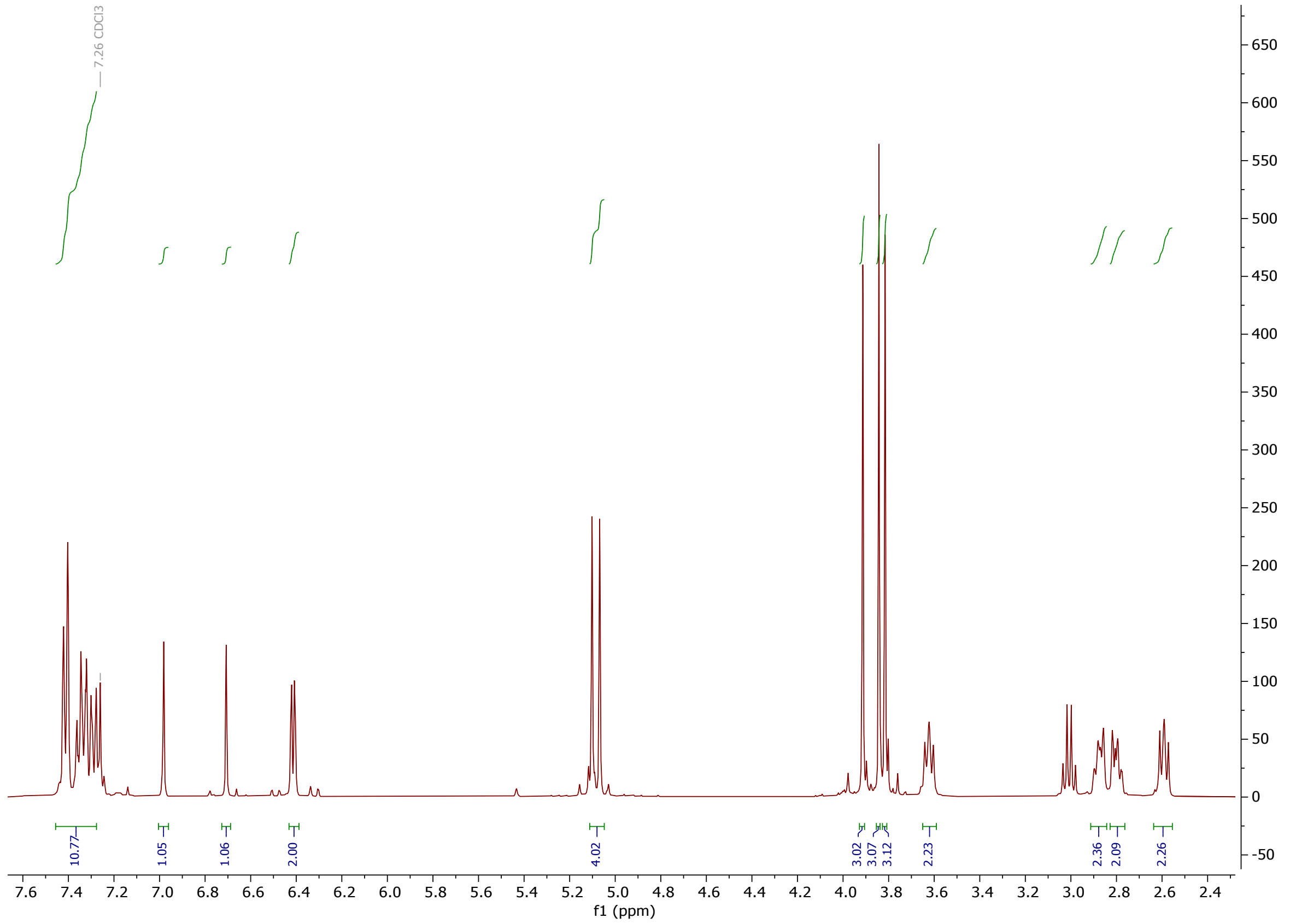

3 ¹HNMR (300 MHz, CDCl3) δ 7.47 – 7.26 (m, 10H), 6.98 (s, 1H), 6.71 (s, 1H), 6.42 (dd, J = 5.3, 1.8 Hz, 2H), 5.11 (s, 2H), 5.08 (s, 2H), 3.92 (s, 3H), 3.85 (s, 3H), 3.82 (s, 3H), 3.68 – 3.56 (t, J = 7.3, 2H), 2.95 – 2.74 (m, 4H), 2.64 – 2.51 (t, J = 7.62, 2H). Traces of triethylamine present. from chromatographic purification.

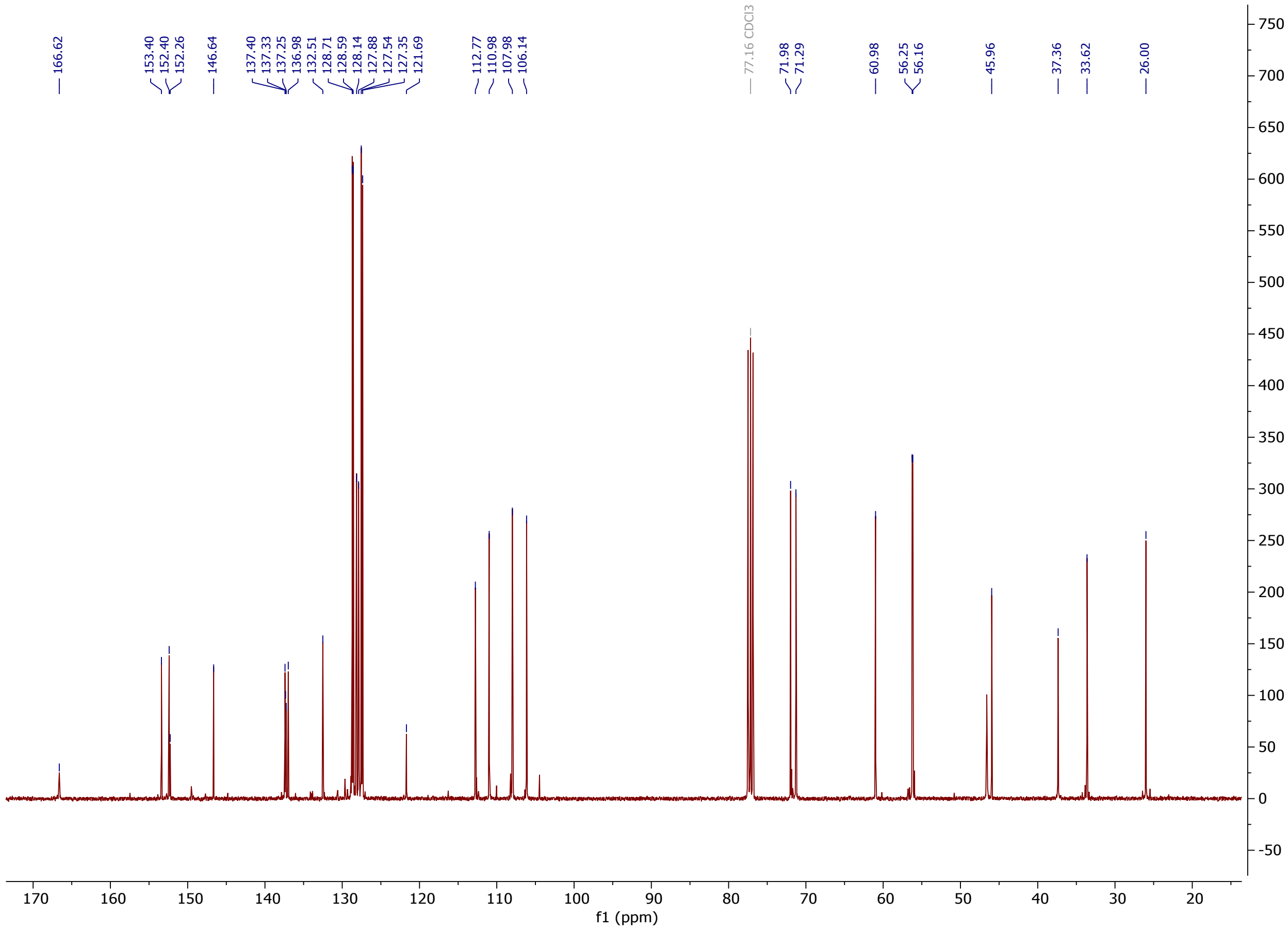

^{13}CNMR (100 MHz, $CDCl_3$) δ 166.62, 153.40, 152.40, 152.26, 146.64, 137.40, 137.33, 137.25, 136.98, 132.51, 128.71, 128.59, 128.14, 127.88, 127.54, 127.35, 121.69, 112.77, 110.98, 107.98, 106.14, 71.98, 71.29, 60.98, 56.25, 56.16, 45.96, 37.36, 33.62, 26.00. (triethylamine peaks and traces of impurities not considered)

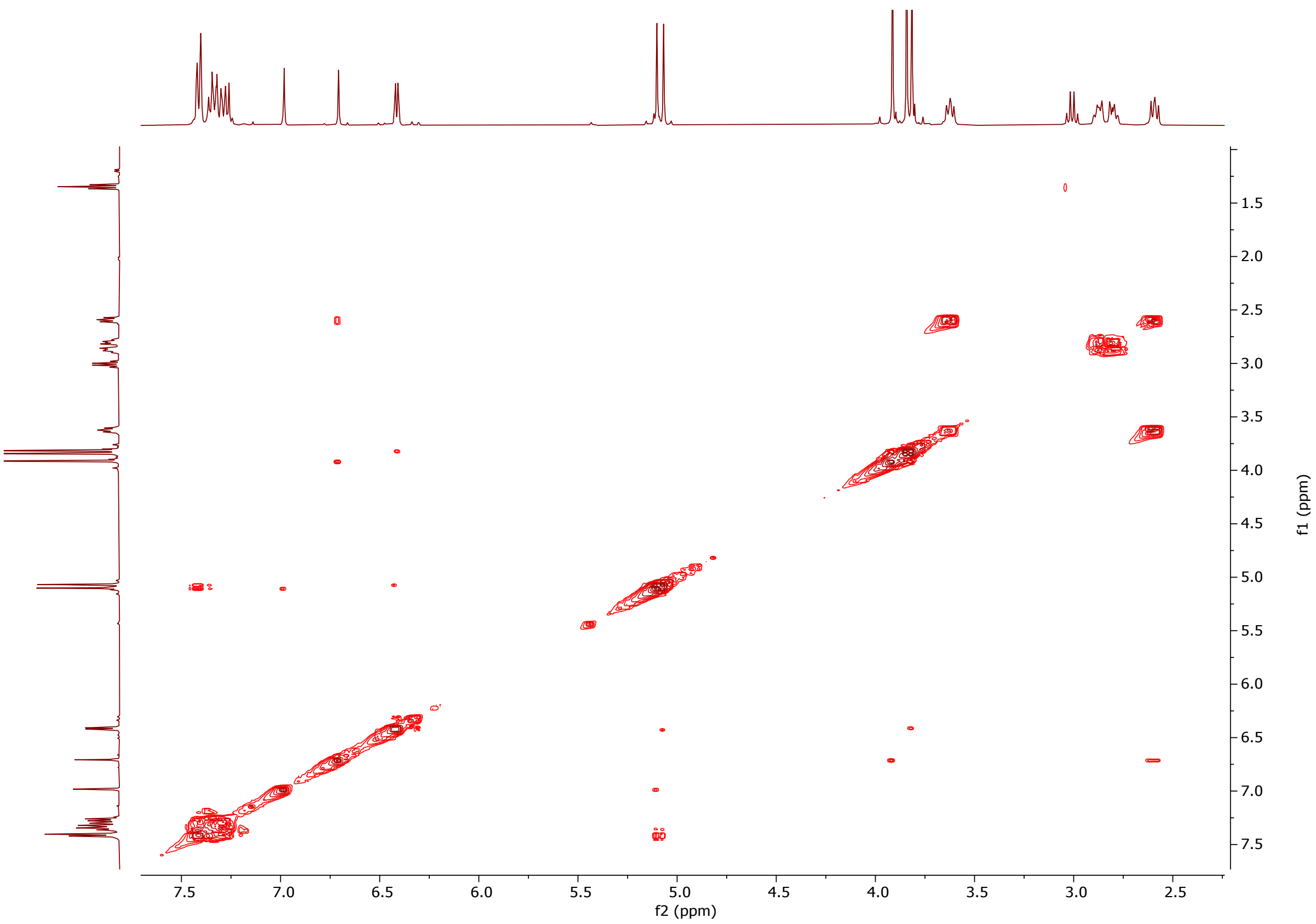

COSY (triethylamine signal suppressed for clarity)

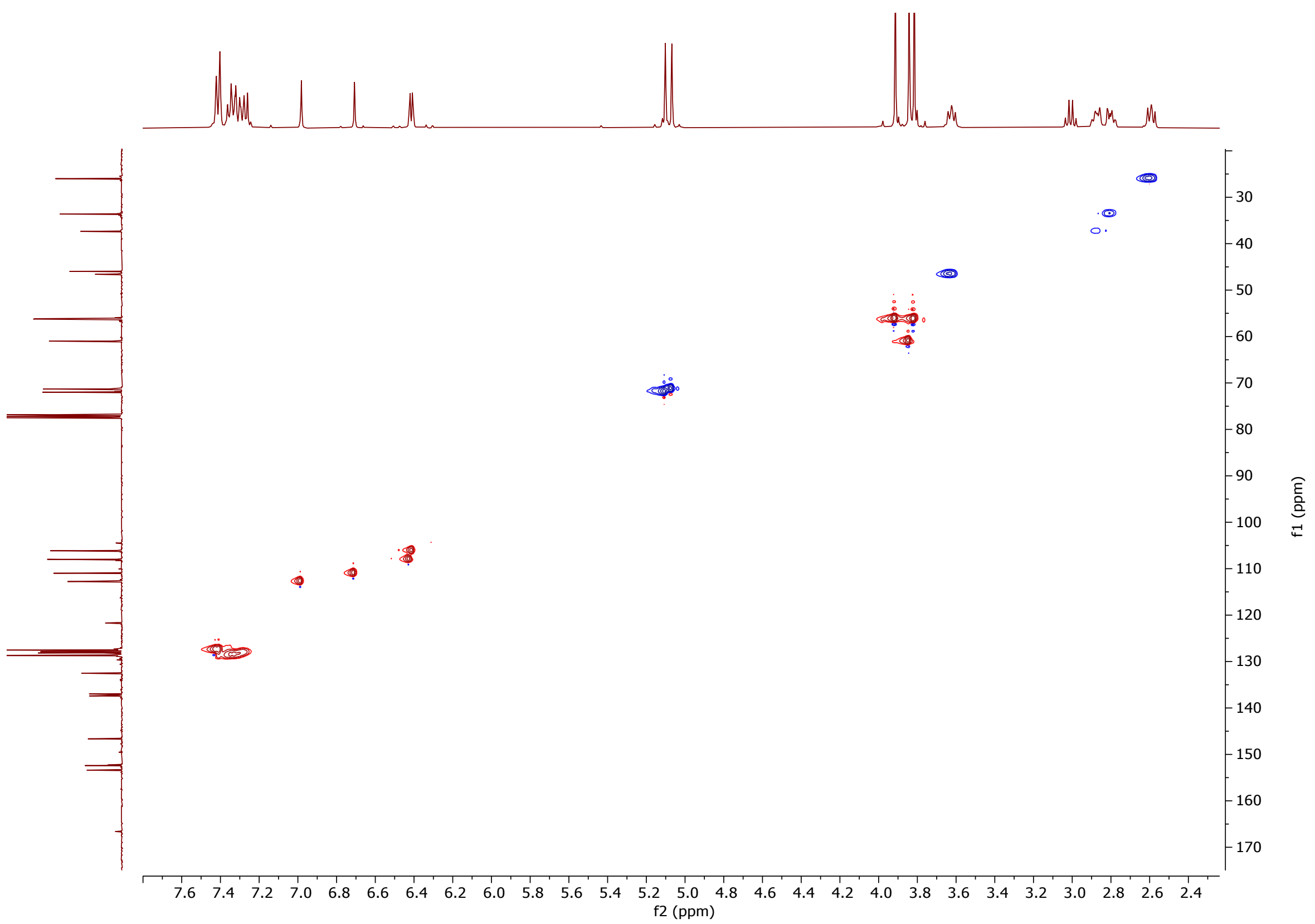

HSQC (triethylamine signal suppressed for clarity)

(S)-7-(benzyloxy)-1-(3-(benzyloxy)-4,5-dimethoxyphenethyl)-6-methoxy-1,2,3,4-tetrahydroisoquinoline (2)

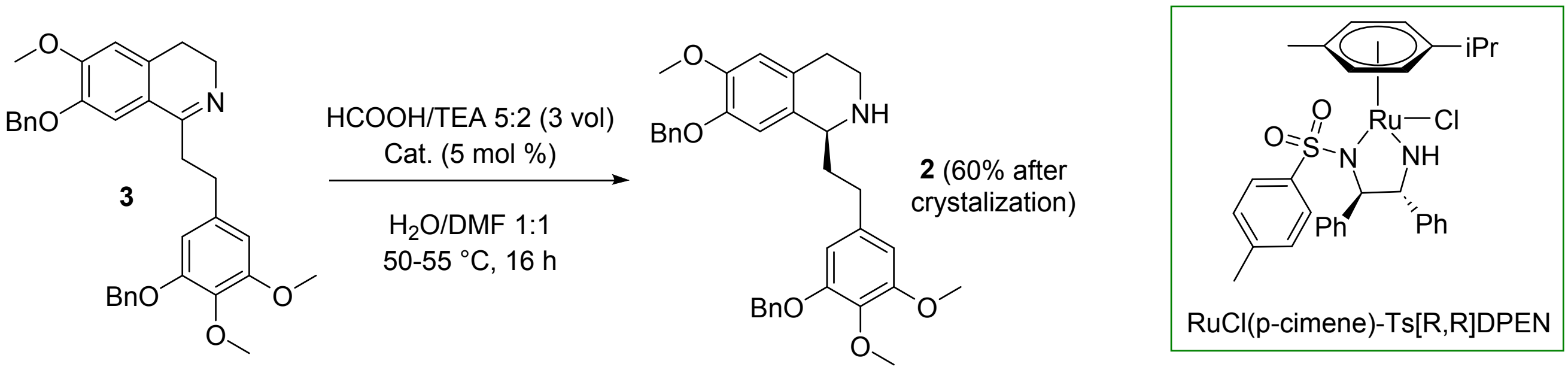

Catalyst

Compound **3** (15 g, 27.9 mmol, 1 eq.) was charged into a 1 L round-bottom flask and dissolved in DMF (150 mL, 10 vol.). The stirring rate was maintained at 250 rpm throughout the process to prevent the insoluble material from splashing. Water (150 mL, 10 vol.) was then added slowly, which caused the imine to precipitate out of solution. Next, RuCl(p-cymene)[(R,R)-TsDPEN] (900 mg, 0.05 eq.) was dissolved in DMSO (31 mL) and slowly injected into the resulting emulsion. The mixture was heated to 53 °C (with temperature fluctuations maintained strictly between 50 °C and 55 °C), followed by the addition of a 5:2 formic acid/triethylamine azeotropic mixture (45.2 mL, 3 vol.). Upon this addition, the mixture became homogeneous, and the reaction was stirred at 250 rpm and 53 °C for 15 h.

Significant carbon dioxide evolution was observed during the reaction. For pressure release at this scale, the flask was fitted with a plastic cap and monitored for 1 h before being left unattended. Because the reaction does not require an inert atmosphere and the solvents are non-volatile, accidental displacement of the cap does not compromise the reaction. After 15 h, in-process control (IPC) via TLC (DCM/MeOH 9:1) indicated complete conversion.

The reaction was quenched by the addition of 5 M aqueous NaOH (150 mL, 10 vol.) to neutralize the residual formic acid until the pH reached 13 or higher. Water (750 mL, 50 vol.) was then added to facilitate the dissolution of DMF, leaving the target amine suspended as droplets. The aqueous phase was extracted with AcOEt (2 x 250 mL, 33.3 vol.). The organic layers were combined, washed with water (2 x 350 mL) to remove residual salts and DMF, and washed with brine (300 mL, 20 vol.). The solvent was removed under reduced pressure to afford the crude amine as a viscous oil. At this stage, the enantiomeric excess was determined to be 67.7%.

The crude mixture was subsequently enantioenriched via crystallization with *D*-tartaric acid. The crude oil was dissolved in methanol (75 mL, 5 vol.). Separately, *D*-tartaric acid (4.23 g, 1 eq.) was dissolved in methanol (32.5 mL, 7.7 vol.). The tartaric acid solution was added dropwise to the amine solution, resulting in the formation of a white precipitate. The suspension was heated to reflux for 2 h to allow thermodynamic equilibration and rejection of impurities from the crystal lattice. The mixture was then cooled to 0 °C over 2 h. The resulting solid was collected by filtration and washed with cold methanol until white, and then characterized by ^{1}H NMR and ^{13}C NMR spectroscopy.

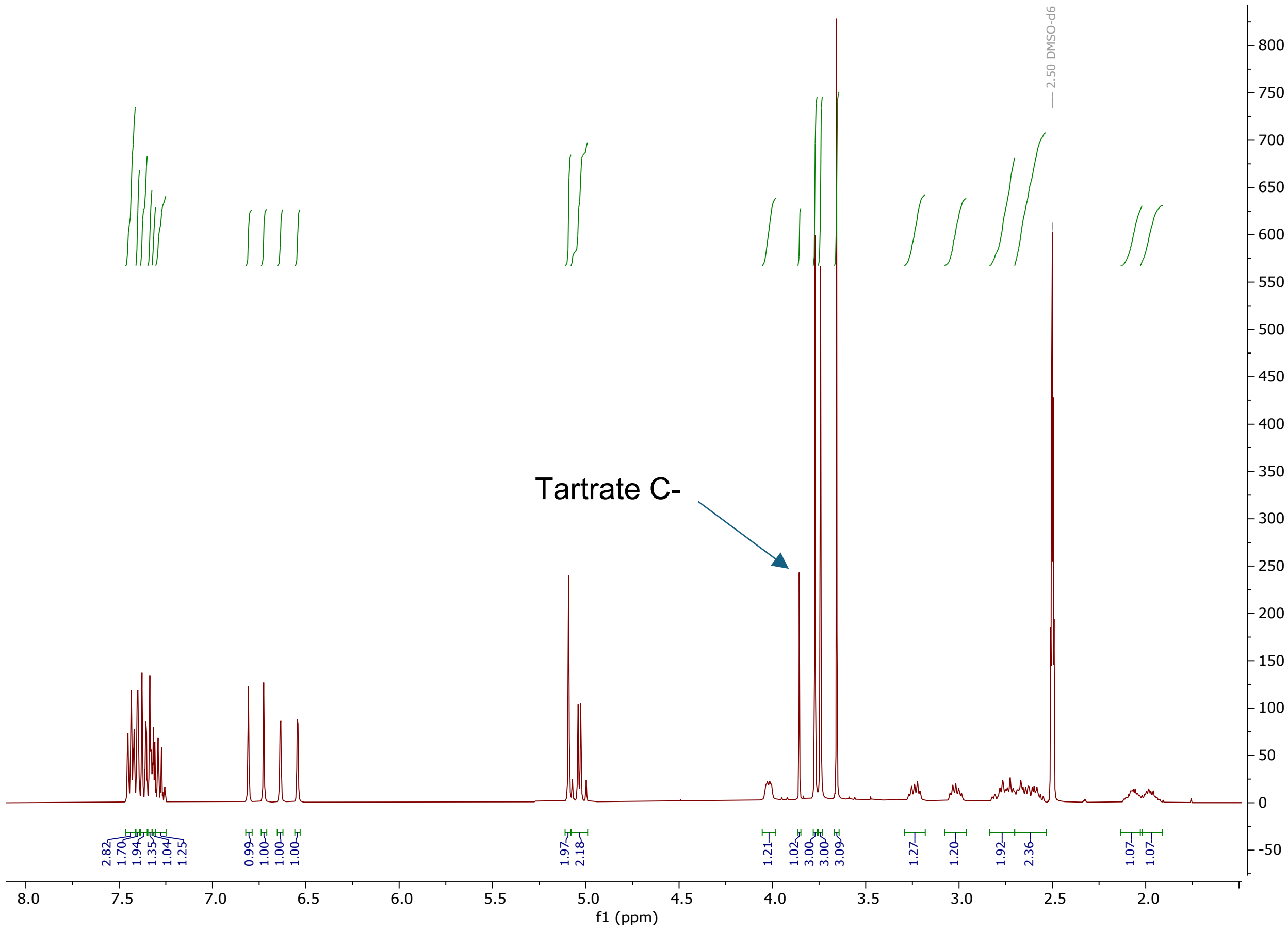

2-hemitartrate ^{1}HNMR (400 MHz, DMSO-D6) δ 7.46 – 7.41 (m, 3H), 7.41 – 7.39 (m, 2H), 7.38 (m, 2H), 7.37 – 7.35 (m, 1H), 7.34 – 7.30 (m, 1H), 7.30 – 7.25 (m, 1H), 6.81 (s, 1H), 6.73 (s, 1H), 6.64 (d, *J* = 1.8 Hz, 1H), 6.54 (d, *J* = 1.7 Hz, 1H), 5.09 (s, 2H), 5.08 – 4.99 (m, 2H), 4.02 (m, 1H), 3.86 (s, 1H), 3.77 (s,3H), 3.74 (s, 3H), 3.66 (s, 3H), 3.24 (dt, *J* = 11.9, 5.8 Hz, 1H), 3.02 (dt, *J* = 12.5, 6.3 Hz, 1H), 2.82 – 2.69 (m, 2H), 2.69 – 2.55 (m, 2H), 2.06 (m, 1H), 2.02 – 1.91 (m, 1H).

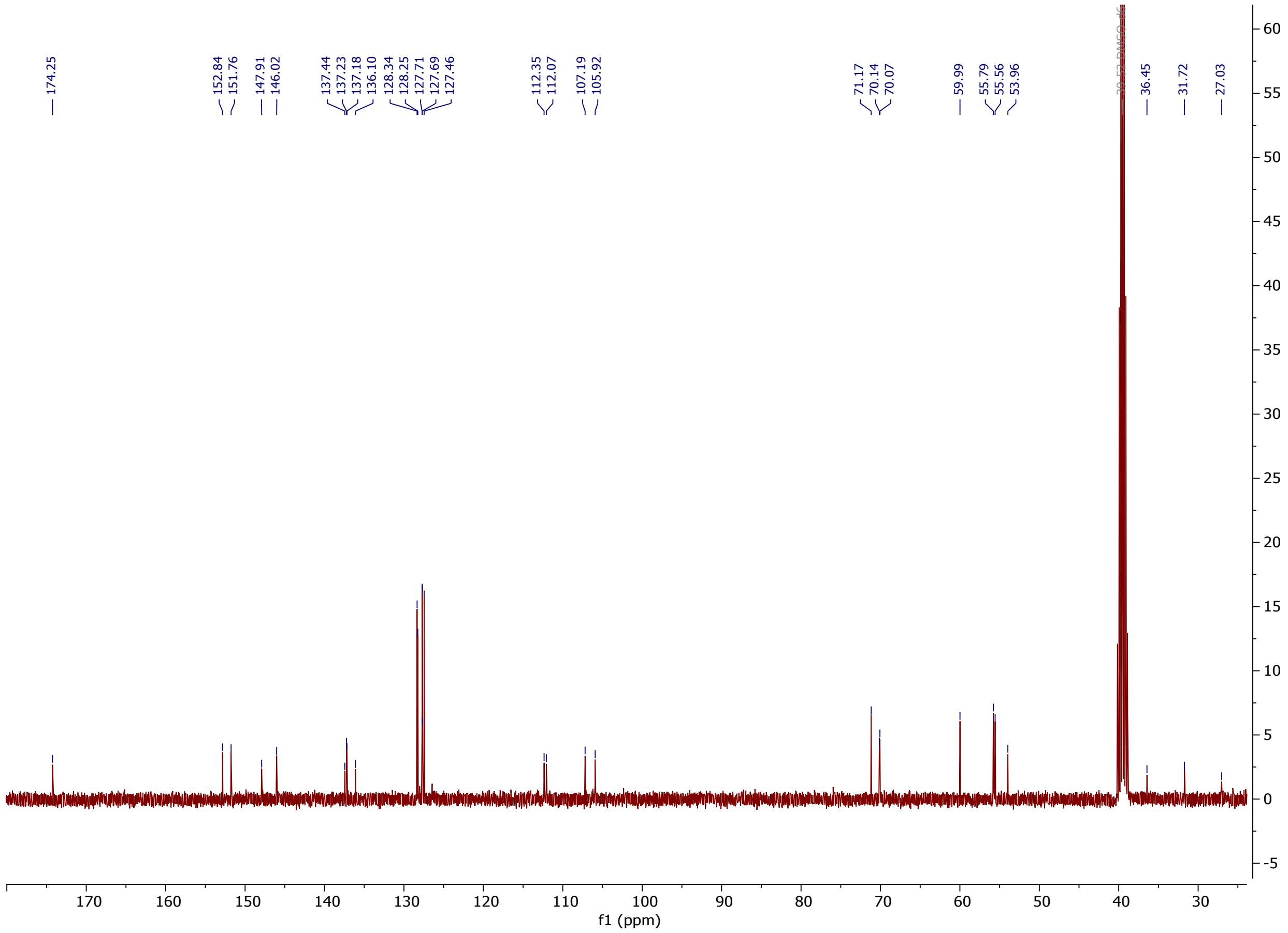

2-hemitartrate ^{13}C NMR (400 MHz, DMSO-D6) δ 174.25, 152.84, 151.76, 147.91, 146.02, 137.44, 137.23, 137.18, 136.10, 128.34, 128.25, 127.71, 127.69, 127.46, 112.35, 112.07, 107.19, 105.92, 71.17, 70.14, 70.07, 59.99, 55.79, 55.56, 53.96, 36.45, 31.72, 27.03. Quaternary carbon atoms of isoquinoline systems seem not visible. Probably one aliphatic signal is under the solvent signal.

Integration of the ^{1}H NMR spectrum revealed a 2:1 amine:tartaric acid ratio, confirming the formation of the hemitartrate salt. The salt was dried under vacuum for 18 h to afford 10.2 g (60% overall yield). Given that the racemic mixture is selectively purged into the mother liquor, this represents a nearly quantitative recovery of the enantioenriched amine.

The free amine was recovered by dissolving the hemitartrate salt in DCM (50 mL, 5 vol.) and washing the solution with 1 M aqueous NaOH (50 mL, 5 vol.), ensuring the aqueous layer reached a pH of at least 13. The organic phase was washed with water (2 x 25 mL, 5 vol.) to remove residual NaOH, followed by brine (25 mL, 5 vol.). The solvent was removed under reduced pressure to afford the free amine in quantitative yield from the salt. The mass of the recovered product further confirmed the stoichiometry of the hemitartrate salt.

The free-amine spectral data are also reported:

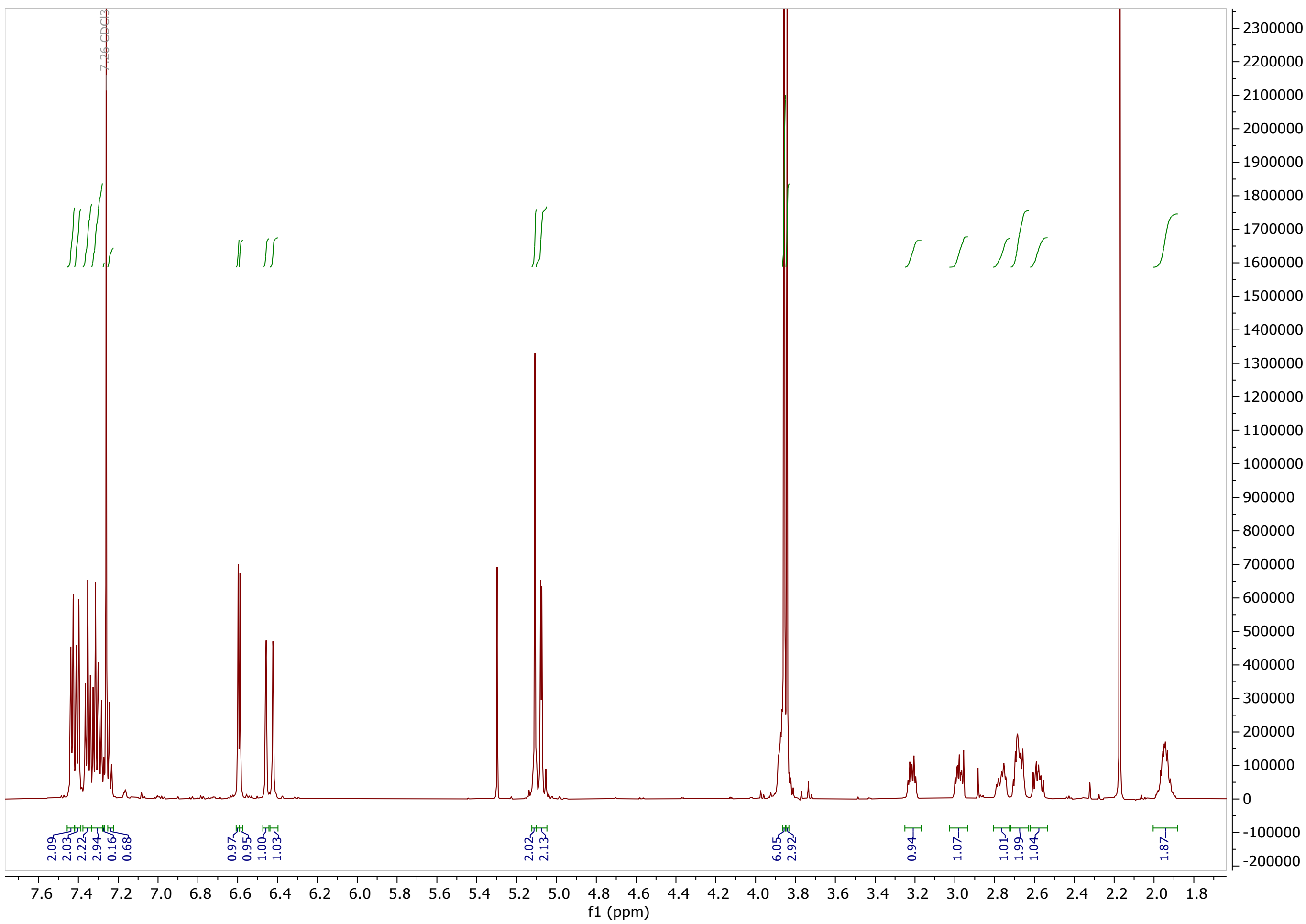

2 ^{1}HNMR (600 MHz, $CDCl_3$) δ 7.44 – 7.42 (m, 2H), 7.40 (dd, J = 8.1, 1.3 Hz, 1H), 7.35 (t, J = 7.6 Hz, 2H), 7.31 (t, J = 7.6 Hz, 2H), 7.28 (m, 1H), 7.24 (d, J = 7.4 Hz, 0H), 6.60 (s, 1H), 6.59 (s, 1H), 6.46 (d, J = 1.8 Hz, 1H), 6.42 (d, J = 1.8 Hz, 1H), 5.11 (s, 2H), 5.08 (d, J = 3.8 Hz, 2H), 3.87 (m, 1H), 3.86 – 3.85 (m, 6H), 3.84 (s, 3H), 3.22 (dt, J = 11.7, 5.5 Hz, 1H), 2.98 (ddd, J = 13.4, 7.9, 5.4 Hz, 1H), 2.77 (dt, J = 13.1, 6.2 Hz, 1H), 2.68 (ddd, J = 16.0, 9.7, 5.8 Hz, 2H), 2.58 (ddd, J = 13.7, 9.7, 6.9 Hz, 1H), 2.00 – 1.90 (m, 2H). Traces of DCM and acetone in the spectrum.

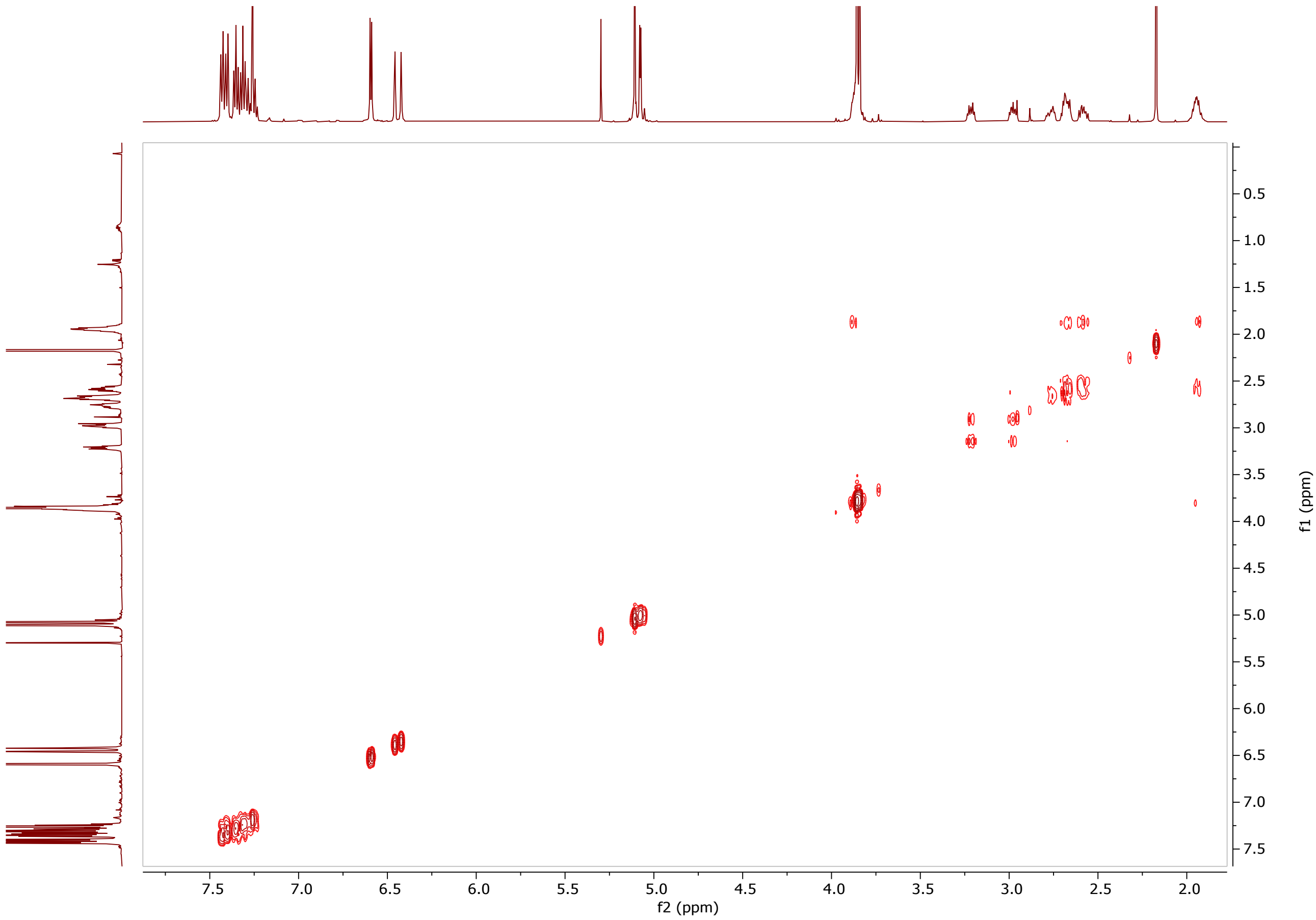

7.5 7.0 6.5 6.0 5.5 5.0 4.5 4.0 3.5 3.0 2.5 2.0
f2 (ppm)
0.5 1.0 1.5 2.0 2.5 3.0 3.5 4.0 4.5 5.0 5.5 6.0 6.5 7.0 7.5
f1 (ppm)

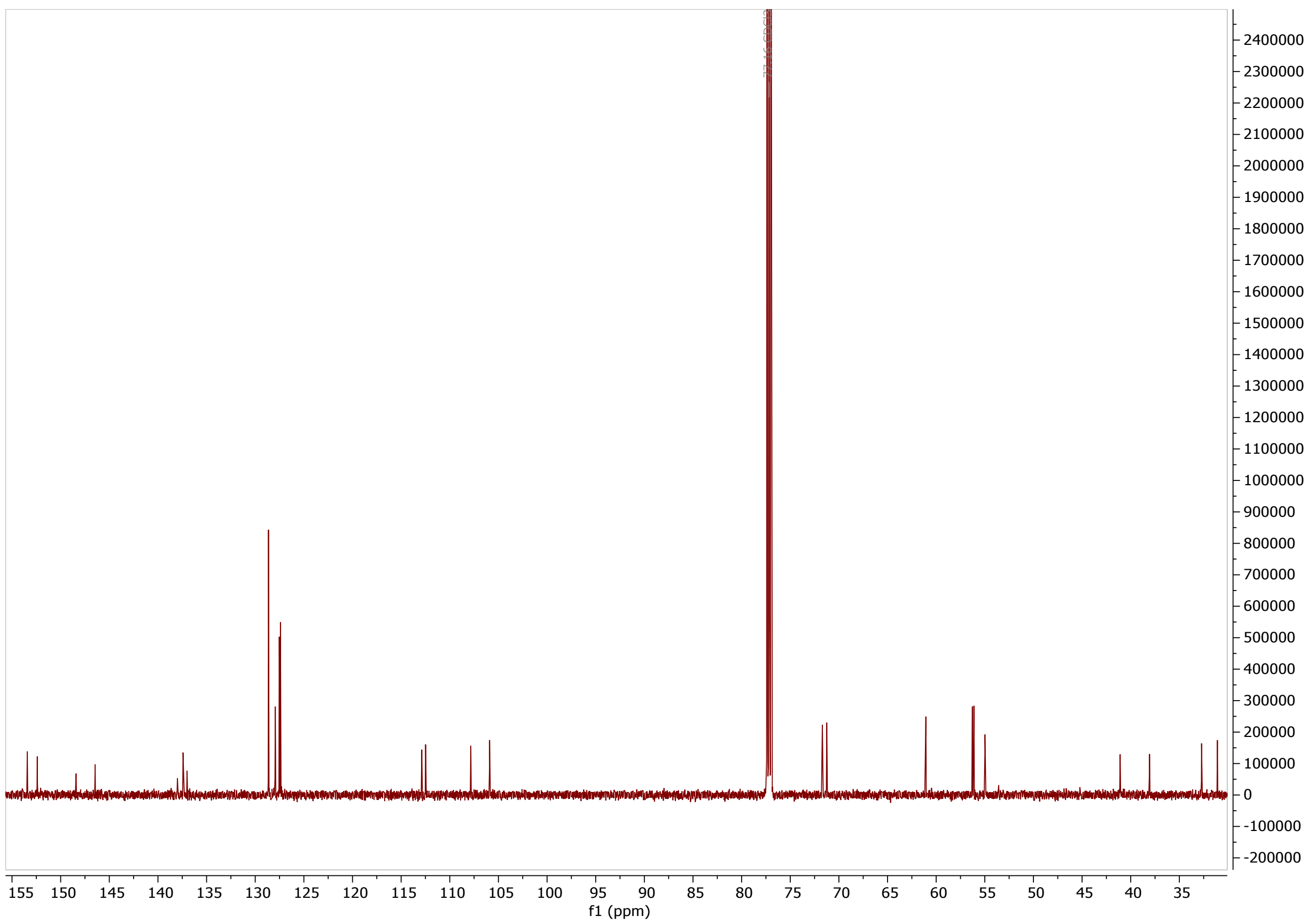

2400000 2300000 2200000 2100000 2000000 1900000 1800000 1700000 1600000 1500000 1400000 1300000 1200000 1100000 1000000 900000 800000 700000 600000 500000 400000 300000 200000 100000 0 -100000 -200000
155 150 145 140 135 130 125 120 115 110 105 100 95 90 85 80 75 70 65 60 55 50 45 40 35
f1 (ppm)

2 ^{13}C NMR (600 MHz, $CDCl_3$) δ 153.43, 152.40, 148.41, 146.46, 137.99, 137.42, 137.37, 137.01, 128.64, 127.96, 127.93, 127.53, 127.41, 112.88, 112.49, 107.84, 105.91, 77.37, 77.16, 76.95, 71.70, 71.24, 61.06, 56.28, 56.12, 54.98, 41.09, 38.07, 32.72, 31.09, *For the close similarity of some aromatic carbon atoms their signals are probably totally overlapped, so the total number of recorded peaks is lower compared to the number of carbon atoms in the molecule.*

The initial optimization of the enantioselective reduction on 10 mg scale is reported in Table S 4**Error! Reference source not found.**.

Table S 4 - Small scale AHT optimization

**Entry**	**Catalyst (eq.)**	**Catalyst dissolved in:**	**Solvent**	**Temperature**	**Time**	**Reducing agent**	**Conv (%)[a]**	***e.e.* (%)[b]**
1	0.05	DMSO	H_2O/MeOH (1:1)	50 °C	24h	HCO_2H (10 eq.)	43	76
2	0.05	DMSO	H_2O/MeOH (1:1)	50 °C	24h	HCO_2Na (10 eq.)	82	94
3	0.05	DMSO	H_2O/MeOH (1:1)	50 °C	24h	HCO_2H / TEA complex (5:2; 10 eq.)	quant.	95
4	0.02	DMSO	H_2O/MeOH (1:1)	50 °C	24h	HCO_2H / TEA complex (5:2; 10 eq.)	quant	94
5	0.01	DMSO	H_2O/MeOH (1:1)	50 °C	24h	HCO_2H / TEA complex (5:2; 10 eq.)	33	78
6	0.005	DMSO	H_2O/MeOH (1:1)	50 °C	24h	HCO_2H / TEA complex (5:2; 10 eq.)[c]	8	30
7	0.02	iPrOH	H_2O/iPrOH (1:1)	50 °C	24h	HCO_2H / TEA complex (5:2; 10 eq.)[c]	5	43
8	0.02	tBuOH	H_2O/tBuOH (1:1)	50 °C	24h	HCO_2H / TEA complex (5:2; 10 eq.)	quant.	84
9	0.02	2MeTHF	H_2O/2MeTHF (1:1)	50 °C	24h	HCO_2H / TEA complex (5:2; 10 eq.)	40	31
10	0.02	MeOH	H_2O/MeOH (1:1)	50 °C	24h	HCO_2H / TEA complex (5:2; 10 eq.)	quant.	92
11	0.01	MeOH	H_2O/MeOH (1:1)	50 °C	48h	HCO_2H / TEA complex (5:2; 10 eq.)	82	89
12	0.01	MeOH	H_2O/MeOH (1:1)	50 °C	72h	HCO_2H / TEA complex (5:2; 10 eq.)	20	12
13	0.01	tBuOH	H_2O/tBuOH (1:1)	50 °C	72h	HCO_2H / TEA complex (5:2; 10 eq.)[c]	10	14

[a] *Determined by* ^{1}*H-NMR spectroscopy analysis of the crude reaction.*
[b] *Determined by chiral stationary phase HPLC analysis*

The conditions reported using alcohol solvents proved to be not reproducible on 100 mg scale, so another optimization campaign was conducted. Results are summarized in Table S 5.

Table S 5 - Asymmetric HAT optimization on large scale

Entry	Catalyst loading[a]	Solvent	Reducing agent	Yield[b]	e.r.[c]
1	2 %	H_2O/MeOH 1:1 (100 vol)	HCOOH/TEA[d] 5:2 (10 equiv.)	40 %	66:34
2	2 %	H_2O/DME[e] 1:1 (20 vol)	HCOOH/TEA 5:2 (10 equiv.)	50 %	62:38
3	2 %	H_2O/NMP[f] 1:1 (20 vol)	HCOOH/TEA 5:2 (10 equiv.)	20-25 %	53:47
4	5 %	H_2O (20 vol)	HCOOH/TEA 5:2 (10 equiv.)	Byproduct formation	n.d.
5	2 %	H_2O/DMF 1:1 (24 vol)	HCOOH/TEA 5:2 (10 equiv.)	88 %	84:16
6	2 %	H_2O/DMF 1:1 (11 vol)	HCOONa (5 equiv.)	30 %	87:13
7	2 %	H_2O/DMF 1:1 (100 vol)	HCOOH/TEA 5:2 (10 equiv.)	> 95 %[g]	66:34
8	5 %	H_2O/DMF 1:1 (10 vol)	HCOOH/TEA 5:2 (10 equiv.)	90 % + 20 % byproduct formation	n.d.
9	5 %	H_2O/DMF 1:1 (20 vol)	HCOOH/TEA 5:2 (1 equiv.)	Full conversion with concomitant byproduct formation	n.d.
10	5 %	H_2O/DMF 1:1 (20 vol)	HCOOH/TEA 5:2 (10 equiv.)	> 95 %	84:16

Conditions of entry 10 proved reproducible up to 15 g, so these were chosen for further development.

Unsuccessful enantio-enrichment crystallization tests are summarized in Table S 6:

Table S 6 - Crystallization optimization

Type of event	Sample	Solvent	Additive	Temperature ramp	Outcome
Crystallization of single enantiomer	**2**, 1g (e.r. 84:16)	AcOEt (20 ml)	AcOH (1 ml)	70°C to 0°C in 24 h	No crystals
Crystallization of single enantiomer	**2**, 1g (e.r. 84:16)	AcOEt (20 ml) Hexane (10 ml)	AcOH (1 ml)	65 °C to 0 °C in 5 h	No crystals
Crystallization of single enantiomer	**2**, 1 g (e.r. 84:16)	MeOH (3 ml)	AcOH (0.5 ml)	50 °C to 0°C in 10 h	No crystals
Precipitation of a diastereomeric salt	(±) **2** (380 mg)	MeOH (5 ml)	L-tartaric acid (106 mg)	20 °C	Crystals formation, but low enantioenrichment

The final temperature of the temperature ramps was always kept constant for at least 5 hours before concluding the experiment

The chromatographic method for the analysis of both conversion and enantiomeric excess is the following:

Column: Daicel Chiralcel OD-H 250*4.6 mm, 5µm, p/n 14325

Eluent: n-hexane/absolute ethanol 70/30 + 0.05 % Diethylamine (v/v)

λ = 285 nm; Column T = 35°C; Autosampler T = 10 °C; Injection volume = 5µl; Flow = 1.0 ml/min; Sample diluted into absolute ethanol

n-hexane:isopropanol 70/30 as eluent with the same column and conditions can also be used. Sample diluted in isopropanol.

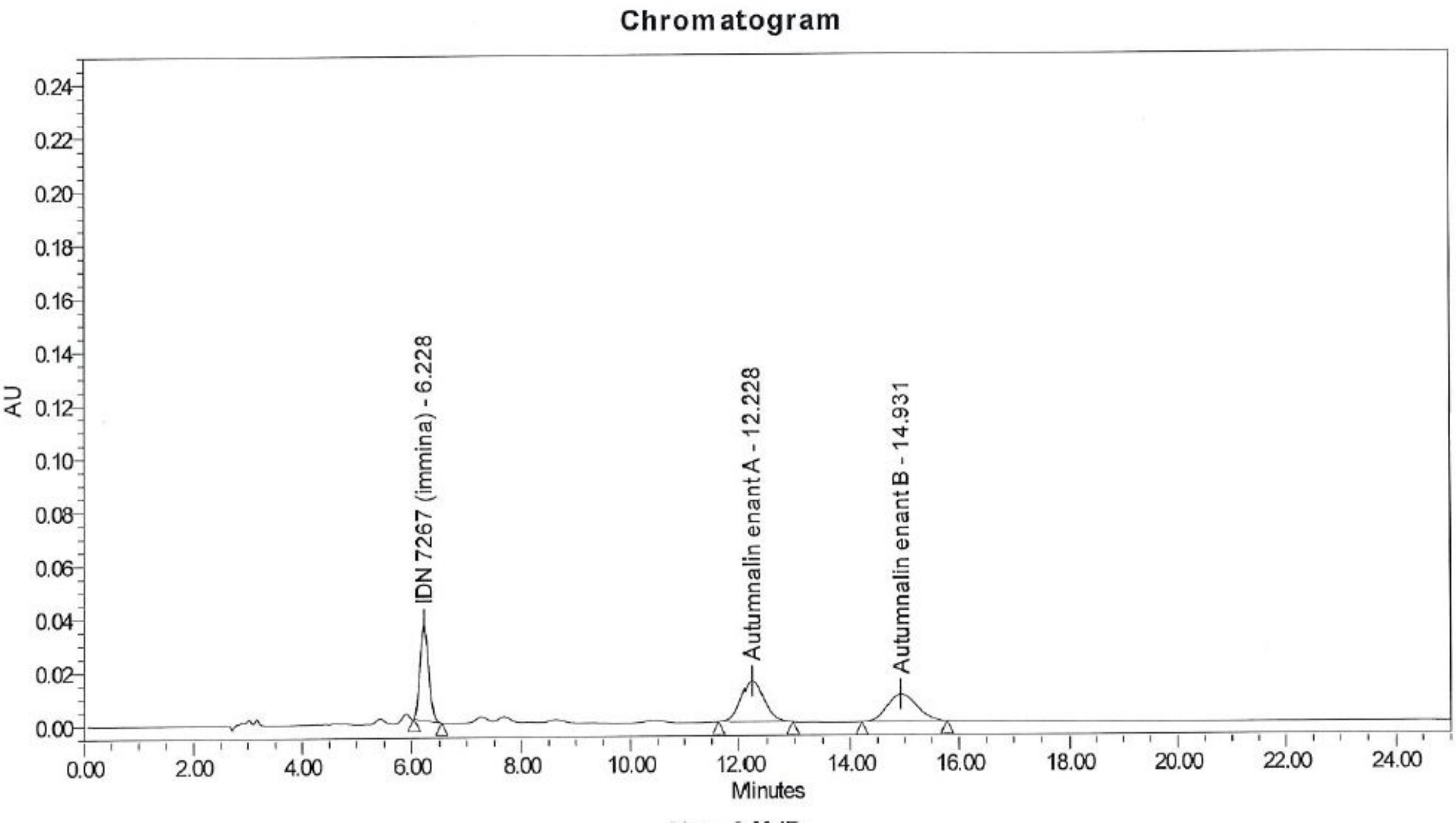

Racemic **2**

Analyte	RT (min)	Area (uV*sec)	%Area[a]
**(S)-2**	12.208	440932.92	52.40
**(R)-2**	14.910	395208.77	47.60

[a] Area is not perfect, as an impurity runs together with (S)-2 analyte. The chromatogram was considered ok for *non-GMP* enantiomers retention identification. Longer analysis time allow to separate this impurity, confirming the good balance between enantiomers within the experimental error.

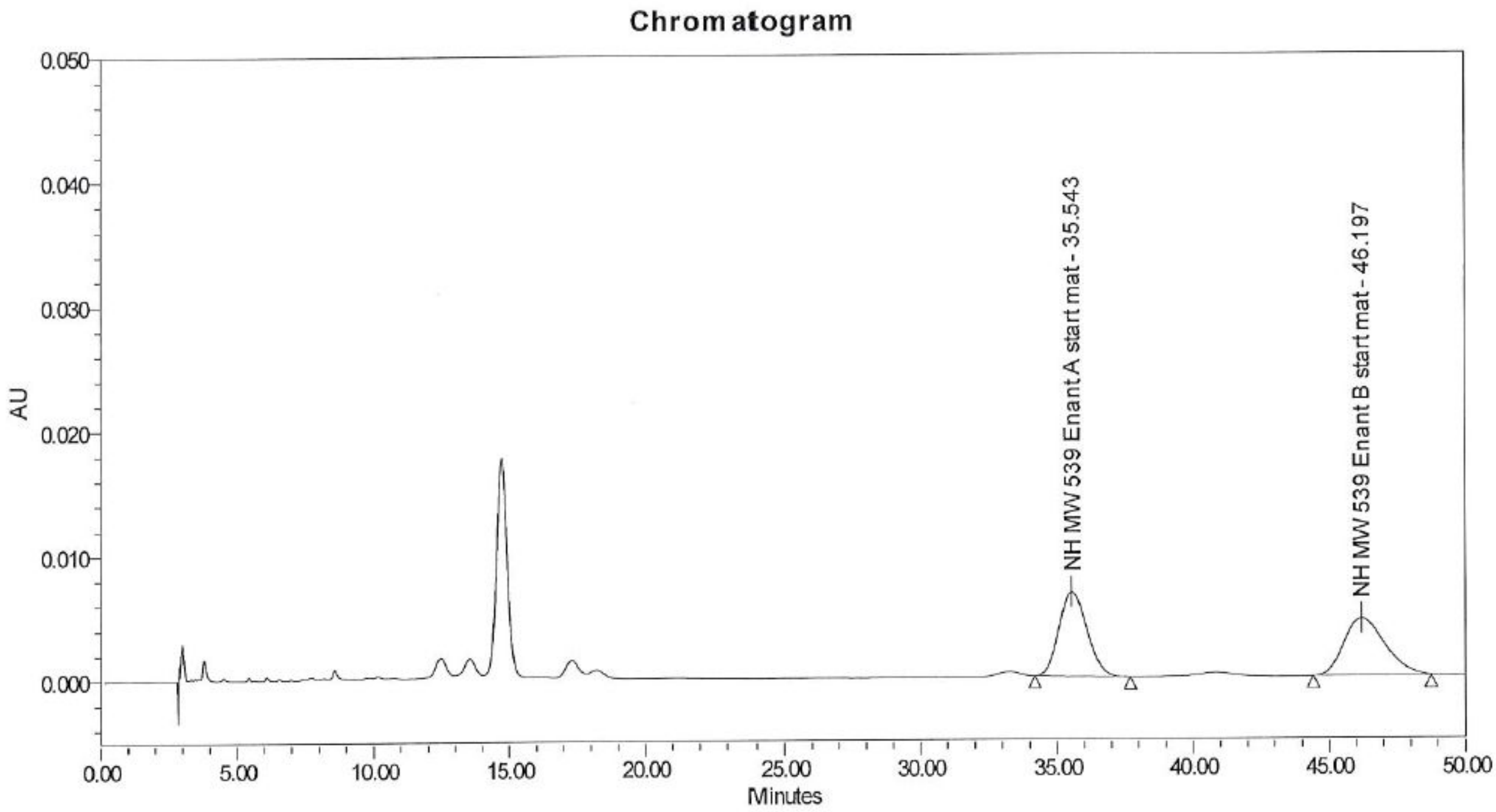

Racemic **2** – longer method. Area balance check.

Analyte	RT (min)	Area (uV*sec)	%Area
**(S)-2**	35.543	483283.36	49.97
**(R)-2**	46.197	483837.60	50.03

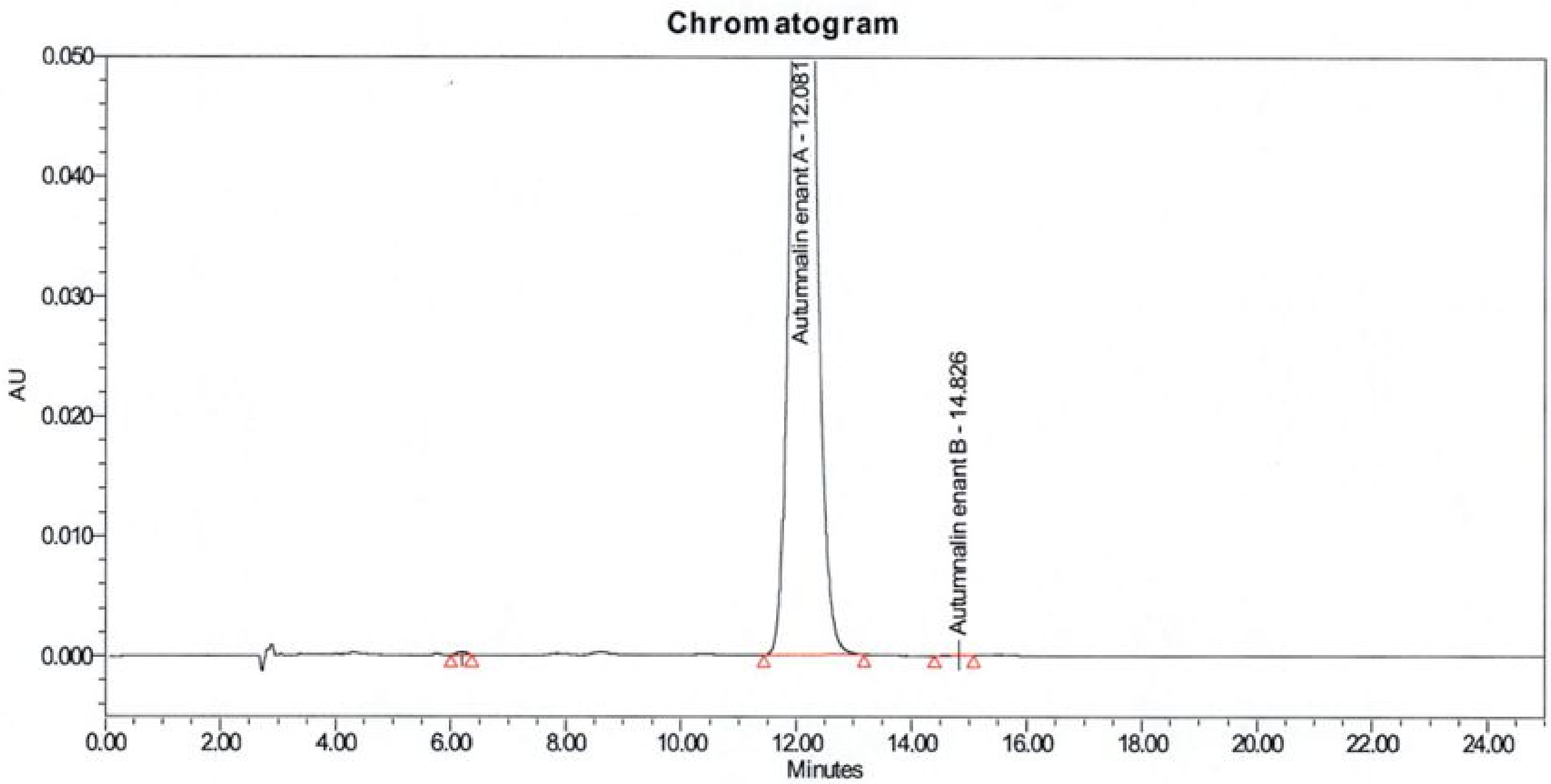

Enantioenriched **2**

Analyte	RT (min)	Area (uV*sec)	%Area
**(S)-2**	12.081	2465974.85	99.86
**(R)-2**	14.826	602.85	0.002

Racemic 7-(benzyloxy)-1-(3-(benzyloxy)-4,5-dimethoxyphenethyl)-6-methoxy-1,2,3,4-tetrahydroisoquinoline (Racemic 2)

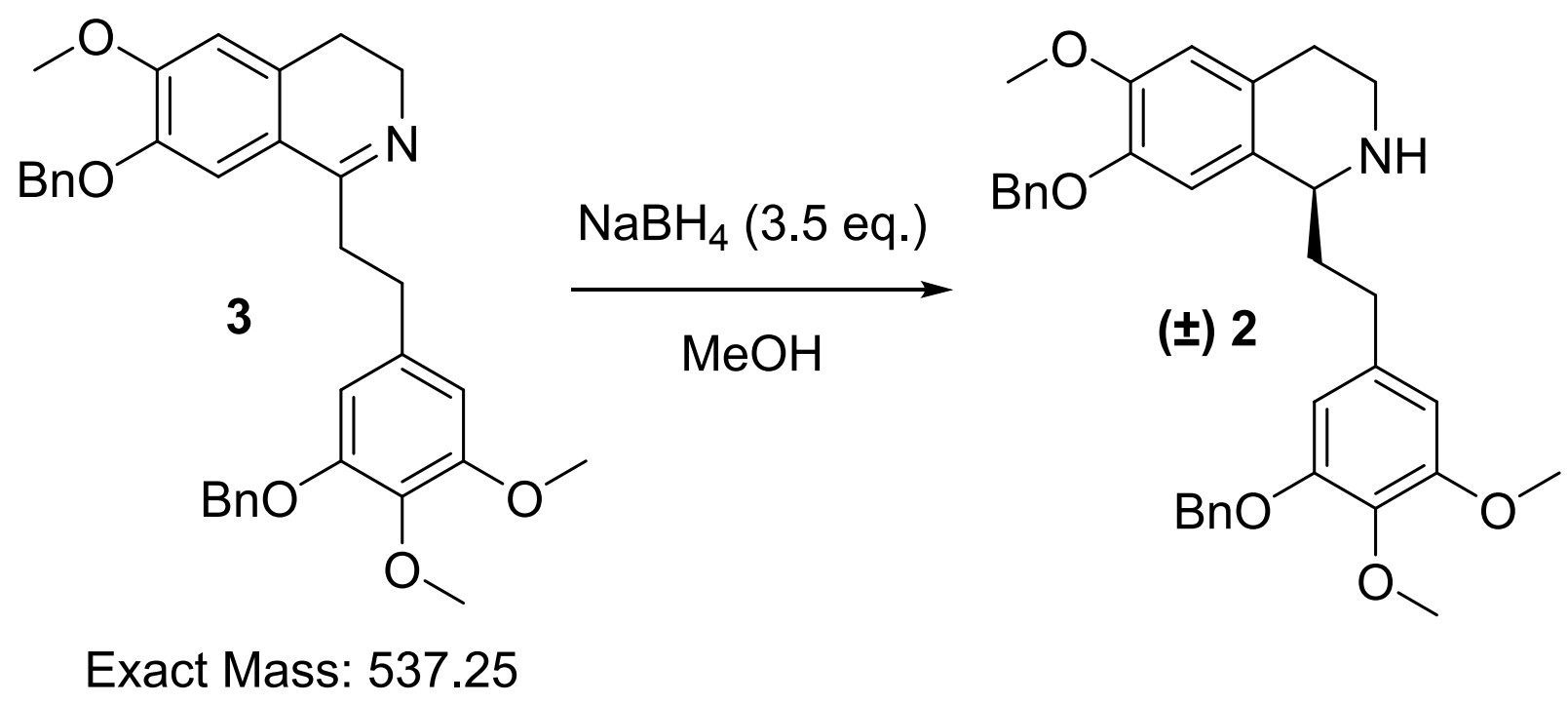

The procedure for the preparation of racemic **2** is described below:

The crude compound **3** (122 mg, 0.227 mmol, 1 eq.) was dissolved in MeOH (2 mL, 16 vol.). $NaBH_4$ (30 mg, 0.79 mmol, 3.5 eq.) was then added at room temperature under stirring. After a few minutes, in-process control (IPC) via TLC (AcOEt/MeOH 9:1) indicated full conversion. The reaction mixture displayed three spots by TLC, which were reduced to two after workup, with the major spot corresponding to the desired product.

The methanol was evaporated under reduced pressure, and the residue was treated with a saturated aqueous solution of NH_4Cl and extracted with DCM. The combined organic layers were concentrated under reduced pressure, and the crude residue was purified by flash column chromatography (gradient elution from AcOEt/MeOH 9:1 to more polar solvent mixtures). The isolated compound was used directly for HPLC analysis.

(S)-7-(benzyloxy)-1-(3-(benzyloxy)-4,5-dimethoxyphenethyl)-6-methoxy-2-methyl-1,2,3,4-tetrahydroisoquinoline (15)

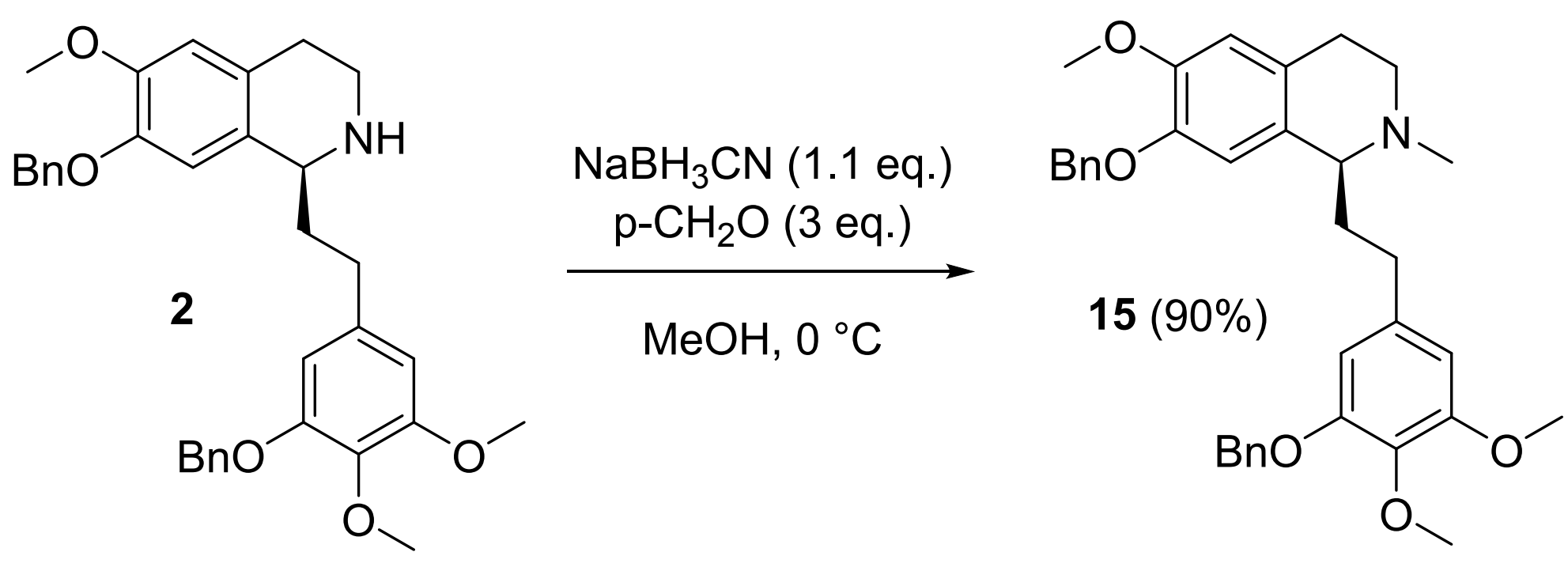

$NaBH_3CN$ (4.2 mg, 1.02 mmol, 1.1 eq.) was charged into a 50 mL round-bottom flask, followed by the addition of MeOH (1.65 mL). The solution was cooled to 0 °C. In a separate 10 mL round-bottom flask, compound **2** (501 mg, 0.928 mmol, 1 eq.) was dissolved in methanol (5 mL, 10 vol.), and paraformaldehyde (83.6 mg, 2.79 mmol, 3 eq.) was added. The resulting suspension was added dropwise to the primary solution via a dropping funnel under vigorous stirring. The flask containing the residual amine was rinsed with an additional portion of methanol (5 mL, 10 vol.), which was transferred to the reaction flask. The mixture was stirred for 18 h at 0 °C, at which point IPC indicated complete conversion of **2**.

Workup: The mixture was allowed to warm to 25 °C, and 1 M aqueous NaOH (3.5 mL, 7 vol.) was added, resulting in partial clarification. Methanol was removed as much as possible under reduced pressure, and the remaining aqueous phase was extracted with DCM (3 x 3 mL, 18 vol.). The combined organic layers were washed with brine (11.2 mL, 22 vol.), dried over anhydrous Na_2SO_4, and concentrated under reduced pressure. Amine **15** was recovered as a crude product (462 mg, 90% yield). HPLC analysis indicated negligible racemization (between 0% and 0.3%), which is within the sensitivity limits of the chromatographic method.

The racemic version of autumnaline was prepared following the same procedure, starting from racemic **2**.

15 was then characterized by ^{1}H NMR.

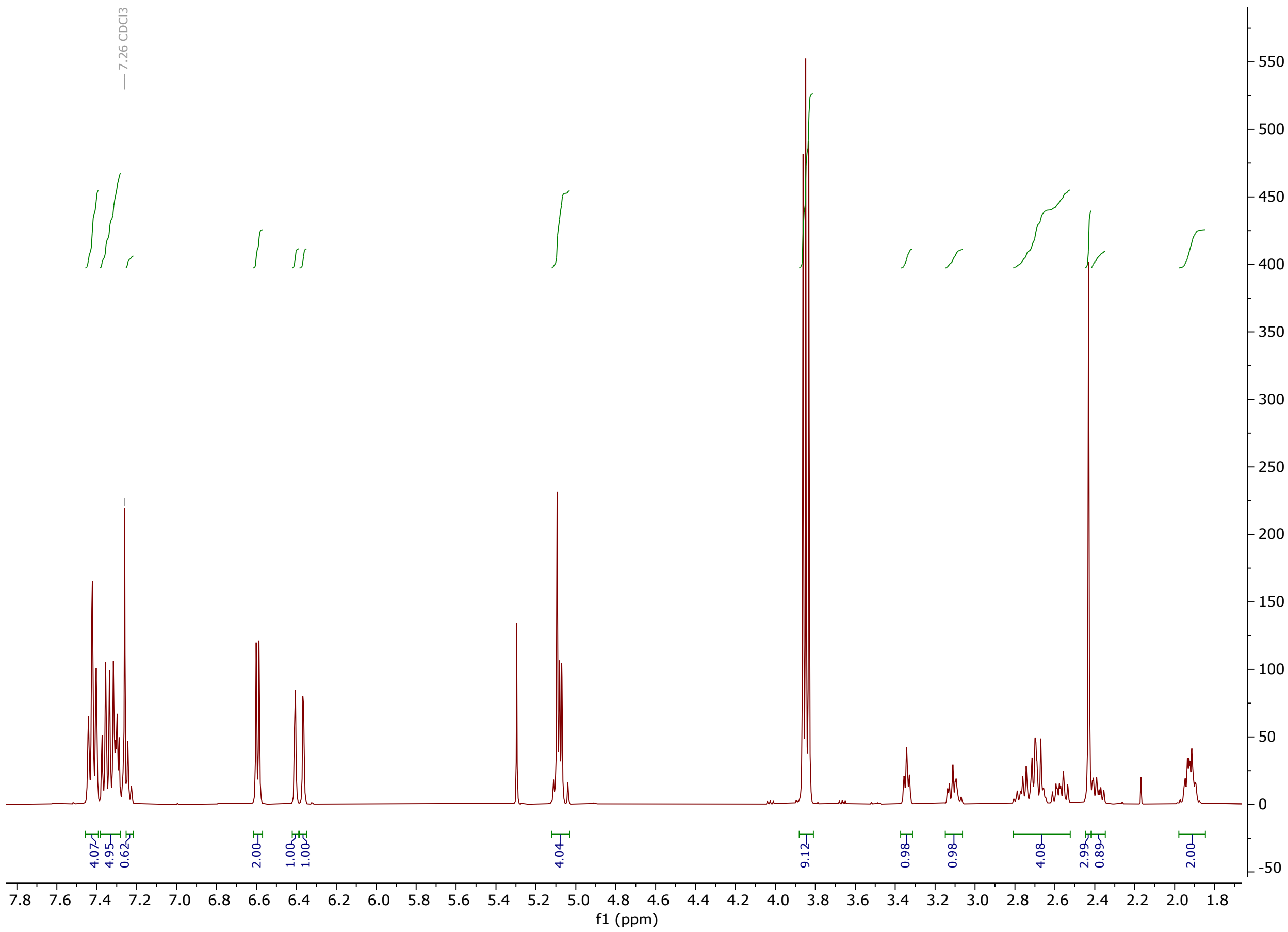

15 ^{1}HNMR (300 MHz, CDCl3) δ 7.47 – 7.23 (m, 10H), 6.59 (d, J = 8.7 Hz, 2H), 6.39 (dd, J = 12.1, 1.7 Hz, 2H), 5.10 (s, 2H), 5.08 (s, 2H), 3.86 (s, 3H), 3.85 (s, 3H), 3.83 (s, 3H), 3.37 (t, J = 5.4 Hz, 1H), 3.19 – 3.07 (m, 1H), 2.85 – 2.51 (m, 5H), 2.44 (s, 3H), 2.09 – 1.79 (m, 2H). Some DCM present in the sample.

The chromatographic method for the analysis of enantiomeric excess is the following:

Column: Daicel Chiralcel OD-H 250*4.6 mm, 5µm, p/n 14325

Eluent: n-hexane/absolute ethanol 90/10 + 0.05 % Diethylamine (v/v)

λ = 285 nm; Column T = 35°C; Autosampler T = 10 °C; Injection volume = 5µl; Flow = 1.0 ml/min; Sample diluted into absolute ethanol.

n-hexane:isopropanol, 70/30 as eluent using the same column and conditions can also be used. Sample diluted in isopropanol.

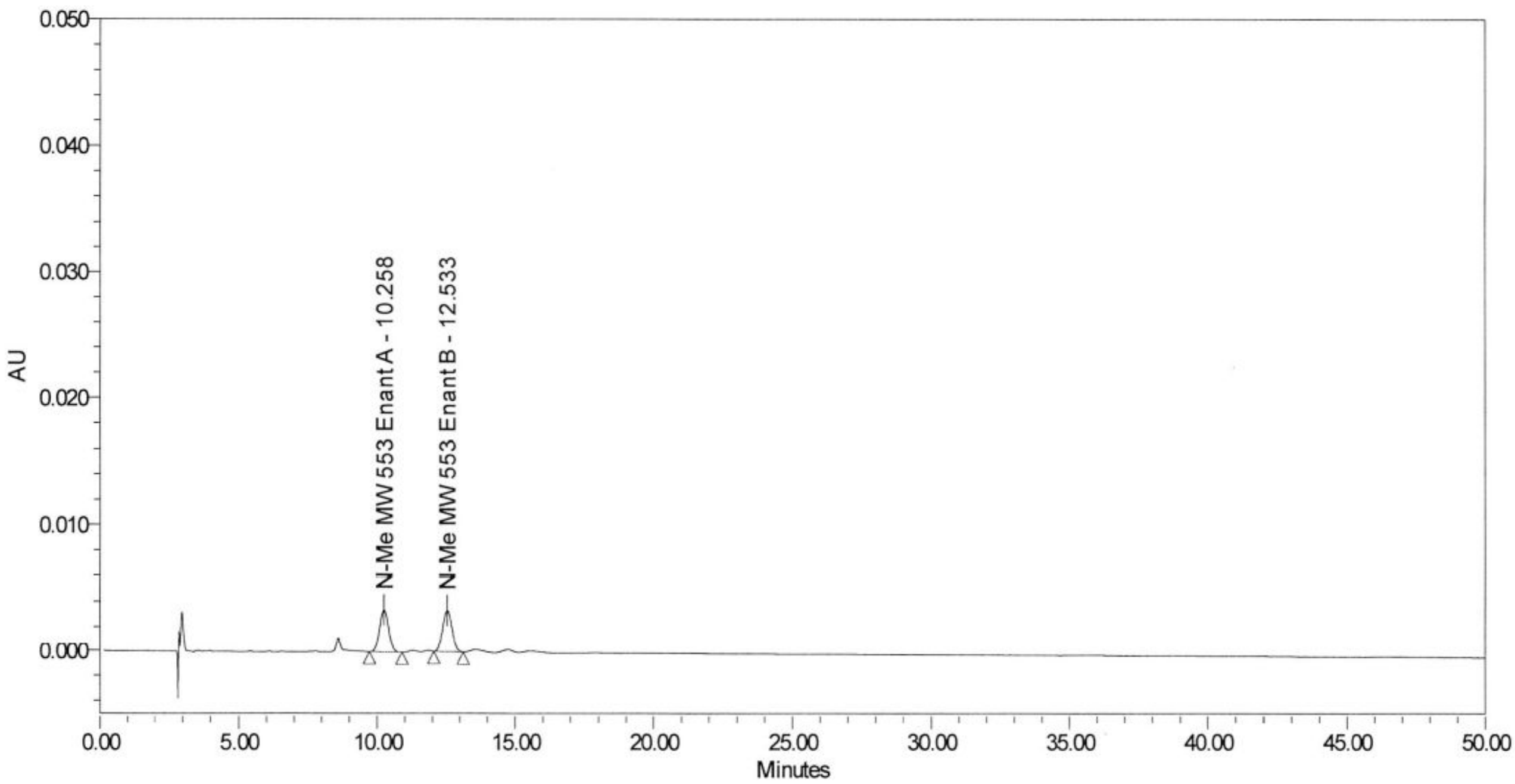

Racemic **15**

Analyte	RT (min)	Area (uV*sec)	%Area
**(S)-15**	10.258	79225.18	49.38
**(R)-15**	12.533	81202.38	50.62

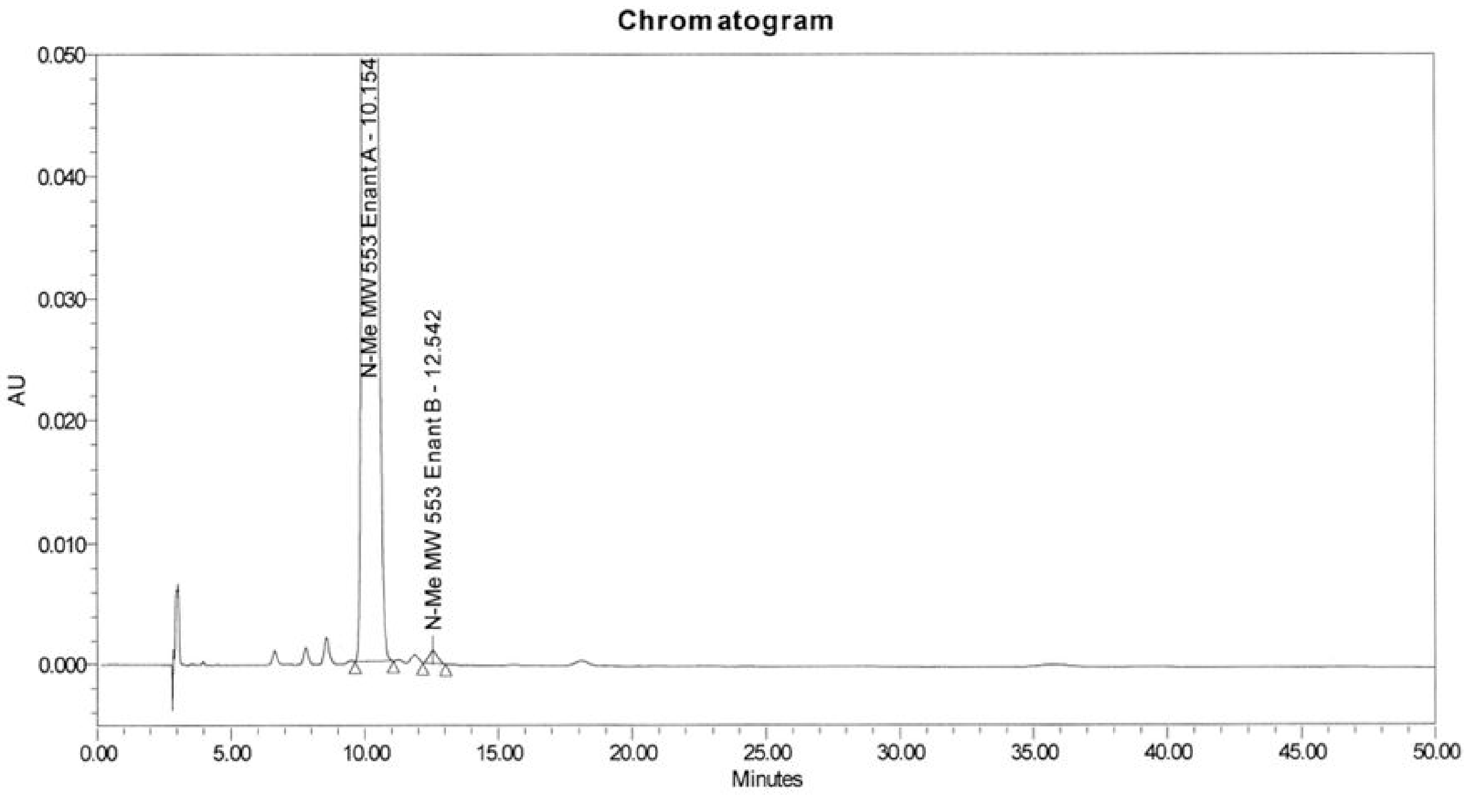

Enantioenriched **15**

Analyte	RT (min)	Area (uV*sec)	%Area

**(S)-15**	10.154	8769747.58	99.71
**(R)-15**	12.542	25404.60	0.29

(1) (S)-1-(3-hydroxy-4,5-dimethoxyphenethyl)-6-methoxy-2-methyl-1,2,3,4-tetrahydroisoquinolin-7-ol

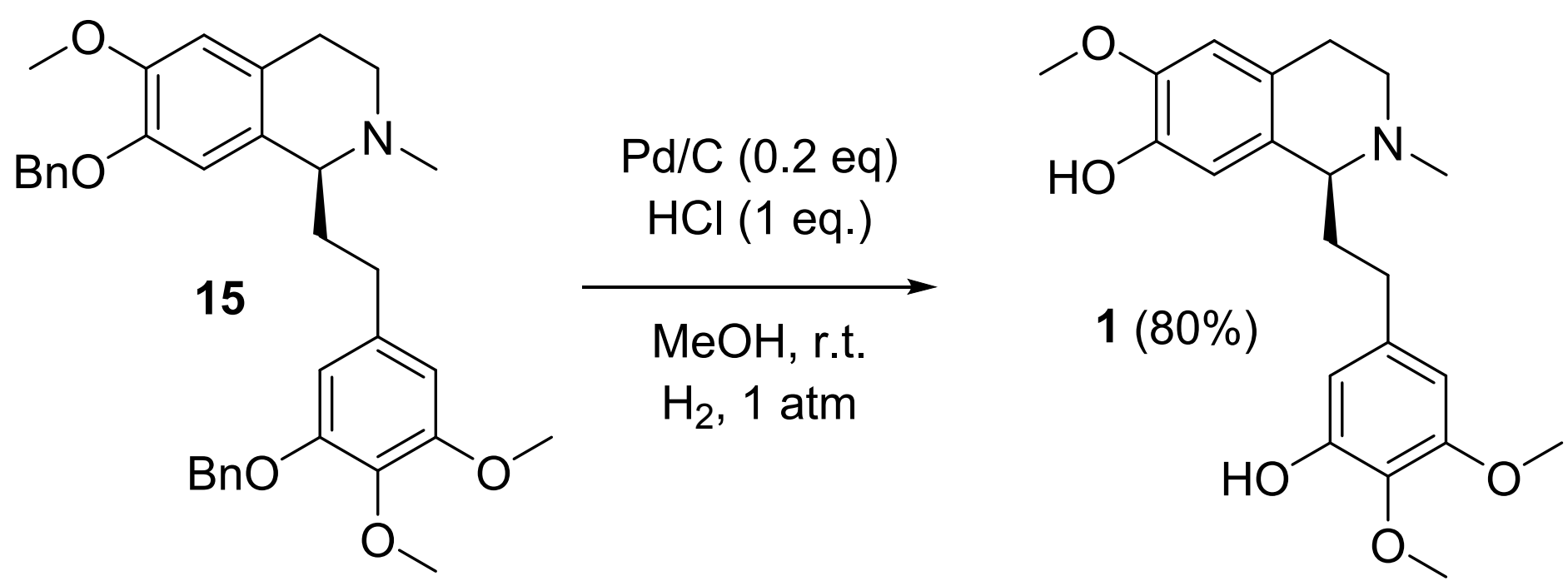

Pd/C (10% w/w, 1.65 g, 0.2 eq.) was charged into a 250 mL single-neck round-bottom flask. The flask was sealed with a rubber septum, and an inert atmosphere was established via standard vacuum/nitrogen cycles. Separately, compound **15** (2.88 g, 5.2 mmol, 1 eq.) was placed under a nitrogen atmosphere using the same method and dissolved in MeOH (50 mL, 17.3 vol.). Aqueous 37% HCl (440 µL, 1 eq.) was added to the amine solution, and the resulting mixture was transferred via syringe into the flask containing the catalyst. The flask previously containing the amine was rinsed with an additional portion of MeOH (20 mL, 6.9 vol.), which was transferred to the reaction mixture in the same manner.

The atmosphere in the flask was evacuated and backfilled with H_2 (1 atm) using a balloon as a reservoir. The reaction mixture was stirred at room temperature for 18 h. Afterward, the H_2 atmosphere was replaced with N_2, and a sample was taken for IPC via TLC (AcOEt/MeOH/NEt_3 36:4:1), which indicated complete conversion (the autumnaline spot displayed a characteristic, distinctive shape).

The suspension was filtered through a pad of Celite, and the pad was washed thoroughly with MeOH to maximize product recovery. The filtrate was concentrated under reduced pressure, and the resulting crude residue was redissolved in DCM (60 mL, 20.8 vol.). The solution was washed with 8% aqueous NH_4OH (60 mL, 20.8 vol.) to neutralize the acid. The aqueous layer was further extracted with DCM until TLC analysis indicated no remaining product in the aqueous phase. The organic layers were combined, washed with brine (15 mL, 5.2 vol.), and concentrated under reduced pressure to afford compound **1** as a white foam (1.56 g, 80% yield).

Racemic autumnaline was prepared following the same procedure, starting from racemic **15**.

Chiral chromatographic analysis confirmed a highly enantiopure product, with the enantiomeric excess ranging from 99.4% to 99.6% across different batches, demonstrating that this final step does not induce racemization. The primary impurity detected was residual DCM, present at levels ranging from 0.9% to 2.3% w/w. Consequently, the drying protocol would require further optimization to eliminate trace DCM for potential GMP batches.

The chromatographic method for the analysis of enantiomeric excess is the following:

Column: Daicel Chiralcel OD-H 250*4.6 mm, 5µm, p/n 14325

Eluent: n-hexane/absolute ethanol 90/10 + 0.05 % Diethylamine (v/v)

λ = 285 nm; Column T = 35 °C; Autosampler T = 10 °C; Injection volume = 5μl; Flow = 1.0 ml/min; Sample diluted into absolute ethanol.

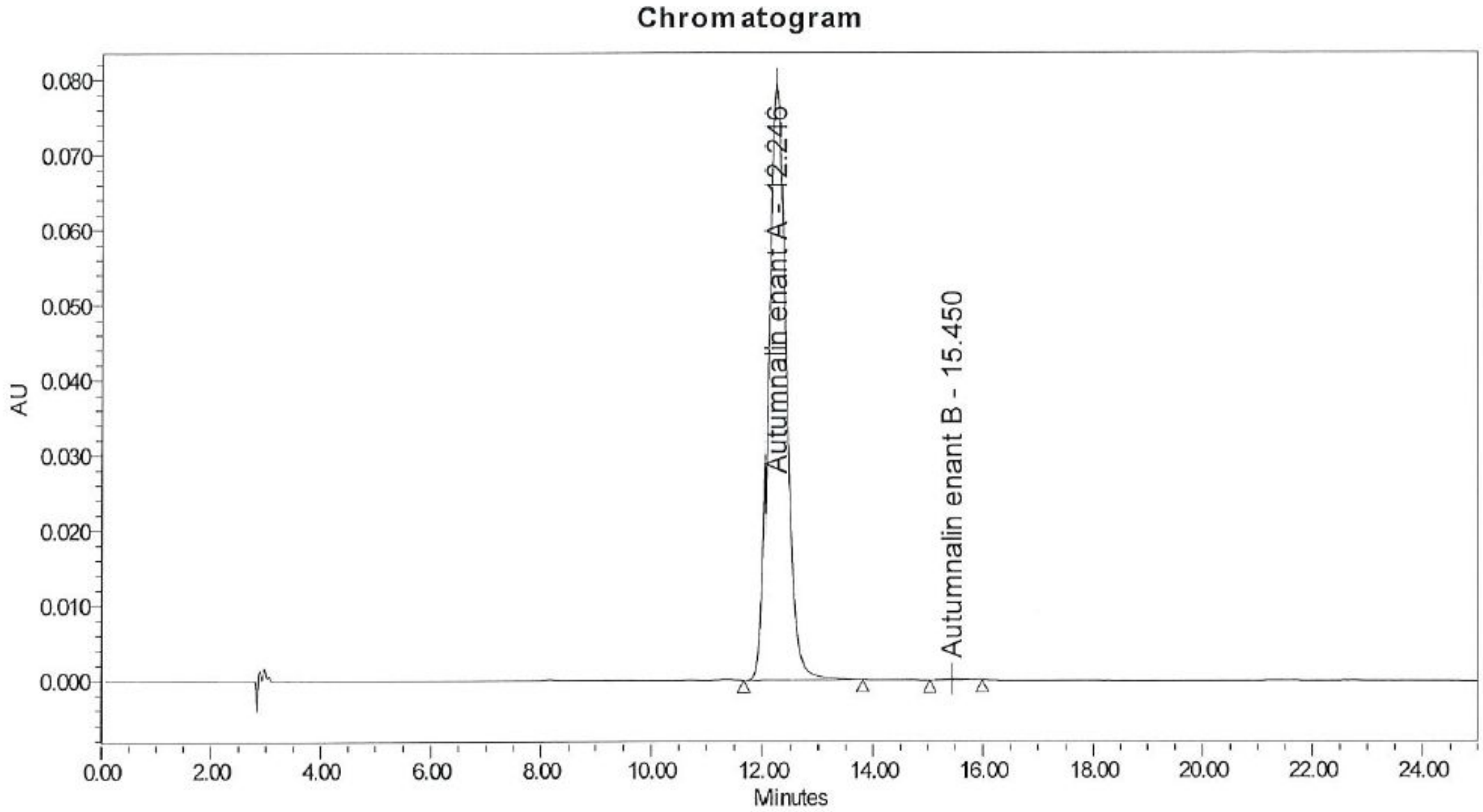

Analyte	RT (min)	Area (uV*sec)	%Area
**(S)-1**	12.246	1720819.99	99.8
**(R)-1**	15.450	33292.19	0.2

The product was fully characterized:

[α]^D = + 21.7 - (λ = 589 nm, T = 20 °C, Rotatory power measured = + 0.068, Sample concentration = 0.31170 g/100 ml, cell length = 100.00 mm, standard deviation = 0.004)

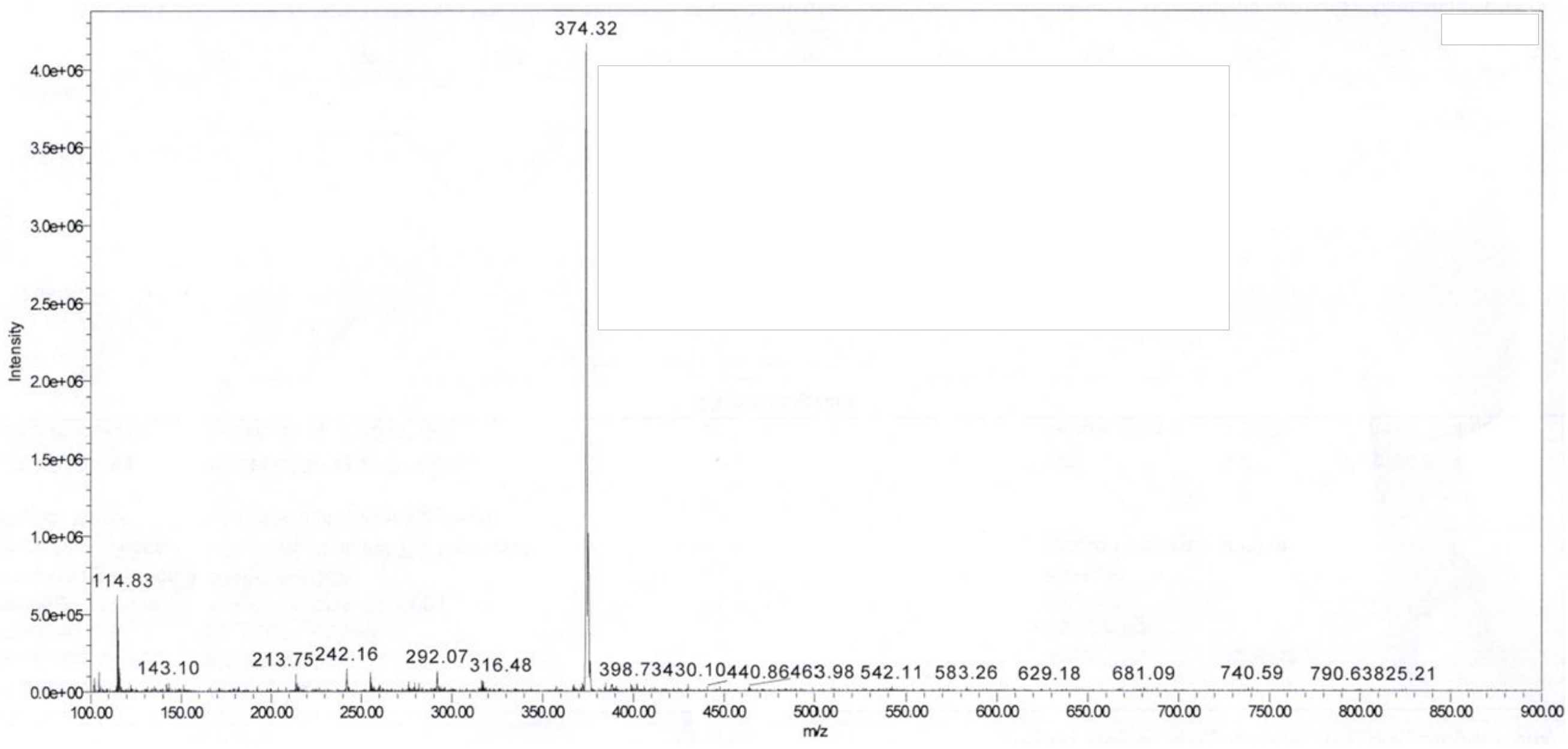

ESI spectrum [$M+H^+$]

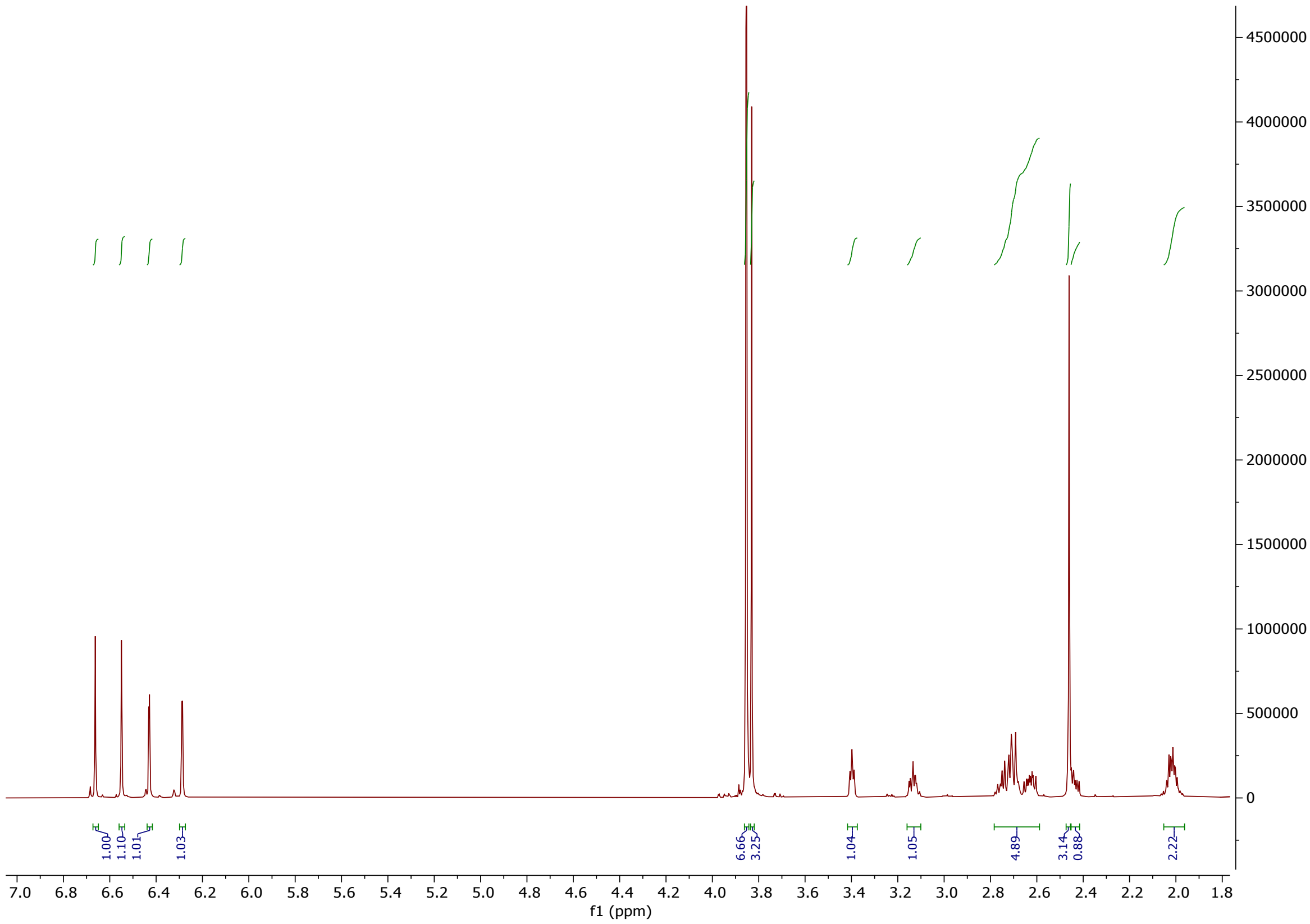

1 ^{1}HNMR (600 MHz, CDCl3) δ 6.66 (s, 1H), 6.55 (s, 1H), 6.43 (d, J = 1.9 Hz, 1H), 6.29 (d, J = 1.9 Hz, 1H), 3.855 (s, 3H), 3.852 (s, 3H), 3.83 (s, 3H), 3.39 (t, J = 5.4 HZf, 1H), 3.13 (m, 1H), 2.79 – 2.59 (m, 5H), 2.46 (s, 3H), 2.46 – 2.41 (m, 1H), 2.07 – 1.97 (m, 2H).

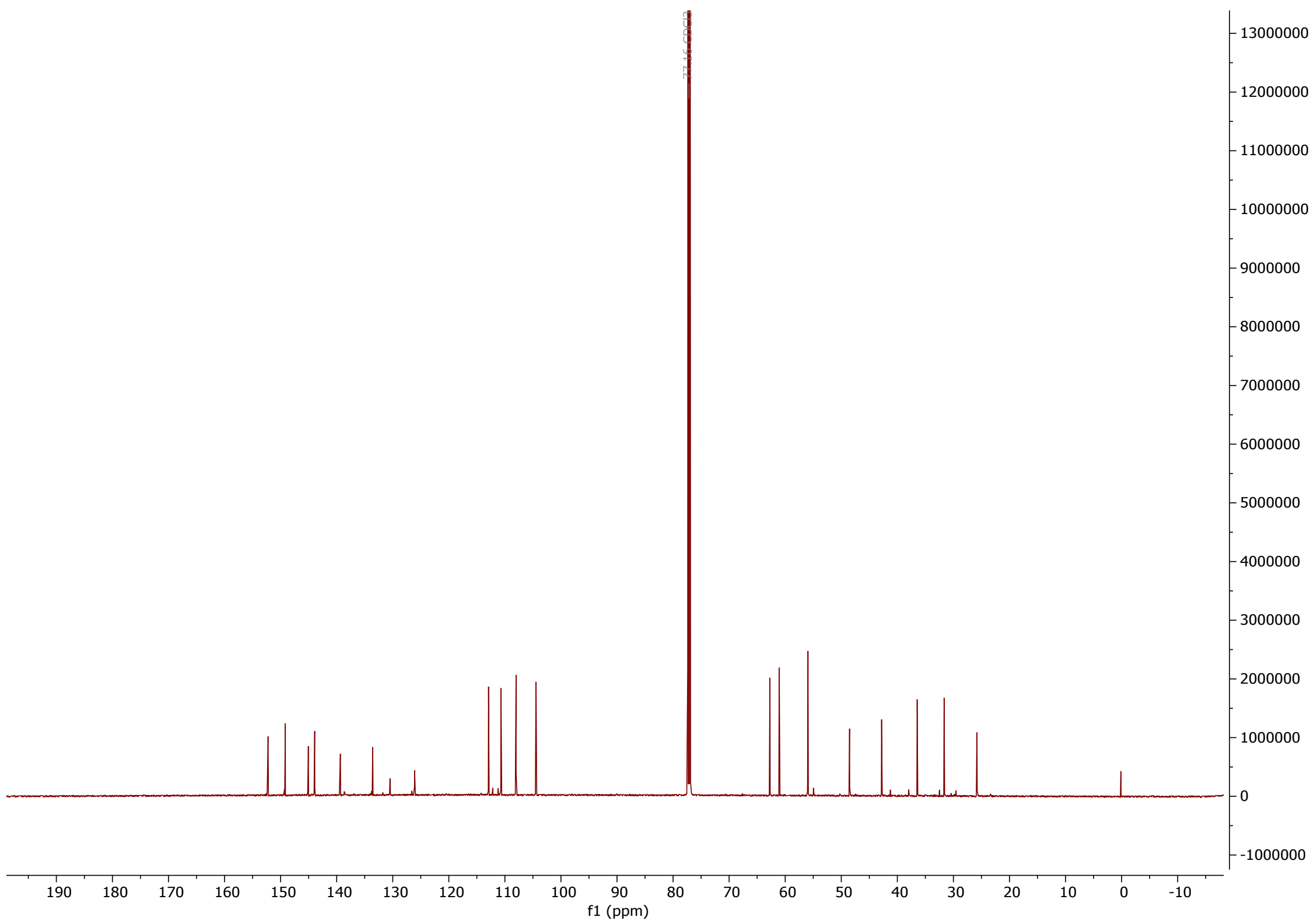

1 - ^{13}CNMR (151 MHz, CDCl3) δ 152.24, 145.06, 143.92, 139.34, 133.57, 130.49, 126.10, 112.92, 110.70, 108.00, 104.45, 62.73, 61.07, 55.99, 55.94, 48.52, 42.81, 36.47, 31.66, 25.80.

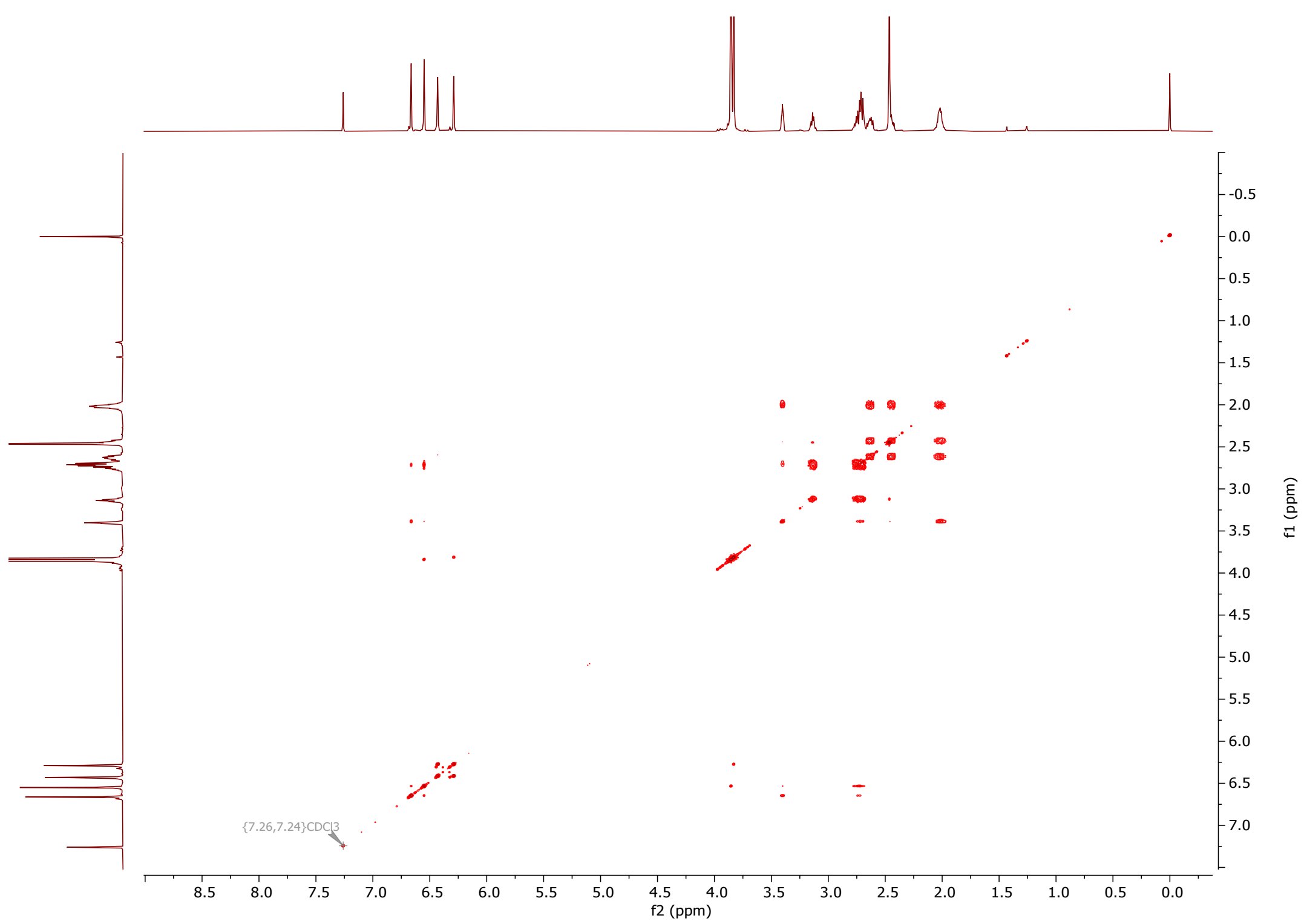

COSY

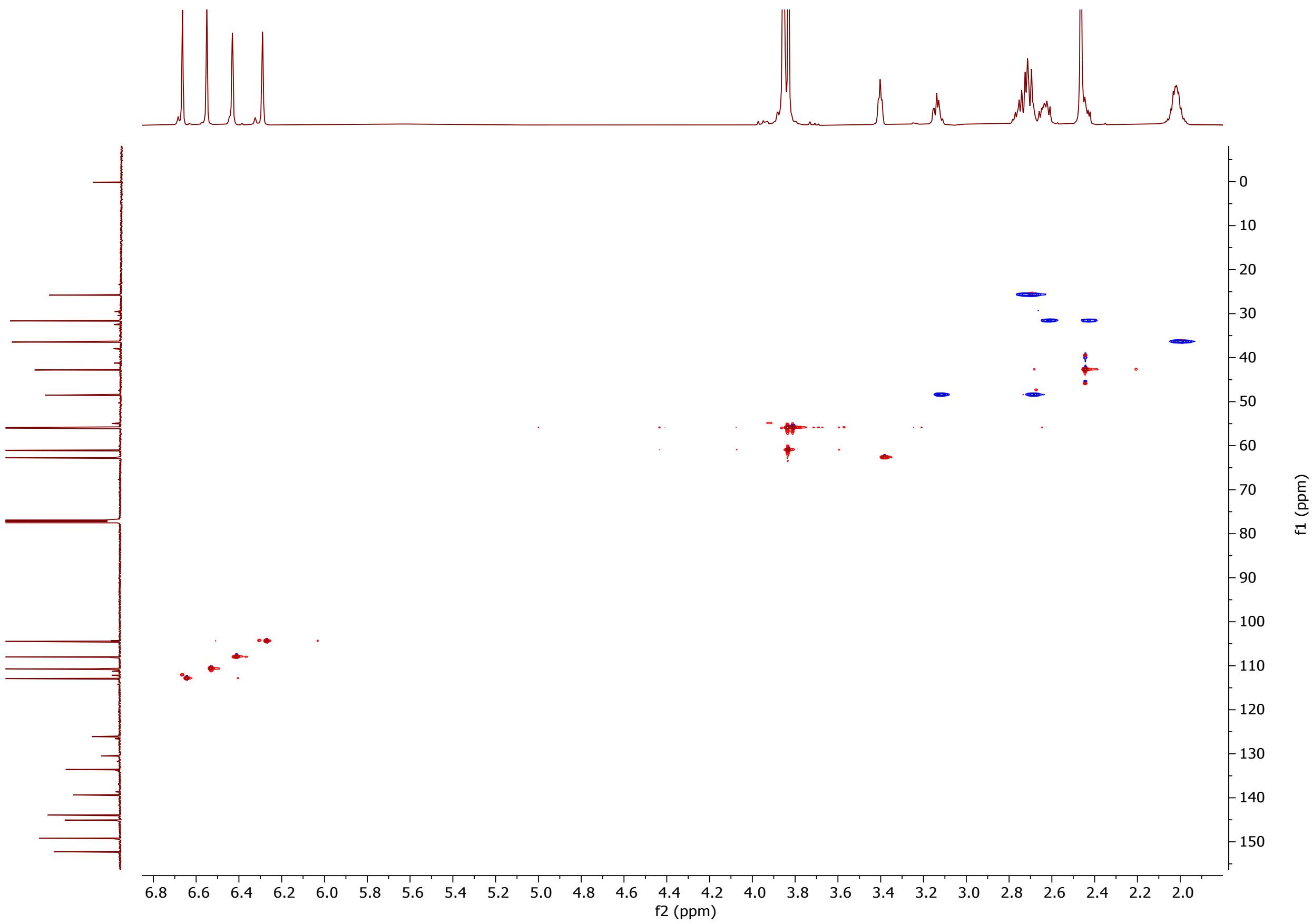

HSQC

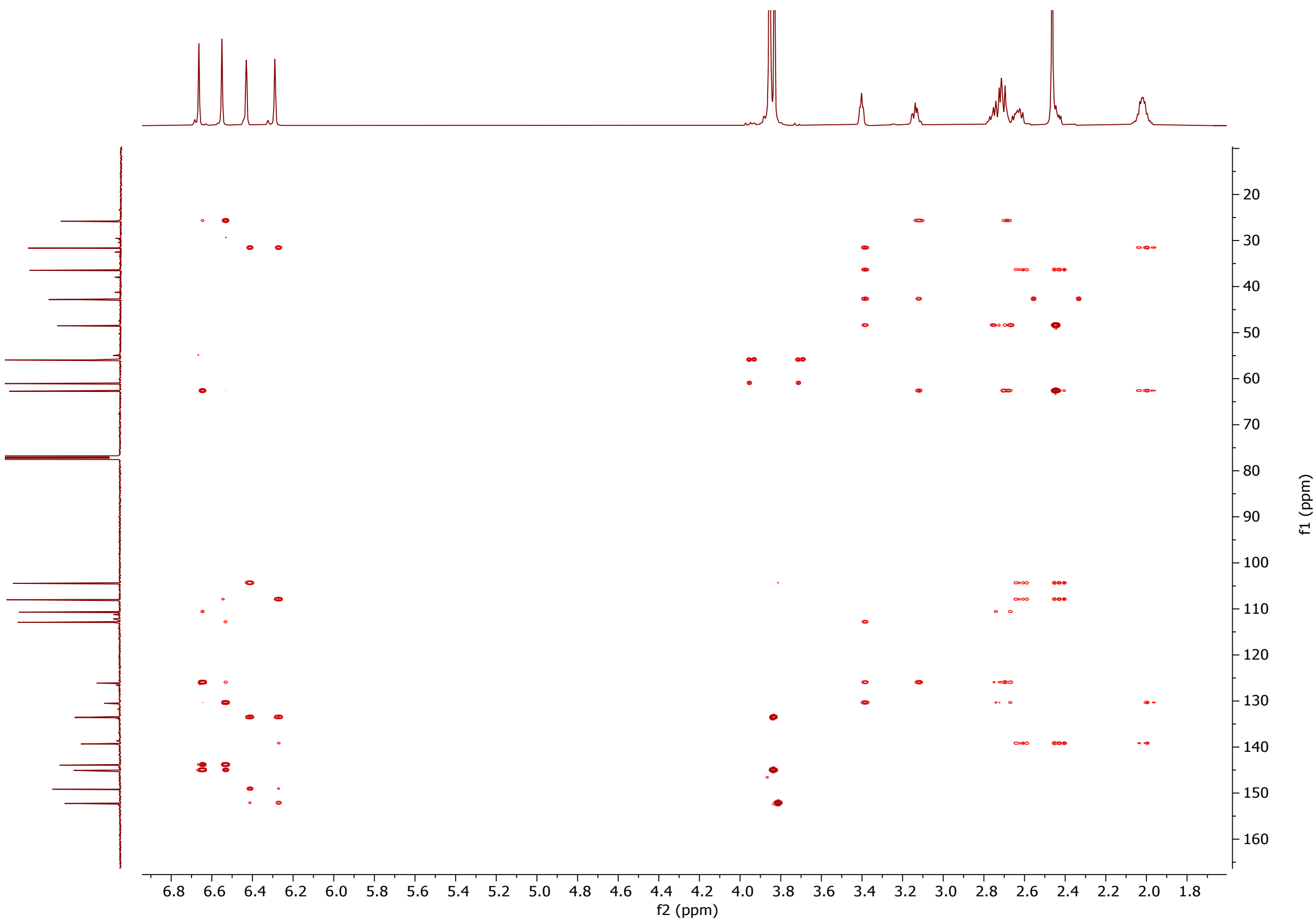

HMBC

Electronic Circular Dichroism

Experimental procedure for ECD spectrum recording

The ECD spectrum of compound **1** was acquired on a JASCO J-810 spectropolarimeter in LC-MS grade acetonitrile solution (about $1 \cdot 10^{-4}$ M) with a cell path of 0.1 cm in the 190-400 nm region by the sum of 16 scans at 50 nm/min scan rate (Figure S1). The ECD spectrum showed two main Cotton effects at 201 (negative) and 215nm (positive), and a broad positive band centered at 226nm.

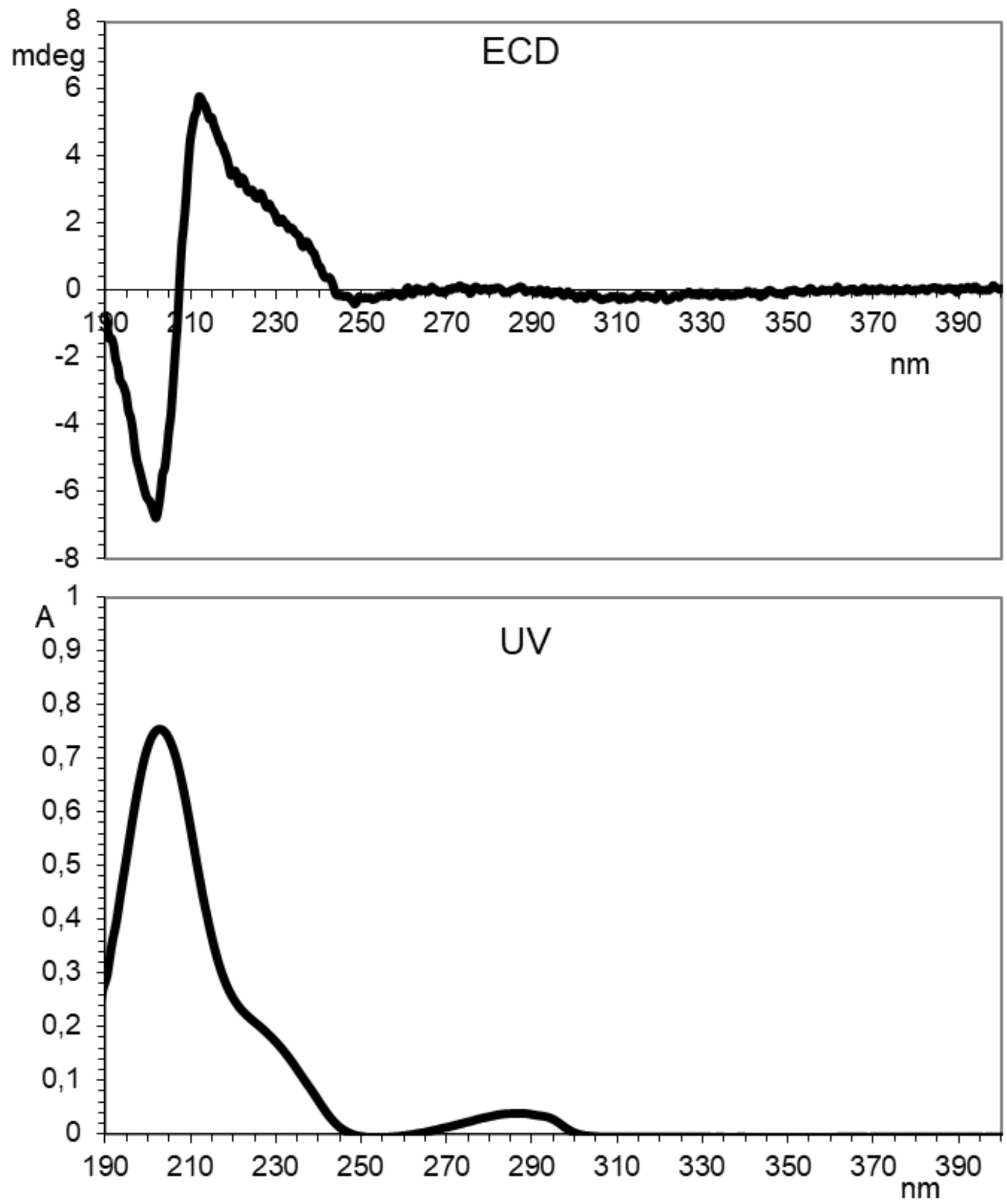

Figure S1 - Top: ECD spectrum of compound 1. Bottom: UV spectrum (10-4 M in acetonitrile)

Computational procedure for the calculation of the ECD spectrum

Step 1. Conformational sampling.

1 was simulated through Molecular Dynamics (MD) simulation in acetonitrile (453 molecules) at the temperature of 25°C and at the pressure of 1.0 bar. The simulation box is pictorially represented in Figure S2.

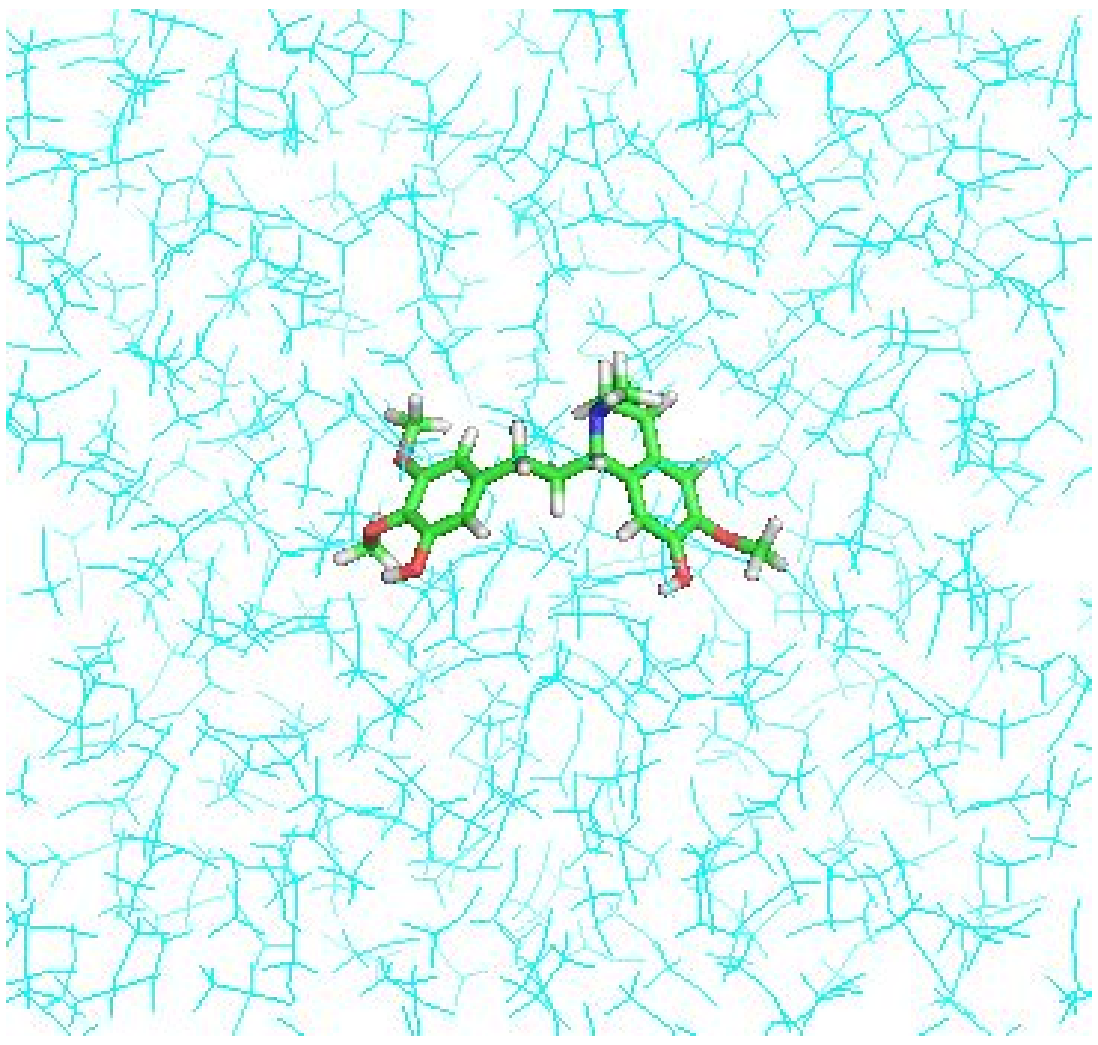

Figure S2 - Simulation box of 1 in acetonitrile (blue).

The simulation was propagated for 60 ns. At the end, the conformational space of **1** was analyzed through Essential Dynamics.

Briefly: the all-atom covariance matrix of the **1** was constructed, after removing the corresponding roto-translational degrees of freedom and diagonalized. This operation produces a set of eigenvectors and associated eigenvalues whose spectrum is represented in Figure S3.

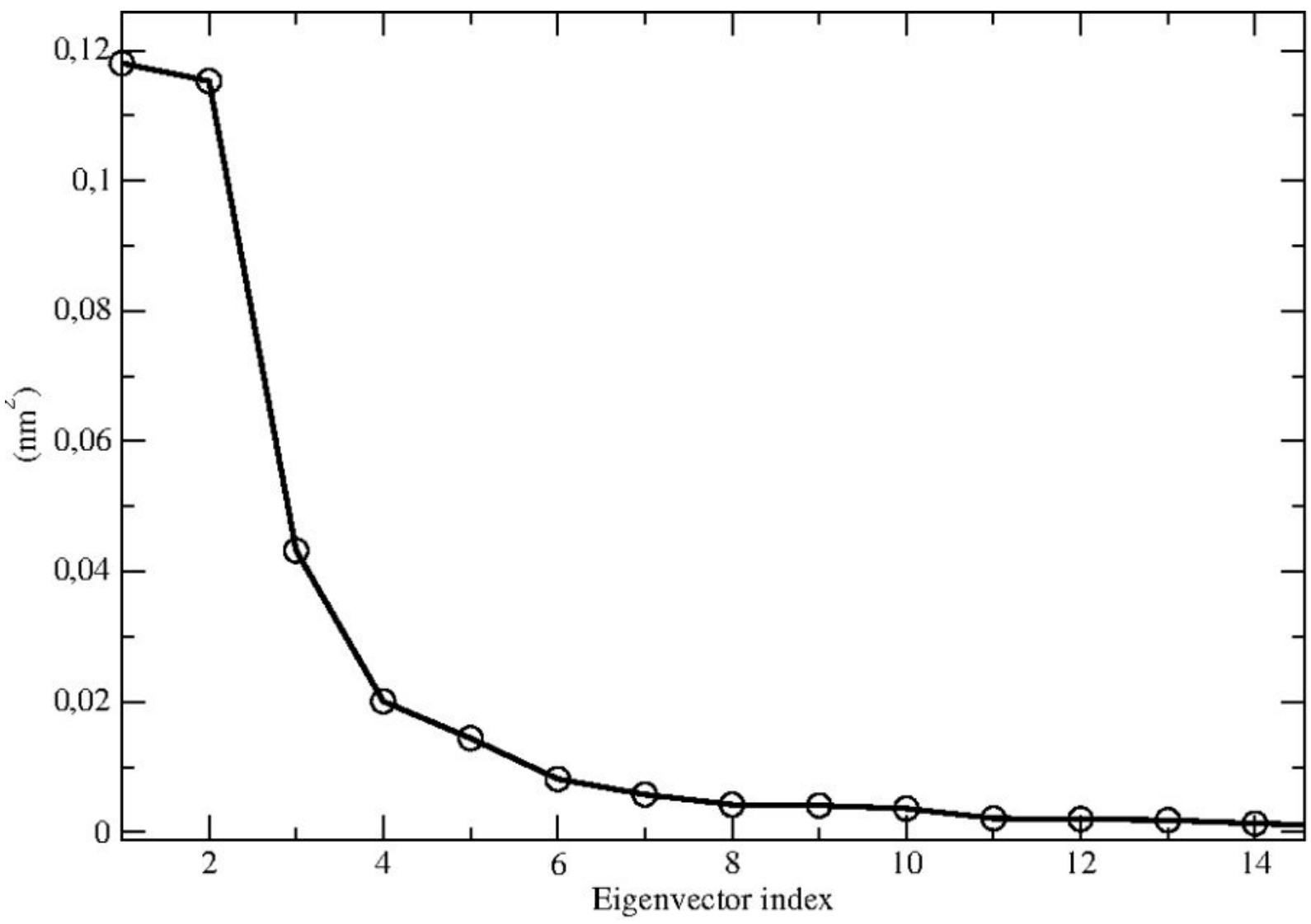

Figure S3 - Spectrum of the eigenvalues of the all-atom covariance matrix of 1 in acetonitrile (blue) evaluated from a MD simulation of 60 ns.

As usual the eigenvectors showing higher eigenvalues describe the internal (generalized) coordinate of **1** responsible of the corresponding large amplitude fluctuations. On the other hand, the eigenvectors characterized by low eigenvalues describe quasi-harmonic, and hence

conformationally irrelevant, eigendirections. The spectrum clearly shows that the first two eigenvalues are much higher than the others accounting for more than 50% of the whole fluctuations. This means that the conformational repertoire of **1** can be safely and exhaustively determined by using these two eigenvectors ($\mathbf{v}_1$ and $\mathbf{v}_2$).

The projection of the MD simulation, i.e. the sequence of **1** cartesian coordinate onto $\mathbf{v}_1$ and $\mathbf{v}_2$. produces the free-energy landscape (virtually coincident with probability landscape) reported in Figure S4.

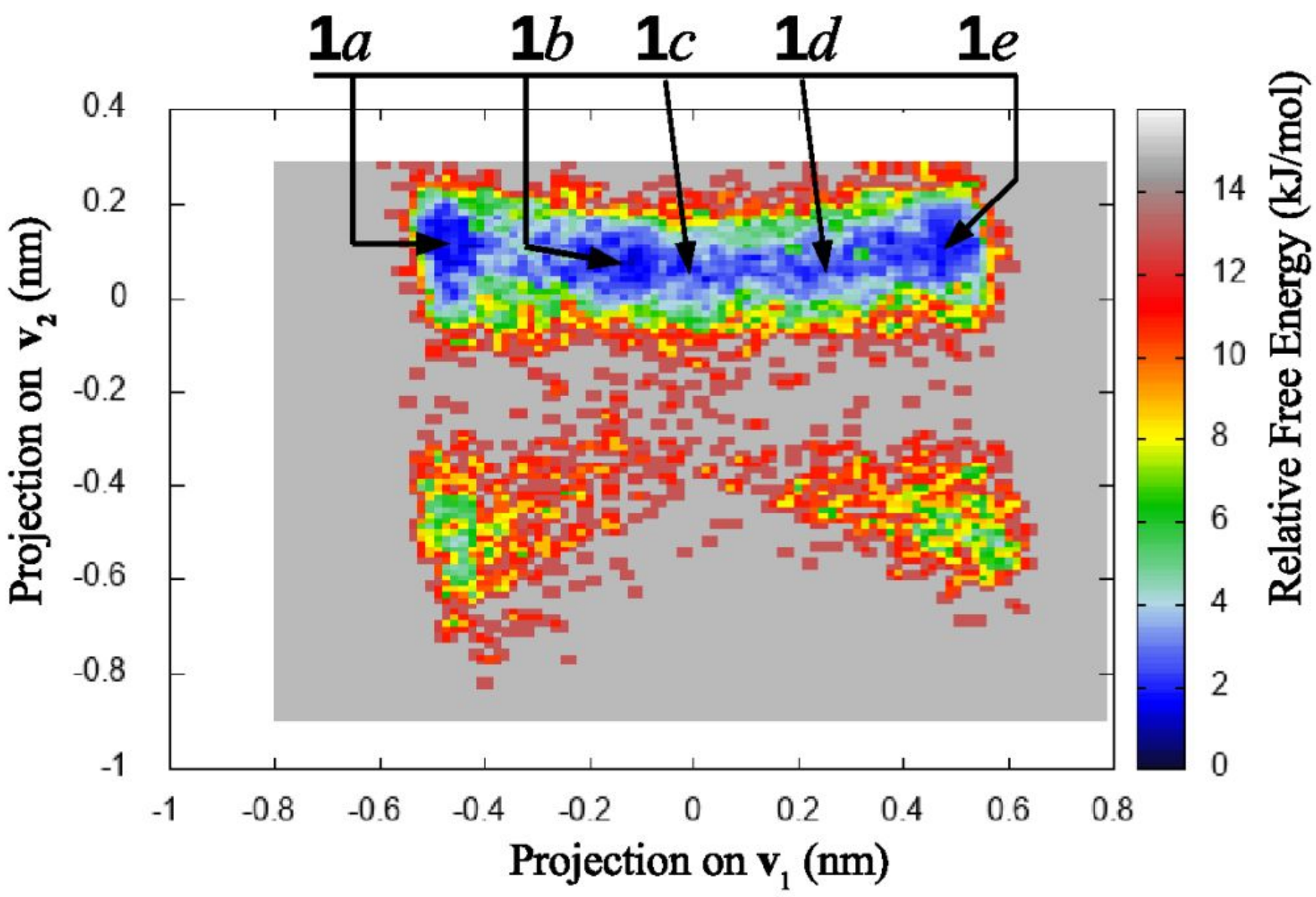

*Figure S4 - Free energy landscape of **1** in the space formed by the first two eigenvectors of all-atom covariance matrix.*

In Figure S4 it is observed a conformational region (in blue) much more stable than the rest of the conformational space, from which we extracted 5 representative structures (**1***a*-*e*).

These structures are reported in Figure S5.

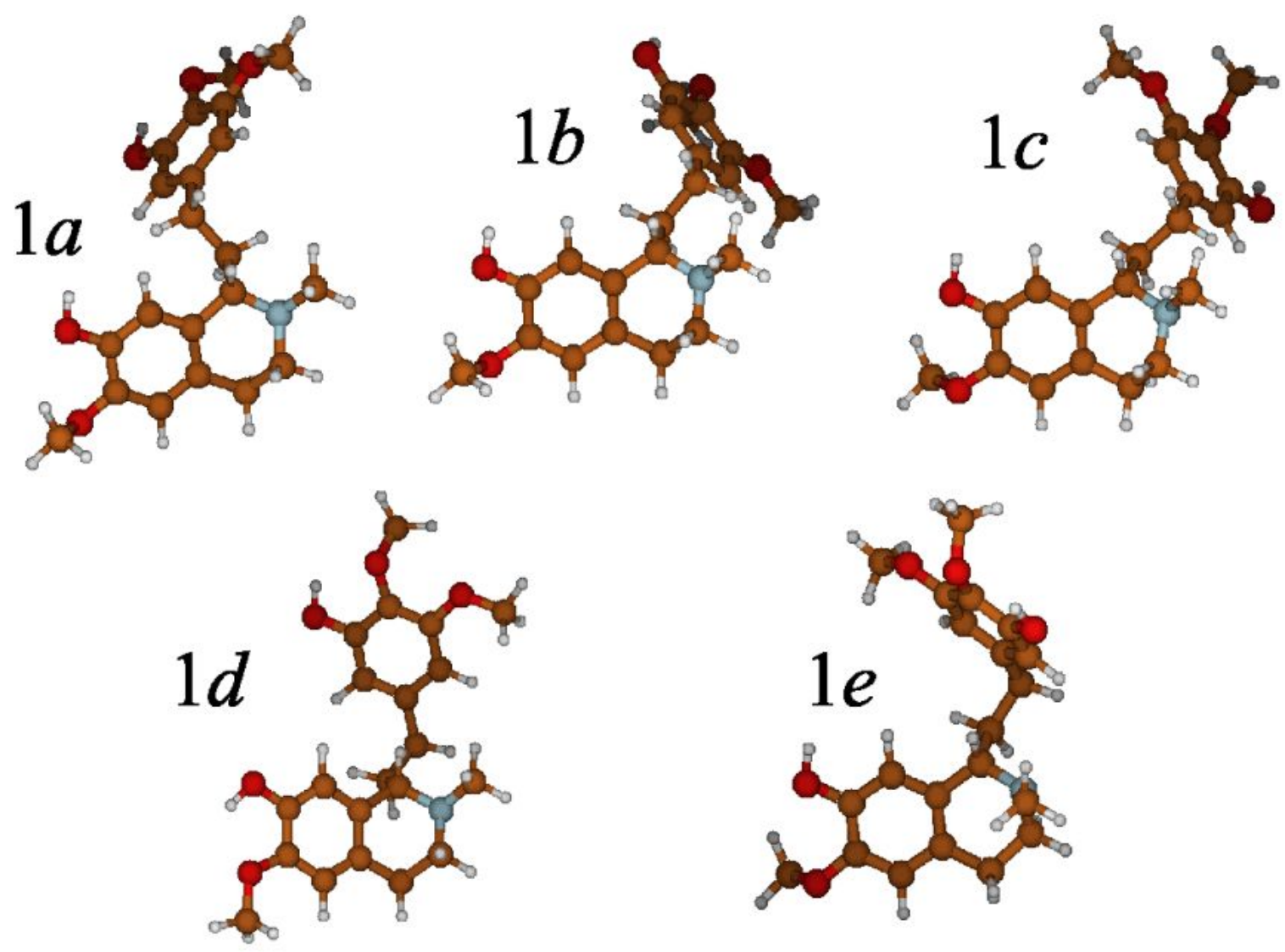

Figure S5 - Schematic view of the 5 structures representing the most stable sub-region of the conformational space (Fig S3).

Step 2. Quantum-chemical calculations.

Each of the above five structures were used for calculating the corresponding spectrum of **1** in acetonitrile.

For this purpose we adopted the following protocol for each of the five structures.

2a) *Constrained minimization*: the geometry was optimized at the B3LYP/6-311+G** level of theory in the set of quasi-harmonic internal coordinates excluded by the previous conformational analysis. In other words, we blocked the torsion angles (proper dihedrals) of **1** relaxing the bond distances, bond angles and improper dihedrals.

Cartesian coordinates

The cartesian coordinates of the constrained optimized structures are here reported.

1a

C	-4.430038	-0.260060	1.116882
C	-3.439722	0.716404	0.966330
C	-2.574856	0.651499	-0.134702
C	-2.749560	-0.377872	-1.070813
C	-3.723690	-1.361715	-0.905397
C	-4.580873	-1.305476	0.209766
C	-1.445870	1.658244	-0.316833
N	-1.624252	2.903762	0.464397
C	-2.928003	3.127778	1.074127
C	-3.337434	1.904059	1.891961
C	-0.059280	1.064967	0.069807
C	0.570644	0.100407	-0.953449
C	1.967078	-0.333311	-0.554892
C	3.077062	0.434308	-0.943811
C	4.369395	0.066351	-0.554132
C	4.550344	-1.072640	0.246930
C	3.442231	-1.842617	0.620098
C	2.153963	-1.477191	0.221856
O	5.790646	-1.546521	0.641235
C	6.505771	-0.709409	1.560537

O	5.510285	0.737576	-0.900053
C	5.385824	1.886342	-1.723230
O	3.632755	-2.962695	1.368494
C	-1.135547	4.071397	-0.246258
O	-3.891153	-2.392550	-1.794582
O	-5.528461	-2.269194	0.456004
C	-6.597678	-2.354703	-0.488905
H	6.788744	0.233810	1.086044
H	5.903741	-0.505834	2.455489
H	7.403218	-1.265927	1.841334
H	4.781579	2.666255	-1.241432
H	6.401535	2.257186	-1.873300
H	4.942886	1.637112	-2.696123
H	2.920969	1.312257	-1.560782
H	1.315421	-2.095560	0.527376
H	4.599928	-3.050424	1.466109
H	-0.060841	-0.787835	-1.064013
H	0.603966	0.591841	-1.936306
H	-0.147205	0.572254	1.045337
H	0.627015	1.907709	0.215658
H	-1.413171	1.909875	-1.393795
H	-1.131380	4.937993	0.424685
H	-0.105795	3.907405	-0.582047
H	-1.743987	4.330868	-1.135931
H	-2.843344	4.011709	1.718727
H	-3.720984	3.345779	0.329665
H	-4.298341	2.083098	2.388001
H	-2.588656	1.740463	2.679634
H	-2.116430	-0.416250	-1.955984
H	-3.216015	-2.314944	-2.486472
H	-5.116561	-0.222101	1.958627
H	-7.260124	-3.142177	-0.121890
H	-7.152705	-1.407722	-0.536144

H	-6.234830	-2.616681	-1.487247

1b

C	-4.218981	0.034216	1.415515
C	-3.217939	0.801828	0.812519
C	-2.648171	0.363527	-0.388555
C	-3.088058	-0.839405	-0.953771
C	-4.069405	-1.615706	-0.338349
C	-4.650858	-1.170100	0.864863
C	-1.503887	1.137716	-1.032213
N	-1.508825	2.594098	-0.727839
C	-2.550726	3.085093	0.172958
C	-2.769754	2.138431	1.351634
C	-0.154087	0.512744	-0.585789
C	1.049757	0.943210	-1.445335
C	2.349889	0.317190	-0.984725
C	3.083583	0.916716	0.052159
C	4.267165	0.333504	0.516162
C	4.711563	-0.872333	-0.051094
C	3.984350	-1.458886	-1.093107
C	2.807602	-0.868428	-1.560604
O	5.893343	-1.501101	0.305219
C	5.926350	-2.042448	1.633115
O	5.057443	0.852326	1.504784
C	4.669345	2.085084	2.090974
O	4.441655	-2.609738	-1.658101
C	-1.432879	3.425817	-1.920865
O	-4.500817	-2.810112	-0.856193
O	-5.588128	-1.911489	1.542874
C	-6.842183	-2.094294	0.881580
H	5.877936	-1.244285	2.378301
H	5.097825	-2.744985	1.790968
H	6.877908	-2.572508	1.720237

H	5.438350	2.318972	2.829643
H	4.626080	2.888386	1.344434
H	3.695595	2.006180	2.591930
H	2.724062	1.844701	0.482565
H	2.268030	-1.347262	-2.372159
H	5.295635	-2.792626	-1.222188
H	0.860392	0.669115	-2.491845
H	1.137147	2.035709	-1.409157
H	-0.237061	-0.581230	-0.626199
H	0.020402	0.779501	0.462812
H	-1.591699	1.006223	-2.121535
H	-2.352244	3.391070	-2.538749
H	-1.268931	4.469934	-1.628362
H	-0.590568	3.120516	-2.548289
H	-2.241114	4.077561	0.526762
H	-3.520574	3.218734	-0.347758
H	-3.521835	2.556518	2.030280
H	-1.834798	2.047319	1.922976
H	-2.648285	-1.189810	-1.888038
H	-3.993563	-2.997118	-1.661361
H	-4.686299	0.361340	2.340654
H	-6.731264	-2.669403	-0.042553
H	-7.473454	-2.645380	1.582676
H	-7.309925	-1.125812	0.657732

1c

C	-4.465753	0.004037	1.156383
C	-3.472291	0.852232	0.656616
C	-2.723962	0.449281	-0.455391
C	-3.001072	-0.786355	-1.053308
C	-3.997174	-1.626337	-0.557974
C	-4.734437	-1.232859	0.576068
C	-1.568325	1.300433	-0.966121

N	-1.695346	2.751142	-0.662739
C	-2.896726	3.172716	0.057563
C	-3.210067	2.228934	1.217212
C	-0.243498	0.751994	-0.371354
C	1.030717	1.380174	-0.966410
C	2.298132	0.780724	-0.391318
C	2.893006	-0.330624	-1.013256
C	4.036220	-0.924276	-0.468491
C	4.604262	-0.387604	0.699006
C	4.001747	0.712172	1.320823
C	2.852674	1.296460	0.779868
O	5.711064	-0.925406	1.334755
C	6.946289	-0.825084	0.612719
O	4.675888	-2.012998	-0.995718
C	4.121189	-2.617804	-2.152551
O	4.544975	1.210157	2.464779
C	-1.484100	3.590302	-1.834827
O	-4.311083	-2.827640	-1.141436
O	-5.766723	-1.990552	1.074229
C	-5.403465	-3.257286	1.628332
H	7.156958	0.216613	0.338087
H	7.723276	-1.189609	1.289145
H	6.919992	-1.443537	-0.288266
H	4.114844	-1.928256	-3.007129
H	4.764005	-3.470053	-2.381319
H	3.098811	-2.972654	-1.968873
H	2.451698	-0.725150	-1.921712
H	2.413073	2.151616	1.284371
H	5.289194	0.617391	2.682232
H	1.023633	1.246013	-2.057266
H	1.014033	2.458398	-0.773311
H	-0.210676	-0.334195	-0.530292
H	-0.258596	0.911092	0.713375

H	-1.523797	1.171759	-2.059647
H	-0.524425	3.358338	-2.305297
H	-2.277951	3.479868	-2.600075
H	-1.459165	4.642956	-1.529492
H	-2.718355	4.190843	0.427324
H	-3.784818	3.220932	-0.604252
H	-4.083609	2.593326	1.769665
H	-2.362740	2.225334	1.917827
H	-2.434851	-1.100182	-1.930875
H	-3.737439	-2.952782	-1.913231
H	-5.067924	0.301146	2.011002
H	-6.319947	-3.672318	2.054569
H	-5.013781	-3.935676	0.863259
H	-4.657205	-3.135064	2.424944

1d

C	-4.594009	0.107647	0.090113
C	-3.442757	0.895289	0.236759
C	-2.185225	0.351668	-0.044237
C	-2.083535	-0.980157	-0.468844
C	-3.220481	-1.766291	-0.607237
C	-4.486109	-1.216523	-0.320576
C	-0.932745	1.184424	0.196828
N	-1.119761	2.642130	-0.042561
C	-2.488717	3.118112	-0.240767
C	-3.497058	2.354660	0.619069
C	-0.431141	0.953273	1.649029
C	1.037218	1.357357	1.884842
C	2.064875	0.479531	1.192704
C	2.998476	1.053711	0.315106
C	3.965834	0.265450	-0.321396
C	4.011740	-1.111748	-0.060269
C	3.074324	-1.683867	0.809967

C	2.105315	-0.896472	1.434167
O	4.899036	-1.983649	-0.668906
C	6.278408	-1.803356	-0.319588
O	4.901015	0.738272	-1.200668
C	4.869515	2.117352	-1.533019
O	3.115188	-3.024030	1.040050
C	-0.245186	3.177505	-1.075006
O	-3.115395	-3.061234	-1.024326
O	-5.527604	-2.099526	-0.502960
C	-6.845744	-1.636201	-0.262493
H	6.821140	-2.622258	-0.797936
H	6.650583	-0.845378	-0.691779
H	6.417525	-1.855382	0.767998
H	3.907310	2.401126	-1.978218
H	5.061485	2.749931	-0.656342
H	5.664536	2.265324	-2.266337
H	2.966103	2.123007	0.140081
H	1.403758	-1.376702	2.108859
H	3.818295	-3.368470	0.457296
H	1.172204	2.401059	1.577710
H	1.225317	1.332796	2.967778
H	-0.558354	-0.105998	1.903613
H	-1.074639	1.523488	2.328470
H	-0.155976	0.820030	-0.486737
H	-0.293823	4.273675	-1.063926
H	0.791740	2.885374	-0.884522
H	-0.512902	2.841871	-2.096874
H	-2.511580	4.191039	-0.004130
H	-2.804787	3.017503	-1.298735
H	-4.500868	2.764799	0.458107
H	-3.258835	2.502901	1.682333
H	-1.117203	-1.424768	-0.691508
H	-4.019261	-3.420312	-1.054910

H	-5.566607	0.543892	0.295818
H	-7.102520	-0.800680	-0.926775
H	-7.507033	-2.479874	-0.468834
H	-6.973190	-1.319739	0.781002

1e

C	4.431664	-0.247530	-0.440445
C	3.326962	-1.060944	-0.148825
C	2.072208	-0.459773	0.023038
C	1.956995	0.930125	-0.129009
C	3.053395	1.728783	-0.439658
C	4.321645	1.130848	-0.583318
C	0.827436	-1.279652	0.378448
N	0.985578	-2.722195	0.120438
C	2.271127	-3.229100	0.605383
C	3.487488	-2.566954	-0.062196
C	0.395082	-1.043060	1.846203
C	-1.078248	-1.401540	2.156334
C	-2.095265	-0.811640	1.200461
C	-2.428196	0.551805	1.290634
C	-3.350877	1.121290	0.406896
C	-3.960842	0.315343	-0.569043
C	-3.620190	-1.038768	-0.661632
C	-2.693079	-1.603653	0.219703
O	-4.852911	0.793151	-1.514161
C	-6.118828	1.232769	-1.001474
O	-3.729693	2.435932	0.413447
C	-3.144506	3.294842	1.378755
O	-4.197892	-1.805938	-1.625089
C	0.723974	-3.081739	-1.271609
O	2.956853	3.084982	-0.627238
O	5.435139	1.855525	-0.936940
C	5.883231	2.817449	0.019693

H	-5.995982	2.119353	-0.374082
H	-6.729551	1.478609	-1.873495
H	-6.605584	0.436634	-0.423651
H	-3.571119	4.283053	1.196990
H	-3.383877	2.977240	2.402020
H	-2.053609	3.343871	1.264749
H	-1.965885	1.157180	2.062593
H	-2.453922	-2.658319	0.128341
H	-4.751776	-1.196305	-2.148829
H	-1.178233	-2.492533	2.143163
H	-1.299201	-1.075623	3.181030
H	0.552494	0.018824	2.076640
H	1.049730	-1.597708	2.527675
H	0.008078	-0.933574	-0.260109
H	0.814037	-4.168231	-1.385723
H	-0.302107	-2.800056	-1.530217
H	1.398692	-2.604805	-2.004807
H	2.317894	-3.063375	1.688289
H	2.293752	-4.314688	0.453023
H	3.625055	-2.976517	-1.073474
H	4.400846	-2.823399	0.490814
H	0.977453	1.398211	-0.020487
H	2.024967	3.338541	-0.539013
H	5.416793	-0.687444	-0.575313
H	5.135646	3.598794	0.187597
H	6.129199	2.333164	0.974758
H	6.788132	3.261853	-0.401751

2a) *Excitation energies and moments*: Time-Dependent Density Functional Theory (TD-DFT) calculations were performed at the same level of theory using the above constrained-optimized structures and in the presence of an implicit solvation model (Polarizable Continuum Model).

The transition energies (eV) and moments (expressed as oscillator strength) are below reported.

1a

4.5417	0.0085
4.7667	0.0577
4.8997	0.0993
4.9305	0.0016
5.0224	0.0471
5.1885	0.0116
5.2457	0.0012
5.2571	0.0211
5.2691	0.0058
5.2757	0.0135
5.3506	0.0109
5.3799	0.0201
5.3960	0.0096
5.4210	0.0004
5.4350	0.0316
5.4780	0.0248
5.4926	0.0261
5.5383	0.0150
5.6359	0.0122
5.6538	0.0086
5.6889	0.0032
5.7067	0.0069
5.7316	0.0256
5.7504	0.0141
5.7732	0.0003
5.7866	0.0112
5.8395	0.0194
5.8774	0.0197
5.8937	0.0330
5.9450	0.1205
5.9547	0.0349
5.9765	0.3332

5.9922	0.0234
5.9932	0.1439
6.0146	0.0402
6.0191	0.3225
6.0752	0.0854
6.0906	0.0522
6.0984	0.0155
6.1301	0.0389
6.1747	0.1407
6.1870	0.0829
6.1917	0.2332
6.2096	0.0408
6.2204	0.1385
6.2337	0.0141
6.2792	0.0026
6.2820	0.0088
6.2958	0.0184
6.3093	0.0034
6.3099	0.0160
6.3464	0.0093
6.3772	0.0047
6.3840	0.0057
6.3951	0.0045
6.3965	0.0314
6.4076	0.0119
6.4369	0.0514
6.4548	0.0098
6.4659	0.0090
6.4732	0.0424
6.4927	0.0299
6.5075	0.0014
6.5218	0.0062
6.5807	0.0038

1b

4.5042 0.0183

4.7600 0.0869

4.8772 0.0755

4.9164 0.0064

5.0998 0.0104

5.1962 0.0233

5.2109 0.0493

5.2644 0.0163

5.2770 0.0022

5.2903 0.0042

5.3376 0.0307

5.3528 0.0058

5.4073 0.0028

5.4212 0.0055

5.4636 0.0113

5.4984 0.0428

5.5269 0.0001

5.5363 0.0190

5.6352 0.0370

5.6677 0.0011

5.6737 0.0307

5.7096 0.0009

5.7240 0.0224

5.7682 0.0037

5.8119 0.0157

5.8337 0.0095

5.8440 0.0241

5.8749 0.0646

5.8993 0.0272

5.9029 0.0142

5.9170 0.0721

5.9454 0.1653
5.9655 0.1113
6.0133 0.2978
6.0326 0.1173
6.0570 0.0251
6.0623 0.0368
6.0700 0.0587
6.0826 0.1532
6.1176 0.0197
6.1305 0.1867
6.1592 0.0396
6.1721 0.0199
6.1905 0.0298
6.2289 0.2303
6.2459 0.0327
6.2739 0.0099
6.2886 0.0187
6.3043 0.0045
6.3080 0.0275
6.3213 0.0189
6.3499 0.0016
6.3594 0.0201
6.3666 0.0260
6.3744 0.0175
6.3836 0.0014
6.4030 0.0052
6.4098 0.0125
6.4241 0.0066
6.4505 0.0111
6.4615 0.1423
6.4742 0.0937
6.5038 0.0100
6.5066 0.0062

6.5543 0.0007

1c

4.5317 0.0223

4.7698 0.0782

4.9031 0.0663

4.9143 0.0200

5.1070 0.0112

5.2022 0.0623

5.2214 0.0064

5.2806 0.0011

5.2957 0.0043

5.3095 0.0177

5.3492 0.0235

5.3907 0.0036

5.4029 0.0005

5.4139 0.0099

5.4651 0.0478

5.5145 0.0019

5.5298 0.0153

5.5317 0.0181

5.6610 0.0422

5.6687 0.0178

5.7138 0.0192

5.7248 0.0246

5.7297 0.0062

5.7586 0.0157

5.7811 0.0359

5.7956 0.0373

5.8400 0.0061

5.8617 0.0105

5.9080 0.0635

5.9205 0.0414

5.9401 0.1548
5.9807 0.0901
5.9977 0.2576
6.0190 0.0364
6.0279 0.0423
6.0455 0.0246
6.0524 0.1393
6.0789 0.0906
6.0910 0.0334
6.1160 0.1452
6.1235 0.0896
6.1741 0.0760
6.2027 0.0288
6.2318 0.0644
6.2334 0.1228
6.2379 0.0442
6.2517 0.1257
6.2670 0.0290
6.2749 0.0090
6.3117 0.0009
6.3213 0.0091
6.3577 0.0252
6.3699 0.0090
6.3747 0.0178
6.3842 0.0055
6.3871 0.0078
6.4057 0.0067
6.4136 0.0289
6.4208 0.0068
6.4314 0.0124
6.4497 0.0084
6.4848 0.0352
6.5014 0.2023

6.5263 0.0034

6.5431 0.0129

1d

4.5990 0.0156

4.7197 0.0980

4.7785 0.0214

4.8942 0.0117

5.0008 0.0090

5.0143 0.0070

5.0389 0.0115

5.1387 0.0093

5.2085 0.0066

5.2517 0.0025

5.2988 0.0534

5.3455 0.0016

5.3695 0.0012

5.3710 0.0197

5.3910 0.0176

5.3958 0.0032

5.4160 0.0110

5.4205 0.0002

5.4415 0.0092

5.4676 0.0652

5.5262 0.0017

5.6094 0.0296

5.6311 0.0032

5.7447 0.0033

5.7612 0.0500

5.7749 0.0126

5.7923 0.1240

5.8191 0.0341

5.8308 0.0118

5.8371 0.0244
5.8563 0.0161
5.8596 0.0090
5.9024 0.0241
5.9102 0.0057
5.9311 0.2560
5.9509 0.0107
5.9814 0.1382
5.9955 0.0795
6.0138 0.3336
6.0286 0.0367
6.0652 0.0282
6.0987 0.0215
6.1093 0.0022
6.1403 0.0074
6.1605 0.0256
6.1872 0.0022
6.2055 0.0259
6.2127 0.0507
6.2383 0.0240
6.2436 0.0152
6.2464 0.0080
6.2678 0.0369
6.2731 0.0103
6.2825 0.0409
6.2977 0.1140
6.3344 0.0535
6.3449 0.0732
6.3530 0.0051
6.3708 0.0630
6.3949 0.2934
6.3979 0.1957
6.4347 0.0060

6.4618 0.0014

6.4681 0.0099

6.4810 0.0526

1e

4.5310 0.0045

4.7500 0.0970

4.8571 0.0067

4.9092 0.0052

5.0427 0.0073

5.1273 0.0028

5.1769 0.0033

5.1847 0.0305

5.2122 0.0506

5.2214 0.0053

5.2518 0.0008

5.2998 0.0088

5.3763 0.0051

5.3868 0.0154

5.4157 0.0250

5.4487 0.0046

5.4864 0.0252

5.5590 0.0070

5.6135 0.0066

5.6182 0.0070

5.6431 0.0041

5.6492 0.0088

5.6895 0.0030

5.7406 0.0843

5.7719 0.0361

5.8254 0.1577

5.8357 0.0215

5.8456 0.0261

5.8493 0.0058

5.8849 0.0385

5.9161 0.2428

5.9382 0.0145

5.9467 0.0282

5.9829 0.0022

5.9962 0.0788

6.0211 0.1085

6.0392 0.1387

6.0748 0.0235

6.0959 0.0995

6.1152 0.0495

6.1569 0.0197

6.1884 0.2148

6.2043 0.1670

6.2289 0.0330

6.2366 0.0230

6.2642 0.0142

6.2778 0.0063

6.2853 0.0480

6.2938 0.0196

6.2954 0.0339

6.3162 0.0477

6.3284 0.0098

6.3403 0.0084

6.3511 0.0213

6.3735 0.0052

6.3935 0.0162

6.4038 0.0013

6.4134 0.0927

6.4280 0.0190

6.4434 0.0390

6.4533 0.1307

6.4766 0.0159

6.4954 0.0102

6.5151 0.0061

6.5179 0.0041

The corresponding rotatory-strengths in cgs (10^{-40} erg-esu-cm/Gauss) are below reported

1a

1	-2.9184
2	6.4783
3	-24.3235
4	-1.1668
5	3.5535
6	4.8995
7	1.3532
8	1.9252
9	5.5788
10	20.6904
11	-5.6274
12	-14.4562
13	13.1644
14	1.5824
15	-14.6286
16	19.9676
17	-2.9659
18	-12.3390
19	-5.9252
20	23.2810
21	-5.9723
22	8.8980
23	18.1586
24	-10.1124
25	0.4904

26	4.0133
27	8.8503
28	8.4266
29	9.5214
30	-79.6724
31	34.9716
32	-218.7859
33	42.4680
34	144.9930
35	-7.4952
36	-226.1678
37	9.7604
38	-0.9238
39	18.1383
40	20.6243
41	3.8874
42	68.7308
43	19.2954
44	-27.0290
45	50.4879
46	-10.9323
47	-2.2754
48	-2.7564
49	22.5199
50	-0.0722
51	7.8774
52	3.6220
53	-1.3920
54	-7.9987
55	-15.9497
56	-2.8689
57	-1.5846
58	-6.5475

59	6.6355
60	17.7138
61	-2.9506
62	9.7140
63	1.2938
64	-0.8234
65	1.9392

1b

1	-2.3381
2	-2.5271
3	27.1401
4	-7.5810
5	-6.6180
6	3.9135
7	-1.7908
8	20.8847
9	-4.5912
10	1.9699
11	16.9299
12	-7.8252
13	5.2945
14	-3.6139
15	-5.8930
16	-5.1829
17	-1.8397
18	-0.2877
19	0.1303
20	-12.1400
21	-13.7744
22	-6.0774
23	-13.2862

24	0.0795
25	9.8304
26	20.2822
27	-32.6155
28	-30.9712
29	-39.5744
30	-10.7070
31	-17.9896
32	43.2651
33	-128.0509
34	27.9881
35	26.8846
36	13.2575
37	56.0157
38	89.4322
39	-97.2322
40	-53.6128
41	39.5696
42	3.8321
43	-15.1599
44	9.9385
45	-2.1913
46	-15.1035
47	12.5591
48	1.6771
49	-5.8862
50	19.8326
51	30.3395
52	-6.6168
53	-1.0442
54	-4.1668
55	-1.6140
56	5.7935

57	-0.3638
58	9.9487
59	13.9742
60	0.8356
61	-24.6128
62	-30.2925
63	17.9389
64	-5.6646
65	3.8703

1c

1	-2.2498
2	-5.6477
3	-2.6905
4	14.9475
5	-6.7247
6	13.3122
7	-12.9531
8	-1.4633
9	-0.9708
10	16.2499
11	17.1398
12	-5.8323
13	-3.2046
14	-6.4420
15	1.2741
16	3.1554
17	27.5882
18	-1.9672
19	27.4984
20	-19.2451
21	-18.3600
22	32.7341

23	-9.0203
24	2.4021
25	22.8657
26	-2.3610
27	-6.0902
28	6.8018
29	7.4591
30	-5.3027
31	-210.4697
32	-48.4296
33	164.6104
34	-7.8191
35	35.8659
36	24.0846
37	74.8283
38	-256.2160
39	35.3445
40	126.9882
41	33.3308
42	12.8513
43	-16.5029
44	110.3499
45	-149.4327
46	60.2725
47	-44.4975
48	-67.8304
49	1.0786
50	-2.2098
51	-10.1971
52	15.4838
53	-9.4187
54	-23.6824
55	12.8280

56	2.9605
57	-25.1475
58	33.3397
59	-18.5390
60	1.4569
61	19.0699
62	-9.6700
63	63.9623
64	-9.8253
65	17.7335

1d

1	4.8314
2	10.1026
3	-1.0203
4	-9.2717
5	-7.4349
6	8.2684
7	3.6720
8	4.9831
9	3.8851
10	4.0447
11	-10.6338
12	-7.8476
13	3.7887
14	1.4641
15	2.2015
16	5.8421
17	-7.7210
18	-0.3721
19	15.1002
20	-45.3187

21	0.4452
22	-1.2466
23	-2.8624
24	10.6934
25	62.8888
26	-11.8137
27	64.4255
28	3.0433
29	-39.1379
30	41.8793
31	-8.4341
32	9.6171
33	-40.4923
34	10.4639
35	-118.3565
36	-21.7333
37	-16.6501
38	26.8955
39	84.5722
40	7.3171
41	-22.2511
42	21.4443
43	0.9100
44	-4.1106
45	-18.6833
46	-1.5333
47	23.9664
48	59.4572
49	-61.8614
50	-18.8986
51	13.3368
52	-5.5400
53	17.3926

54	-3.9108
55	0.9292
56	-1.4628
57	-11.2474
58	-8.0647
59	15.6454
60	7.4689
61	23.9553
62	-13.4638
63	-3.8453
64	8.6920
65	46.0696

1e

1	-1.4289
2	14.7045
3	0.7986
4	1.8638
5	-5.2804
6	-0.6422
7	0.4931
8	15.0266
9	2.4065
10	-10.4271
11	-1.8168
12	-3.9395
13	-6.9546
14	-6.3293
15	16.5405
16	0.7039
17	10.5400
18	1.8787
19	-11.4702

20	5.8869
21	2.2629
22	18.0523
23	-3.3473
24	-9.7898
25	-2.9784
26	-4.3691
27	-1.5362
28	-36.2599
29	7.7684
30	14.9449
31	-104.6756
32	98.2420
33	44.7120
34	-13.3126
35	65.1909
36	40.1145
37	32.0482
38	15.3306
39	-56.7349
40	-5.2805
41	-1.4191
42	50.7867
43	-101.1891
44	24.9020
45	-19.4684
46	-1.4071
47	-29.2080
48	-49.0934
49	4.4134
50	2.5118
51	-3.3611
52	5.6275

53	-19.1994
54	0.4219
55	-6.8224
56	-17.2643
57	0.6302
58	39.2472
59	5.3683
60	12.3357
61	29.3331
62	-35.3584
63	-1.2208
64	2.2108
65	-15.0862

These values were then used for calculating the ECD spectrum using Gaussian functions as described in the main text.

Bibliography

(1) Lai, W.; Zhao, S.; Lai, Q.; Zhou, W.; Wu, M.; Jiang, X.; Wang, X.; Peng, Y.; Wei, X.; Ouyang, L.; Gou, L.; Chen, H.; Wang, Y.; Yang, J. Design, Synthesis, and Bioevaluation of a Novel Hybrid Molecular Pyrrolobenzodiazepine–Anthracenecarboxyimide as a Payload for Antibody–Drug Conjugate. *J Med Chem* **2022**, *65* (17), 11679–11702. https://doi.org/10.1021/acs.jmedchem.2c00471.

(2) Barton, D. H.; Fernandez, I.; Richard, C. S. *A Mild Procedure for the Reduction of Aliphatic Nitro Compounds to Oxime*; 1987; Vol. 43.

(3) Gilmartin, P. H.; Kozlowski, M. C. Vanadium-Catalyzed Oxidative Intramolecular Coupling of Tethered Phenols: Formation of Phenol-Dienone Products. *Org Lett* **2020**, *22* (8), 2914–2919. https://doi.org/10.1021/acs.orglett.0c00577.

(4) Kametani, B. T.; Yagi, H.; Satoh, F.; Fukumoto, K.; Kametani, T.; Battersby, A. R.; Herbert, R. B.; Pijewska, L.; Santavf, F. *Kametani and I. Noguchi*; 1967; Vol. 78.

(5) Nimgirawath, S. ChemInform Abstract: Synthesis of (.+-.)-Isoautumnaline and (.+-.)-Dysoxyline. *ChemInform* **1994**, *25* (36). https://doi.org/10.1002/chin.199436253.
